# HRP2 and pLDH-Based Rapid Diagnostic Tests, Expert Microscopy, and PCR for Detection of Malaria Infection during Pregnancy and at Delivery in Areas of Varied Transmission: A Prospective Cohort Study in Burkina Faso and Uganda

**DOI:** 10.1371/journal.pone.0156954

**Published:** 2016-07-05

**Authors:** Daniel J. Kyabayinze, Issaka Zongo, Jane Cunningham, Michelle Gatton, Patrick Angutoko, John Ategeka, Yves-Daniel Compaoré, Atis Muehlenbachs, Jerry Mulondo, Miriam Nakalembe, Fabrice A. Somé, Aminata Ouattara, Noél Rouamba, Jean-Bosco Ouédraogo, Heidi Hopkins, David Bell

**Affiliations:** 1 Foundation for Innovative New Diagnostics, Kampala, Uganda; 2 Institut de Recherche en Sciences de la Santé (IRSS), Direction Régionale de l’Ouest, Bobo-Dioulasso, Burkina Faso; 3 UNICEF/UNDP/World Bank/WHO Special Programme for Research & Training in Tropical Diseases (TDR), Geneva, Switzerland; 4 School of Public Health and Social Work, Queensland University of Technology, Queensland, Australia; 5 Department of Pathology, University of Washington, Seattle, Washington, United States of America; 6 Department of Obstetrics & Gynecology, Makerere University College of Health Sciences, Kampala, Uganda; 7 Foundation for Innovative New Diagnostics, Geneva, Switzerland; Centro de Pesquisa Rene Rachou/Fundação Oswaldo Cruz (Fiocruz-Minas), BRAZIL

## Abstract

**Background:**

Intermittent screening and treatment (IST) of malaria during pregnancy has been proposed as an alternative to intermittent preventive treatment in pregnancy (IPTp), where IPTp is failing due to drug resistance. However, the antenatal parasitaemias are frequently very low, and the most appropriate screening test for IST has not been defined.

**Methodology/Principal Findings:**

We conducted a multi-center prospective study of 990 HIV-uninfected women attending ANC in two different malaria transmission settings at Tororo District Hospital, eastern Uganda and Colsama Health Center in western Burkina Faso. Women were enrolled in the study in the second or third trimester of pregnancy and followed to delivery, generating 2,597 blood samples for analysis. Screening tests included rapid diagnostic tests (RDTs) targeting histidine-rich protein 2 (HRP2) and parasite lactate dehydrogenase (pLDH) and microscopy, compared to nPCR as a reference standard. At enrolment, the proportion of pregnant women who were positive for *P*. *falciparum* by HRP2/pan pLDH RDT, Pf pLDH/pan pLDH RDT, microscopy and PCR was 38%, 29%, 36% and 44% in Uganda and 21%, 16%, 15% and 35% in Burkina Faso, respectively. All test positivity rates declined during follow-up. In comparison to PCR, the sensitivity of the HRP2/pan pLDH RDT, Pf pLDH/pan pLDH RDT and microscopy was 75.7%, 60.1% and 69.7% in Uganda, 55.8%, 42.6% and 55.8% in Burkina Faso respectively for all antenatal visits. Specificity was greater than 96% for all three tests. Comparison of accuracy using generalized estimating equation revealed that the HRP2- detecting RDT was the most accurate test in both settings.

**Conclusions/Significance:**

The study suggests that HRP2-based RDTs are the most appropriate point-of-care test currently available for use during pregnancy especially for symptomatic women, but will still miss some PCR-positive women. The clinical significance of these very low density infections needs to be better defined.

## Introduction

The negative effects of malaria infection in pregnancy have been long recognized [1–4]. Placental malaria infection, while often asymptomatic, has been clearly associated with poor outcomes including maternal anemia, low birth weight, infant mortality and delayed child development [5–8]. Because of these dangers, the World Health Organization (WHO) recommends, and many African countries implement, intermittent preventive treatment in pregnancy (IPTp) with sulfadoxine-pyramethamine (SP) in addition to other measures including long-lasting insecticide-treated nets (LLINs) and effective case management. However, as SP resistance rises, there is uncertainty over the effectiveness of SP for IPTp and alternative strategies are needed [9]. The alternatives, artesunate-based combination therapy (ACT) or quinine, are not recommended in pregnancy without first demonstrating the presence of infection.

A challenge of screening and ACT-based treatment in pregnancy is that *P*. *falciparum* malaria parasites, sequester in the placenta and may not be detected in maternal peripheral blood by routine laboratory testing, particularly microscopy[10]. Alternative diagnostic methods that do not rely on direct visualization of parasites may enable more reliable detection of malaria infection during gestation. Detection of parasite antigen in peripheral blood by RDTs offers an alternative[11]. Antigens detected by currently available RDTs include histidine-rich protein 2 (HRP2), which persists in circulation for a number of weeks and so can accumulate, and parasite lactate dehydrogenase (pLDH) that clears rapidly. The differing properties of these targets may render them more or less suited as screening tests in different environments and populations[12–13].

A prospective study was conducted to evaluate the performance of rigorously quality-controlled malaria diagnostic tests during gestation and at delivery, in sites with different malaria prevalence, to determine the potential utility of RDTs to detect and treat malaria infection during pregnancy. This report compares results of different diagnostic tests performed on maternal peripheral blood during pregnancy and at delivery.

## Materials and Methods

### Ethics and protocol

All participants provided their written informed consent to participate in the study before any study procedures were performed by the study staff member. When a participant could not read and write, an impartial adult witness confirmed that the mother had participated in the informed consent discussion, had understood the contents of the consent form, and freely agrees to participate. Additional consent was also obtained for future use of biological samples. The ethics committee approved this consent procedure.

The study was approved by the following ethics review committees: a) World Health Organization Research Ethics Review Committee (protocol ID RPC390), b) Comité d’Ethique Institutionnel of the Centre Muraz of the Ministère de la Santé in Burkina Faso (reference number A23-2010/CE-CM), c) School of Medicine Research and Ethics Committee of Makerere University in Uganda (reference number 2011–046), and d) Uganda National Council of Science & Technology (reference number HS160)(S1 and S2 Files). This report conforms to STROBE (S1 Table) guidelines for reporting results of observational cohort studies[14] and STARD guidelines for studies of diagnostic accuracy[15].

### Study setting and population

The study was conducted at the Colsama health center in the District de Dô, a peri-urban area of Bobo-Dioulasso in southwestern Burkina Faso, and at the Tororo District Hospital in south-eastern Uganda. Both sites are government-sponsored health facilities that provide routine antenatal and delivery care, including: provision of LLINs, provision of IPTp-SP at least twice during pregnancy according to the standard of care at the time of the study [16], treatment of symptomatic malaria in pregnancy with quinine (Burkina Faso) or ACTs (Uganda), and testing and care for HIV-infected women.

Malaria transmission in the region of Bobo-Dioulasso is highly seasonal peaking from June to October, with an estimated *P*. *falciparum* entomological inoculation rate of 300–500 infective bites per person per year [17]. Tororo region is holoendemic area for malaria, with an entomological inoculation rate (EIR) estimated at 310 infective bites per person per year [18]. The prevalence of malaria among pregnant women in the region was reported to be approximately 40% based on PCR-corrected microscopy[19]. In both sites, *P*. *falciparum* is the dominant malaria species.

Specific participant inclusion criteria were:

1. Presenting for care after quickening and before onset of labor (i.e. in the second or third trimester of pregnancy)
2. Age between 16 years and 44 years
3. Willingness and ability to follow up with study visits through the duration of pregnancy and at delivery
4. Absence of history of serious adverse reaction to sulfa-containing drugs
5. Absence of history of serious adverse reaction to artemisinin-based drugs (Uganda) or quinine (Burkina Faso)
6. Absence of HIV infection
7. Absence of history of, or current, obstetric complications (e.g. pre-eclampsia, eclampsia, hypertension during pregnancy, post-partum hemorrhage, current evidence of multiple gestation)
8. Absence of chronic disease (including diabetes mellitus and sickle cell disease)
9. Absence of severe disease requiring inpatient management or referral
10. Provision of written informed consent
11. Absence of severe anaemia (Hb ≥7 g/dL)

### Sample size

The target sample size at each study site was calculated to test hypotheses based on the positive and negative predictive values (PPV and NPV, respectively). The null hypothesis was: [PPV ≤ (disease prevalence + 0.4) or NPV ≤ (1-disease prevalence)] with 80% power and 5% significance at each study location. Previously published estimates of RDT sensitivity (65%) and specificity (98%) were used in these calculations and malaria prevalence was assumed to be 15% in each location. Based on these estimates, an enrollment target was set of 345 women in Uganda and 345 women in Burkina Faso, to ensure 90% confidence of obtaining the required number of positive and negative tests, and allowing for a 15% loss to follow-up between enrolment and delivery. To meet secondary study objectives involving clinical outcomes (not reported here), a larger target sample size was set in Burkina Faso.

### Study visits

**Fig 1:**
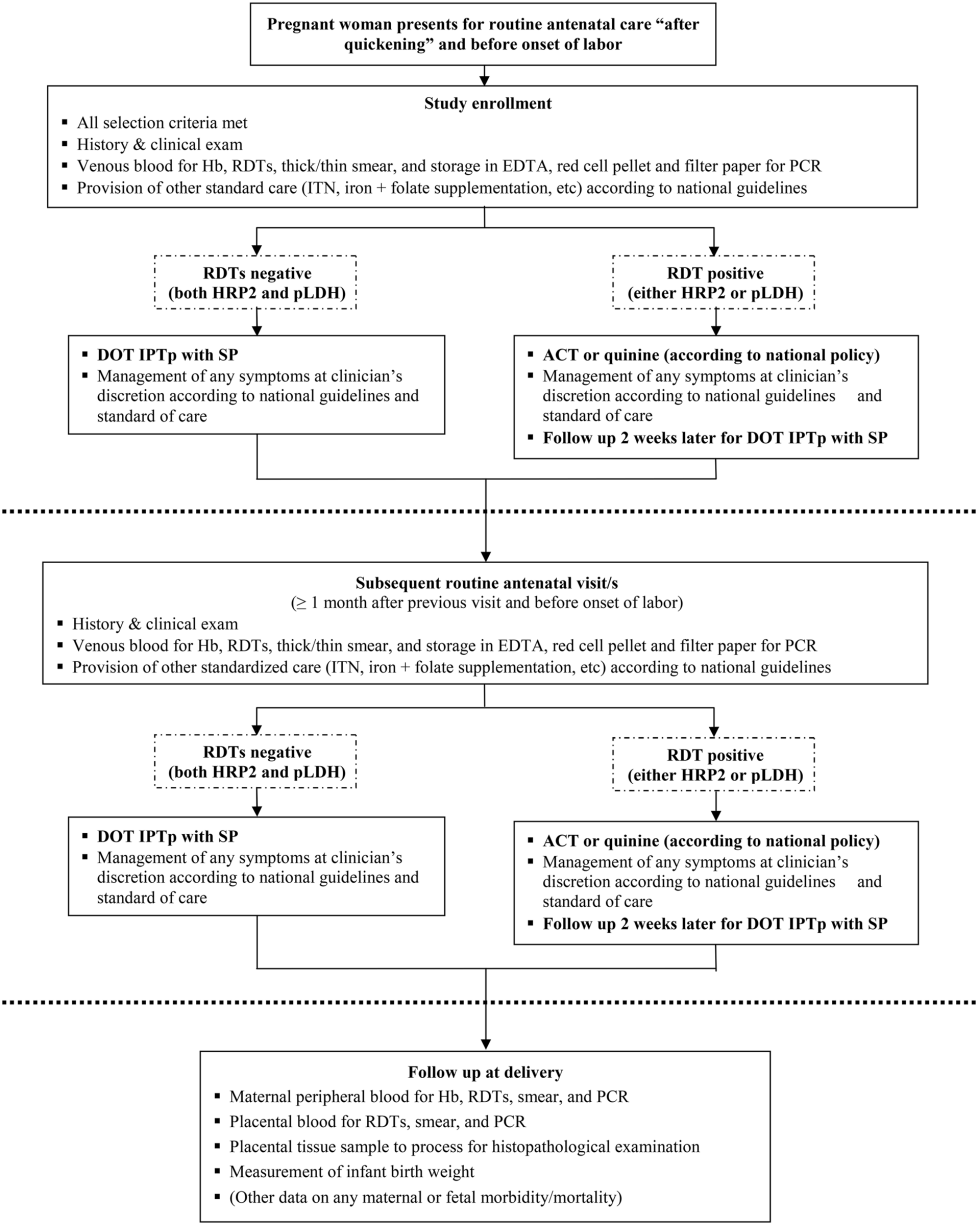
Flow diagram of the study participants.

Study activities ran from November 2010 to April 2012 at the Burkina Faso site and from May 2011 to April 2012 at the Uganda site. Potential participants were referred to the study team by the antenatal clinic staff at each site; study staff completed screening and enrollment consecutively, according to the selection criteria shown in Fig 1. Subsequent antenatal visits were scheduled at least four weeks apart. Participants were encouraged to return to the study site in case of illness between scheduled visits, and to attend the study site for delivery.

### Sample collection

At each scheduled visit and at delivery, a focused physical examination was conducted and 2 mL venous blood sample was drawn for measurement of hemoglobin (Hb; HemoCue, Quest Diagnostics, Ängelholm, Sweden), preparation of RDTs and blood smears, and storage in a microtainer at -20°C and on filter paper. After delivery, placental blood and a placental biopsy were collected; the complete methods and the resulting findings will be reported separately. If a participant missed a visit then a home visit was arranged, and study staff attempted to visit the homes of those who delivered elsewhere within 24 hours of the birth.

### Provision of antenatal and delivery care

At enrollment and at each scheduled visit, RDT results were used to guide management. When all RDT test bands were negative, the participant took a directly observed scheduled dose of SP. When any RDT test band was positive, the participant received treatment with quinine (600mg, eight hourly for 7 days) in Burkina Faso or artemether-lumefantrine (AL) (80/480 mg twice daily for three days) in Uganda; the initial dose was directly observed, and the remaining doses were given to the participant with clear instructions for completing the treatment at home. If quinine or AL was administered, study staff visited the woman at home two weeks later to administer a dose of SP as IPTp. If a participant presented with symptoms suggestive of malaria, microscopy was performed immediately by study staff and microscopy results (rather than RDT) were used for clinical management according to national policy. All study participants received routine antenatal care according to national guidelines, including receipt of a LLIN, iron and folate supplementation, and management of any other symptoms at the treating clinician’s discretion.

### Diagnostic test procedures

#### Rapid diagnostic tests (RDTs)

RDT kits for this study were selected on the basis of their performance in WHO malaria RDT product testing and the target antigens detected[20]. The study selected the three most commonly used antigen-detection targets: HRP2, pan-specific pLDH (pan-pLDH) and *P*. *falciparum-*specific pLDH (Pf-pLDH). Two combination tests comprising four test bands were used: CareStart^™^ Malaria pLDH (Pf/PAN) Test, product G0121, lot E10IL, expiry April 2012 (here referred a pLDH); and CareStart^™^ Malaria HRP2/pLDH (Pf/PAN) COMBO Test, product G0131, lot E10IR, expiry April 2012 (here referred a HRP2). RDTs were procured directly from the manufacturer, Access Bio, Inc. (Monmouth Jct, New Jersey, USA).

Before the study began, RDTs passed lot testing according to WHO-FIND guidelines[20] at the Centers for Disease Control and Prevention (CDC), Atlanta, Georgia, USA. At the study sites, temperature and humidity of the storage areas for RDTs were not controlled but were monitored and remained within the manufacturer’s recommended storage conditions of 4°C to 40°C. After the completion of participant follow-up, RDTs were retrieved from both sites and again subjected to lot performance testing at the CDC.

RDTs were performed on maternal venous blood according to manufacturers’ instructions. Results of all four test bands were recorded. Invalid RDT results were repeated using the same blood sample. RDT results were reported to the treating clinician to guide participant management according to the study protocol.

#### Microscopy

Thick and thin blood smears were prepared and stained with 2% Giemsa for 30 minutes. A smear was declared negative if examination of 100 high power fields did not reveal asexual parasites or gametocytes. For positive smears, parasite density was calculated by counting the number of asexual parasites per 200 leukocytes (or per 500, if the count is <10 asexual parasites/200 leukocytes), assuming a leukocyte count of 8,000/μl. When a thick smear was positive, the corresponding thin blood smear was read to determine the parasite species. Each smear was read by two microscopists who were blinded to the RDT results and any previous microscopy. Results were considered discordant if one result was negative and the other positive for malaria infection; for positive smears, if the density estimates differed by >50% of the maximum reading; or if the two microscopists reported different species present. Discordant results were resolved by a third microscopist, taking into account concordance with the initial readings[21].

Before the study began, all microscopists were pre-qualified using WHO slide banks and procedures [22] and required to reach Level 1 or 2 expertise level. In addition, a random sample of 10% of slides from each site was sent for review at University of Lagos by Level 1 expert microscopists.

#### Polymerase Chain Reaction (PCR)

For all routine antenatal visits, 2.5 mL of peripheral blood was collected into an EDTA microtainer, labeled and stored at -20°C. Stored samples were transferred to a central laboratory (at Institut de Recherche en Sciences de la Santé [IRSS] in Bobo-Dioulasso, Burkina Faso) and analyzed by nested PCR to confirm presence or absence of parasites, and parasite species. PCR procedures were performed according to standardized protocols and published methods[23]. PCR technologists were blinded to RDT and microscopy results.

Before the study began, proficiency testing of the IRSS molecular laboratory was performed using reference samples from the CDC. In addition, a stratified random sample of 5% of samples were re-tested at the Institut Pasteur du Cambodge laboratory in Phnom Penh, Cambodia, as a means of external quality control.

### Data management and statistical analysis

Test outcomes and clinical data were collected on case record forms and were double-entered in an electronic database using Epi-Info version 6.04 (Centers for Disease Control and Prevention, Atlanta, GA). Data were subsequently cleaned and analyzed using SPSS version 16.0. Data from the antenatal visits (655 samples from Uganda and 1087 samples from Burkina Faso) were used to calculate sensitivity, specificity, positive predictive value (PPV) and negative predictive value (NPV) for each diagnostic test. Generalized Estimating Equations (GEEs) were used to compare the sensitivity, specificity, PPV and NPV between diagnostics, assuming that PCR is the gold standard for the presence of *P*. *falciparum*. Explanatory variables including age, gravidity, prior antimalarial treatment or IPTp, fever symptoms within last 24 hours, were tested for their influence on diagnostic performance.

## Results

### Study participants baseline demographics

Six hundred and fifty (650) pregnant women in Burkina Faso and 340 in Uganda were enrolled, of whom 85% and 90% respectively returned for delivery (Fig 1). Half received their first IPTp-SP dose beyond 28 weeks of amenorrhea in Burkina Faso, while this proportion was three quarters in Uganda. Other baseline characteristics and comparisons between the two sites are shown in Table 1.

**Table 1:** Demographic characteristics of enrolled pregnant women from antenatal clinics in Tororo District Hospital, Uganda and Colsama Health Center, Burkina Faso.

Demographic characteristic	Burkina Faso N = 650	Uganda N = 340	Comparison of sites
Age: median (IQR)	24 (20–29)	23 (20–27)	P = 0.001*
Gravidity: median (IQR)	3 (1–4)	2 (1–3)	
Primigravidae	170 (26.2%)	115 (33.8%)	P = 0.012*
Fundal height cm- median(IQR)	23 (21–25)	24 (20–27)	
Participants with fever symptoms	68 (10.5%)	47 (14.0%)	
Prior IPTp at enrolment (n, %)	107 (16.5%)	39 (11.5%)	P = 0.038*
Prior antimalarial treatment	199 (30.6%)	43 (12.6%)	P = 0.001*

* statistically significant difference between the two study populations (distributions by Mann-Whitney U test, proportions by chi square test)

### Diagnostic test results

The two different RDTs containing HRP2/pan pLDH RDT is hereby referred to as HRP2 kit and the second kit containing Pf pLDH/pan pLDH RDT is reported as pLDH for ease of reading. At enrolment in Burkina Faso, the proportion of samples positive based on PCR for *P*. *falciparum*35%, HRP2 RDT 21% Pf pLDH 16% RDT and expert microscopy 15%. In Uganda these proportions were 44% for PCR, 38% for HRP2, 29% pLDH and 36% microscopy (Table 2). The proportions positive for all diagnostics test were lower for subsequent and delivery samples at each site. Overall PCR and the HRP2 based RDT detected more infections than the Pf-pLDH/pan pLDH RDT and expert microscopy. Considering the individual test bands of each RDT, the pan pLDH test band returned fewer positive results than either the HRP2 or Pf pLDH test bands. In Burkina Faso the pan pLDH band was positive in 76% and 12% of positive RDT results for the HRP2/pan pLDH RDT and the Pf pLDH/pan pLDH RDT, respectively. In Uganda these values were 84% and 37%.

**Table 2:** Diagnostic test results and species of samples of pregnant women from Burkina Faso and Uganda.

	Number (%, n) of positive results	
	Burkina Faso	Uganda
**Enrolment Visits**		
Pf PCR	224 (34.5%, 649)	149 (44.0%, 338)
HRP2/pan pLDH RDT	134 (20.6%, 650)	130 (38.2%, 340)
Pf pLDH/pan pLDH RDT	102 (15.7%, 650)	99 (29.1%, 340)
Microscopy	95 (14.6%, 650)	123 (36.2%, 340)
**Subsequent visits**		
Pf PCR	25 (5.7%, 438)	70 (22.1%, 317)
HRP2/pan pLDH RDT	11 (2.5%, 438)	54 (17.0%, 317)
Pf pLDH/pan pLDH RDT	7 (1.6%, 438)	40 (12.6%, 317)
Microscopy	7 (1.6%, 437)	43 (13.6%,317)
**Delivery visit**		
Pf PCR	26 (5.7%, 555)	88 (29.7%, 296)
HRP2/pan pLDH RDT	9 (1.6%, 555)	67 (22.9%, 297)
Pf pLDH/pan pLDH RDT	9 (1.6%, 555)	39 (13.3%, 297)
Microscopy	9 (1.6%, 554)	49 (16.7%, 294)
**Species distribution of all PCR-positive samples**		
*P*. *falciparum*	264 (93.0%)	272 (84.0%)
*P*. *ovale*	9 (3.2%)	9 (2.8%)
*P*. *malariae*	0	8 (2.5%)
Mixed (Pf. + other)	11 (3.9%)	35 (10.8%)
Total samples +ve by PCR	284	324

Seasonal patterns in test positivity at enrollment are shown in S1 Fig. The graph shows variation in PCR-detected infection in Burkina Faso from just under 20% at the end of the dry season to about 55% toward the end of the rainy season; and a similar range in Uganda.

*P*. *falciparum* was the predominant species at both sites across all visits as determined by PCR (Table 2). In Burkina Faso, the majority of samples (275, 97%) contained *P*.*falciparum*, while 3% were mono-infections with *P*. *ovale*. In Uganda *P*. *falciparum* was found in 95% of samples, either alone (84%) or in combination with P. *ovale* or *P*. *malariae*. PCR-determined mono-infection with *P*. *ovale* and *P*. *malariae* were recorded for 9 samples from Burkina Faso and 13 samples from Uganda. Neither of the RDTs used returned positive results for these samples. Further analysis is restricted to consideration of *P*. *falciparum* only (including mixed infections).

Estimates of parasite density at enrolment were obtained from microscopy readings. In Burkina Faso 95/650 (14.6%) of women were parasite positive by microscopy. For this group of women the geometric mean parasite density was 788 parasites/μL (median = 780 parasites/μL, IQR = 279.5–2,349 parasites/ μL). Thirty-six percent (123/340) of women in Uganda were parasite positive by microscopy at enrolment. For this group of women the geometric mean parasite density was 371 parasites/μL (median = 348 parasites/μL, IQR = 72–1,508 parasites/ μL). Although a smaller proportion of women were microscopically positive at enrolment in Burkina Faso compared to Uganda, these women presented with higher parasite densities (P = 0.002, Mann-Whitney test).

At enrolment, 68/650 (10.5%) of participants had fever symptoms in Burkina Faso. There was no association between having febrile symptoms and returning at least one positive diagnostic tests (P = 0.894, Fisher’s Exact test). In Uganda, 47/340 (13.8%) of participants had fever symptoms and these participants were more likely to return at least one positive diagnostic result, compared to participants without febrile symptoms (P<0.001, Fisher’s Exact test).

At enrollment, 15% and 41% of positive samples were positive only by PCR in Uganda and Burkina Faso, respectively. However, among the subsequent visit and delivery visit samples the proportion of samples that were positive by PCR only increased to 25% and 27% in Uganda, and to 55% and 58% in Burkina Faso, respectively. Therefore, the proportion of sub-patent infections—i.e. infections below the level of detection of RDTs and microscopy but positive by PCR—was higher at subsequent and delivery visits than at enrollment visits (S1 Fig).

### Diagnostic test results compared with PCR as reference standard

In the antenatal samples from Burkina Faso, the HRP2 had a significantly higher sensitivity for *P*. *falciparum* than either microscopy or the Pf-pLDH/pan pLDH RDT (P<0.01; Table 3). There was no difference between the sensitivities of microscopy and the pLDH (p>0.1). Specificity values on these same samples were high (>99%) with no significant differences between the three diagnostic tests (P>0.3, Table 3). There were also no differences between the PPV of the three diagnostics (P = 0.085, Table 3). Considering NPV, microscopy and the Pf-pLDH/pan pLDH RDT had similar values (P = 0.194), while the HRP2had a significantly higher NPV for *P*. *falciparum* (P<0.001).

**Table 3:** Accuracy of diagnostics for detection of *P*. *falciparum* in peripheral maternal blood during antenatal visits and delivery visits.

	Burkina Faso (PCR positive 249 / 1087) Statistic (95% CI)			Uganda (PCR positive = 218 / 655) Statistic (95% CI)		
	Microscopy	HRP2/pan pLDH RDT	Pf pLDH/pan pLDH RDT	Microscopy	HRP2/pan pLDH RDT	Pf pLDH/pan pLDH RDT
**Antenatal visits**						
Sensitivity	39.8 (34.1–46.3)	55.8 (50.0–62.4)	42.6 (36.9–49.2)	69.7 (63.9–76.1)	75.7 (70.2–81.6)	60.1 (53.9–67.0)
Specificity	99.8 (99.1–99.9)	99.3 (98.4–99.7)	99.6 (98.9–99.9)	98.4 (96.7–99.2)	95.7 (93.3–97.2)	98.2 (96.4–99.1)
PPV (%)	98.0 (92.2–99.5)	95.9 (90.9–98.1)	97.3 (91.6–99.1)	95.6 (90.9–97.9)	89.7 (84.2–93.3)	94.2 (87.7–97.1)
NPV (%)	84.8 (82.3–86.8)	88.3 (86.1–90.2)	85.4 (83.0–87.4)	86.7 (83.3–89.4)	88.6 (85.5–91.3)	83.1 (79.6–86.1)
**Delivery visit**						
Sensitivity (%)	34.6 (20.4–58.7)	34.6 (20.4–58.7)	34.6 (20.4–58.7)	54.0 (44.5–65.6)	67.0 (57.9–77.6)	42.1 (32.9–53.7)
Specificity (%)	100.0 (99.3–100.0)	100.0 (99.3–100.0)	100.0 (99.3–100.0)	99.5 (96.6–99.9)	96.2 (92.4–98.1)	99.0 (96.2–99.8)
PPV (%)	100.0 (66.2–100.0)	100.0 (66.2–100.0)	100.0 (66.2–100.0)	97.9 (85.5–99.7)	88.1 (77.1–93.8)	94.9 (80.2–98.7)
NPV (%)	96.9 (95.0–98.1)	96.9 (95.0–98.1)	96.9 (95.0–98.1)	84.7 (80.4–89.6)	86.0 (81.8–90.4)	81.7 (77.0–86.7)

In Uganda, the sensitivity and NPV of the HRP2on antenatal samples were significantly higher than expert microscopy, which were significantly higher than the pLDH (P<0.02, Table 3). The specificities for the three tests were above 95%, however the HRP2had a significantly lower value than the other two diagnostics (P<0.05, Table 3). The HRP2 kit had the lowest PPV of 89.7%. This value was significantly lower than microscopy (P = 0.006), but was not significantly different to the pLDH (P>0.05, Table 3).

Both RDTs returned false positive results for *P*. *falciparum* infection using PCR as the reference standard. In Burkina Faso the false positive rates (false positives/all positives) were 0.24% and 0.06% for the HPR2/pan pLDH RDT and pLDH respectively (n = 1639). In Uganda the false positive rates were higher at 2.22% and 0.63%, respectively (n = 948). Interestingly, one enrolment sample in Uganda was positive on both RDTs, but negative by PCR and microscopy. There was no pattern in the false positive rates between enrolment, subsequent visit and delivery samples. There were no false positive results for microscopy.

### Factors associated with test accuracy on antenatal samples

Data from the antenatal samples in Uganda and Burkina Faso were merged to investigate factors that affected the diagnostic performance of microscopy, and the two RDTs. At total of 1,742 samples were available for analysis in this combined data set and the factors considered were age of woman, gravidity, whether the women had received prior antimalarial treatment or IPTp during the pregnancy, whether fever symptoms where present or reported within 24 hours of the sample collection, and country. Country interactions were also considered. The temporal pattern of transmission differed between Uganda and Burkina Faso so month of visit was not included in the models as an explanatory variable. Hence the effect of seasonality was not assessed in the combined data set (Table 4). The rainy season in Burkina Faso is between June and October. Uganda has two rain seasons, December to February and again between May to July.

**Table 4:** Fitted models predicting diagnostic performance on antenatal samples in Uganda and Burkina Faso.

Parameter	Explanatory variable		Relative Risk for explanatory variable (95% CI)		
			Microscopy	HRP2 / pan(pLDH) RDT	Pf pLDH / pan(pLDH) RDT
Sensitivity	Baseline value*		91.8%	92.0%	74.2%
	Gravidity		0.85 (0.80–0.91)	0.90 (0.85–0.94)	0.83 (0.77–0.90)
	Prior trmt	No	1.00	1.00	1.00
		Yes	0.80 (0.69–0.93)	0.87 (0.77–0.98)	NS
	Country	Uganda	1.00	1.00	1.00
		Burkina Faso	0.61 (0.52–0.72)	0.77 (0.69–0.87)	0.78 (0.66–0.92)
Specificity	Baseline value*		97.3%	91.1%	96.0%
	Age		NS	1.13 (1.02–1.25)	NS
	Prior trmt	No	1.00	1.00	1.00
		Yes	16.13 (1.98–125.00)	3.00 (1.42–6.33)	10.75 (2.79–41.67)
	Symptoms	No	1.00	1.00	1.00
		Yes	0.18 (0.05–0.65)	0.25 (0.12–0.52)	0.28 (0.08–0.97)
	Country	Uganda	1.00	1.00	1.00
		Burkina Faso	4.85 (1.00–23.26)	5.32 (2.20–12.82)	4.41 (1.16–16.67)
Negative Predictive Value	Baseline value*		75.6%	80.8%	72.9%
	Age		1.04 (1.00–1.07)	1.04 (0.99–1.08)	1.05 (1.02–1.09)
	Gravidity		0.88 (0.80–0.98)	0.87 (0.78–0.98)	0.86 (0.78–0.94)
	Prior trmt	No	1.00	1.00	1.00
		Yes	2.87 (2.23–3.69)	2.84 (2.11–3.82)	3.10 (2.43–3.96)
	Country	Uganda	1.00	1.00	1.00
		Burkina Faso	NS	NS	NS

* Estimated marginal mean of parameter for woman in Uganda who is primigravidae, aged 20 years, has had no prior treatment and has no fever symptoms. NS, Not significant (P>0.05)

#### Sensitivity

The sensitivity of all three diagnostics was significantly influenced by gravidity (P<0.05), while prior treatment/IPTp was important for microscopy and the HRP2 (P<0.05), but not the pLDH (P>0.05). Sensitivity decreased with increasing gravidity, and also with prior treatment/IPTp. Country was a significant factor for sensitivity for all diagnostics (P<0.05), even after accounting for gravidity and prior treatment/IPTp, with Uganda having higher sensitivity than Burkina Faso.

#### Specificity

For all three diagnostics, specificity was significantly influenced by prior treatment/IPTp, the presence of fever symptoms and country (P<0.05). The presence of fever symptoms and/or having no prior treatment/IPTp was associated with lower specificity. For the HRP2, age of woman was also a significant explanatory variable (P<0.05); specificity increased with age.

#### Positive Predicative value

None of the explanatory variables tested were significant in the model to predict PPV (P>0.05).

#### Negative Predicative Value

Age, gravidity and prior treatment/IPTp were found to influence the NPV in all three diagnostics (P<0.05). The largest influencing factor was prior treatment/IPTp, with samples from women having prior treatment/IPTp having a higher NPV. The influence of age and gravidity was most obvious in samples from women who had no prior treatment/IPTp with increasing age and decreasing gravidity associated with higher NPV. For the samples collected in Burkina Faso, month of visit was also a significant factor for NPV for all three diagnostics, with lower NPV in the high transmission season (Oct-Jan).

#### Parasite Density

The diagnostic positivity rate for the different tests on enrolment samples was influenced by parasite density. The number of diagnostic tests positive for a sample increased with increasing parasite density (P<0.001, Mann-Whitney U test). All samples with a microscopy-determined density above 1,000 parasites/μL were positive on at least three of the diagnostic tests.

## Discussion

Malaria diagnostic testing of pregnant women may allow better targeting of efficacious antimalarial treatment to asymptomatic women with demonstrated malaria infection, as a replacement for failing IPTp. In this study, we screened asymptomatic pregnant women with quality-assured RDTs in two African clinical settings (Uganda, high transmission, and Burkina Faso, seasonal transmission) and treated those with positive results. In parallel, microscopy and PCR were conducted. We determined the accuracy of the various diagnostics using PCR as the reference standard in these two distinct settings. Diagnostic testing of pregnant women who receive IPTp detected high proportions of malaria infection. Many of these infections were of low density and only detected by PCR, rather than HRP2/pan pLDH and Pf pLDH/pan pLDH RDTs and microscopy.

Close to half of all the women in the higher transmission setting had detectable malaria infection but only 14% reported fever or history of fever symptoms at the time of enrolment. In the seasonal transmission setting, fewer (35%) women had detectable parasites at enrolment, with 10% reporting fever or history of fever. Overall, PCR detected 32%, 49% and 47% more positive samples then the HPR2/pan pLDH RDT, the pLDH and expert microscopy, respectively. The higher sensitivity of the HRP2-detecting RDT may reflect the detecting of circulating antigen from parasites confined to the placenta, or recent spikes in parasite density. While it is reasonable to postulate that higher parasite densities in circulation may cause greater harm to mother and child, a greater understanding of the clinical significance of the third of PCR-positive women who were undetectable by the most sensitive RDT (and so would not receive treatment under a RDT-based screening approach) is needed.

Age, gravidity, fever symptoms, prior antimalarial treatment or IPTp, and month of visit were all significant explanatory factors for variations in sensitivity, specificity, PPV, NPV, although the significance of associations varied with diagnostic test and country. The lower prevalence of infection in older and more gravid women likely reflects increasing immunity, though the differences in prevalence in this age range should be relatively low. As expected, all three point- of-care diagnostics were of high sensitivity and lower specificity in women with fever symptoms—these cases are expected to have higher parasite density. Parasite density is known to influence the performance of malaria diagnostics; however, parasite density itself was not assessed as a factor in the statistical models since densities were only available for microscopy-positive samples. The presence of fever symptoms and prior treatment/IPTp were also used as surrogate measures for parasite density. The association between the explanatory variables and diagnostic performance suggests that some measures of performance are significantly influenced by immunity and parasite density. The significant country effect for sensitivity and specificity is likely linked to infection rates, as the Ugandan site has a higher incidence and multi-clonal infections and recrudescence of infection is probably greater, promoting higher parasite loads. Transmission in Burkina Faso is highly seasonal which was shown to impact on NPV, but seasonality itself did not affect PPV, sensitivity or specificity. These results indicate that the epidemiological background and source of samples are important considerations when diagnostic performance is being assessed. Of particular interest for antenatal screening are the differences noted for prior treatment/IPTp. It may be that prior treatment/ IPTp was frequently successful in clearing infection, and these patients, when positive, tended to have new infections with higher parasite densities, and so were more readily detectable.

The advantage of HRP2-detecting RDTs over microscopy, presumably reflecting the detection of sequestration of parasites in the placenta, is contrary to what some previous studies have reported [24]. In this study we believe microscopy was of very high quality, and expert microcopy conducted in Uganda was approximately similar to the HRP2 in the proportion of positive samples; and expert microscopy in Burkina Faso was approximately similar to the Pf-pLDH/pan pLDH RDT. However, these levels of expert reading are not available for daily health care in antenatal setting, and it is likely that microscocpy-based point-of-care screening at antenatal visits would commonly be poorer when performed on a routine basis.

False positive (FP) rate was higher in HRP2 than pLDH RDTs, but the reason for this is not clear in the absence of prior treatment. Other previous studies showed that the difference was due to persistent antigenicity [25]. However, since the FP rate was lower post –treatment, this suggests it may not be persistent HRP2 antigenicity, but actually true positives missed by the reference test (PCR).

While evidence is lacking as to whether the 25–50% RDT-missed PCR-positive infections are clinically significant [26], many stakeholders may consider these RDTs insufficiently sensitive for effective use in antenatal screening programs. However, in many areas, declining malaria transmission and/or increasing SP resistance may force a change in IPTp strategies as it becomes difficult to justify or ineffective. Currently, achieving greater sensitivity requires the use of a nucleic acid amplification test (NAAT) assay, at significantly greater cost, and the clinical benefit may be small. As IPTp is reduced, programmes will have to make this cost-benefit trade-off. A recent study showed that by improving the diagnostic sensitivity to 20 parasites per microlitre increased the proportion of detection of infection by 49% in Burkina Faso[27].

Screen testing for malaria during the first antenatal visit detected the highest proportion of parasite infections, and at fairly similar levels in both Burkina Faso and Uganda. Infection rates reduce greatly after initial screening by RDT and treatment. This suggests that a screen and treat approach may be an effective alternative to IPTp, however the study design which included IPTp could not exclude the possibility that the reduction in prevalence was due, at least in some part, to the IPTp. However, the drop in infection rates in subsequent visits, indicating the impact of IPTp and treatment, was far greater in the seasonal malaria setting of Burkina Faso than in Tororo in Uganda where transmission is high and perennial. This likely reflects both rapid re-infection and higher rates of SP resistance /poorer prophylactic effect in Uganda.

The strengths of this study were the rigorous quality control of diagnostic methods and testing. Additionally, the prospective study design allowed us to observe pregnancy over time. The administration of IPTp to women 2 weeks after treatment, an ethical requirement, made it difficult to separate the impact of screening and treatment of confirmed cases from any beneficial effects of IPTp. Detailed clinical and histopathological assessment and longer follow-up post-delivery would inform the impact of the infections missed by RDT. Further data analysis from the study will assess this.

## Conclusions

The HRP2-based RDT appeared to perform better than Pf pLDH-based RDT and expert microscopy, in detecting malaria infection. All three diagnostics failed to detect some infections which were positive by PCR. Infection rates reduce greatly after initial screening by RDT and treatment. This suggests that a screen and treat approach may be an effective alternative to IPTp. Although HRP2 is not perfect, this study provides robust data on performance to inform decisions/tradeoffs in future setting like in lower transmission. Our results may inform policy decisions on alternatives to IPTp, especially in areas where declining malaria transmission and/or increasing SP resistance force a change.

## Supporting Information

S1 Fig Figure showing comparison of diagnostic test results at different study times. Positivity of diagnostic tests over the calendar year for PCR, RDTs (HRP2 and *p*LDH based kits) and microscopy showing then number of samples positive at time of enrolment (top), subsequent visits during antenatal (middle), and time of delivery (bottom) in Burkina Faso and Uganda.
(TIF)

S1 File Study protocol for Uganda in English. (DOC)

S2 File Study protocol Burkina Faso in French. (DOC)

S1 Table STROBE Checklist for reporting observational studies. (DOCX)
