# Supplementary figures and images for: HRP2 and pLDH-Based Rapid Diagnostic Tests, Expert Microscopy, and PCR for Detection of Malaria Infection during Pregnancy and at Delivery in Areas of Varied Transmission: A Prospective Cohort Study in Burkina Faso and Uganda
Source: PLoS One. 2016 Jul 5;11(7):e0156954. doi: 10.1371/journal.pone.0156954 (PMC4933335; doi:10.1371/journal.pone.0156954)

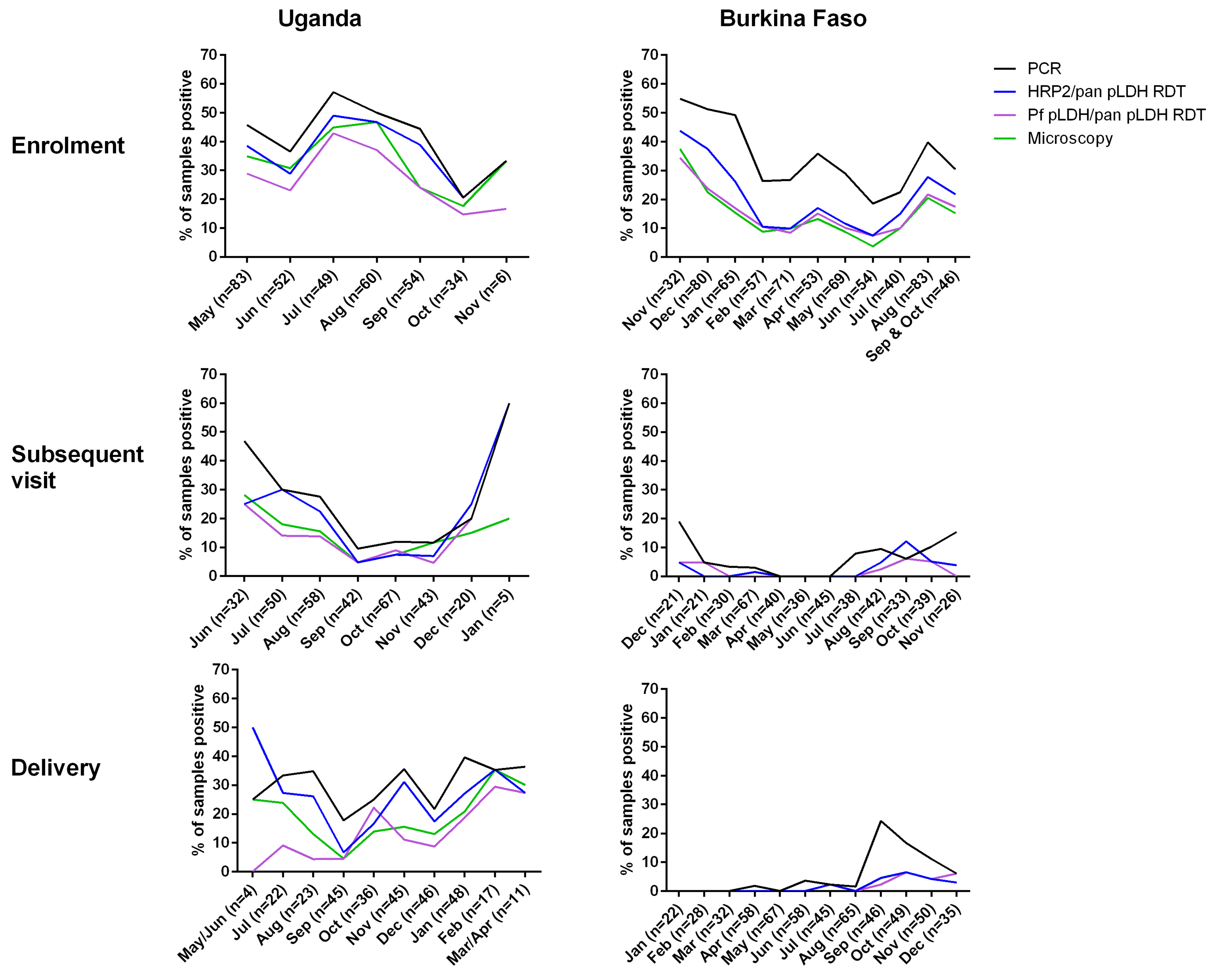

Supplement: S1 Fig — Positivity of diagnostic tests over the calendar year for PCR, RDTs (HRP2 and pLDH based kits) and microscopy showing then number of samples positive at time of enrolment (top), subsequent visits during antenatal (middle), and time of delivery (bottom) in Burkina Faso and Uganda. (TIF) [file pone.0156954.s001.tif]
