# Supplementary material for: HRP2 and pLDH-Based Rapid Diagnostic Tests, Expert Microscopy, and PCR for Detection of Malaria Infection during Pregnancy and at Delivery in Areas of Varied Transmission: A Prospective Cohort Study in Burkina Faso and Uganda
Source: PLoS One. 2016 Jul 5;11(7):e0156954. doi: 10.1371/journal.pone.0156954 (PMC4933335; doi:10.1371/journal.pone.0156954)
Supplement: S1 File — (DOC) [file pone.0156954.s002.doc]

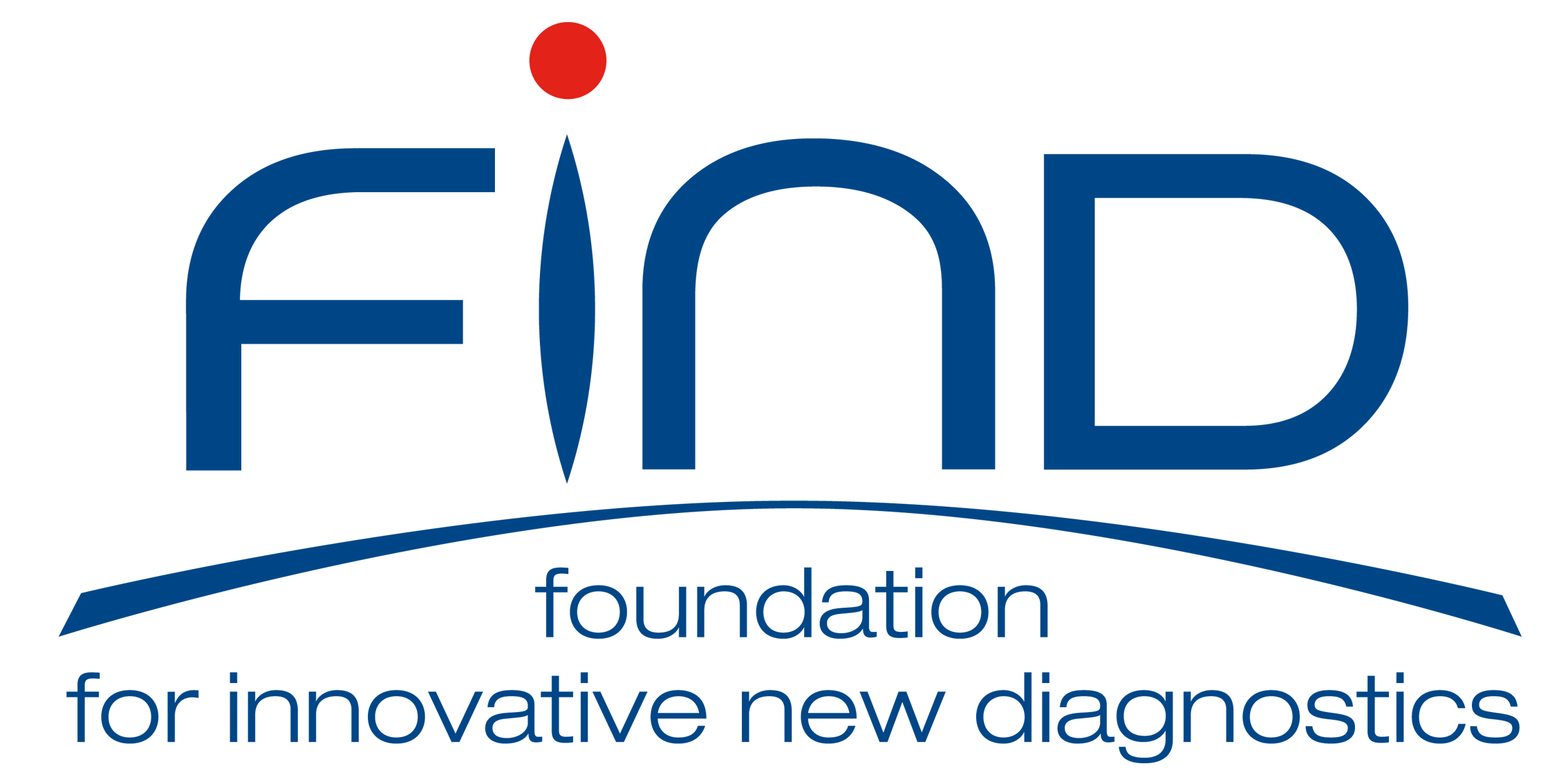


| **STUDY PROTOCOL:**  **Uganda study site** |
| --- |
|  |
| **Malaria rapid diagnostic tests (RDTs)**  **in pregnancy: Detection of placental malaria** |
|  |
| Version and date: 0.9 / 27 Jan 2011 |
| Trial sites: Antenatal clinics in Burkina Faso, Nigeria, Uganda |

| Study Coordinator: Dr Heidi Hopkins  FIND Uganda  email: heidi.hopkins@finddiagnostics.org  tel: +256 772 911236 | |
| --- | --- |
|  |  |
|  | |


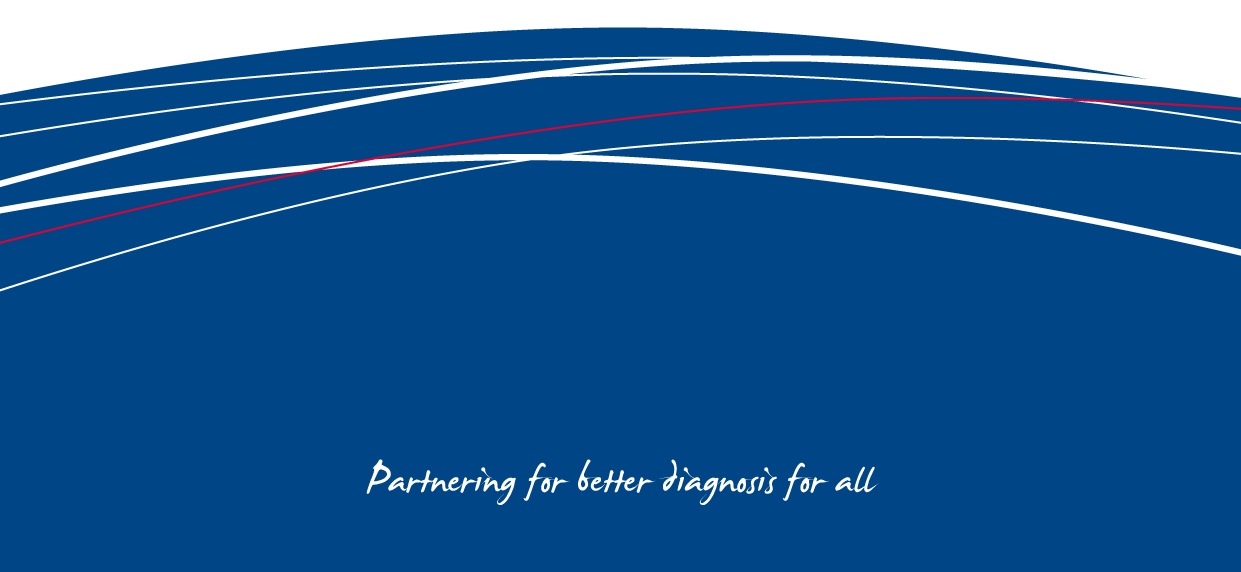

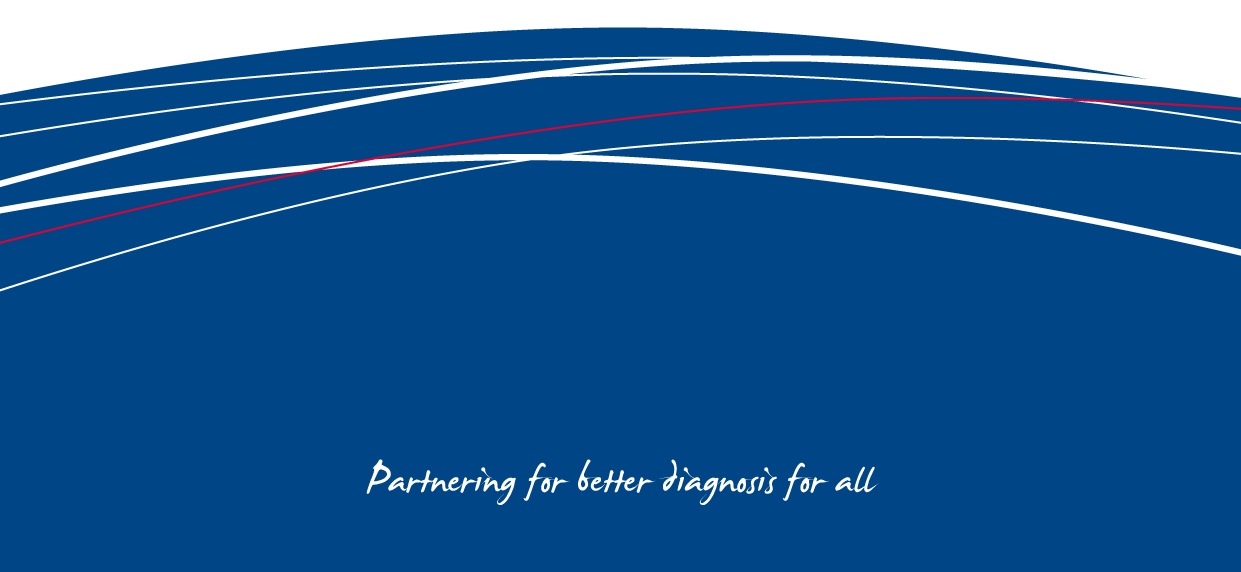


*Technical and Financial Agency:*

**FIND** Geneva tel: +41 22 710 0590 fax: +41 22 710 0599 www.finddiagnostics.org

**CONFIDENTIALITY STATEMENT:**

The information contained in this document, especially unpublished data, is the property of FIND (or under its control) and may not be reproduced, published or disclosed to others without written authorization.

**TABLE OF CONTENTS**

**1 GENERAL INFORMATION** ………………………………………………………………... 4

**2 STUDY OVERVIEW AND PROTOCOL SUMMARY** ……………………………………… 5

**3 BACKGROUND AND RATIONALE** ……………………………………………………….. 6

**3.1 Background Information** ………………………………………………………... 6

3.1.1 Epidemiology and risks of malaria in pregnancy ……………………………… 6

3.1.2 Diagnosis of malaria in pregnancy …………………………………………… 6

3.1.3 Current prevention and control methods for malaria in pregnancy ……….. 7

3.1.4 Potential loss of efficacy of IPTp with SP due to parasite resistance ……… 8

**3.2 Significance and Scientific Rationale** …………………………………………… 9

**4 STUDY OBJECTIVES** ………………...…………………………………………………… 9

**5 STUDY DESIGN** ………………………………………………………………………..……. 10

**Figure 1. Patient flow diagram** ……………………………………………………………. 11

**6 STUDY POPULATION** …………………………………………………………………….. 12

**7 METHODS AND STUDY PROCEDURES** ………………………………………………. 12

**7.1 Study sites and personnel** ……………………………………………………….. 12

7.1.1 Selection of study sites ……………………………………………………….. 12

7.1.2 Personnel conducting protocol activities ……………………………………… 13

**7.2 Study enrollment** ..………………………………………………………….…… 13

7.2.1 Selection for recruitment and enrollment ……………………………………… 13

7.2.2 Provision of written informed consent ……………………………………… 15

**7.3 Study activities and data collection at initial visit** ………………………… 17

7.3.1 Clinical data collection ………………………………………………………. 17

7.3.2 Laboratory sample collection …………………………………………………… 17

7.3.3 Administration of antimalarial medicines ……………………………………… 17

7.3.4 Provision of symptomatic and routine antenatal care ……………………. 18

7.3.5 Scheduling of subsequent visit/s ………………………………………………. 19

**7.4 Study activities at subsequent routine antenatal visits** ……………………. 19

7.4.1 Clinical data collection ……………………………………………………….. 19

7.4.2 Laboratory sample collection …………………………………………………… 19

7.4.3 Administration of antimalarial medicines ……………………………………… 19

7.4.4 Provision of symptomatic and routine antenatal care ……………………. 20

**7.5 Study activities at the time of delivery** ………………………………………….. 20

**7.6 Specific clinical and laboratory assessments** …………………………….. 21

7.6.1 Measurement of hemoglobin (Hb) and assessment of anemia …………… 21

7.6.2 Rapid diagnostic tests (RDTs) ………………………………………………. 21

*7.6.2.1 Quality control and storage of RDTs* …………………………………. 21

*7.6.2.2 RDTs to be evaluated* …………………………………………………… 22

*7.6.2.3 Preparation of RDTs* …………………………………………………… 22

*7.6.2.4 Interpretation and recording of RDT results* ………………………… 22

7.6.3 Microscopy …………………………………………………………………….. 22

*7.6.3.1 Preparation and reading of blood smears* ………………………… 22

*7.6.3.2 Quality control of microscopy results* …………………………………. 23

7.6.4 PCR for detection and speciation of parasitemia ………………………… 23

7.6.5 Estimation of gestational age ……………………………………………… 23

7.6.6 Measurement of infant birth weight …………………………………………. 24

7.6.7 Placental histopathology for detection of evidence of placental malaria ….. 24

7.6.8 Collection of data on other maternal and fetal outcomes ………………... 24

**8 SAFETY CONSIDERATIONS** ……………………………………………………………. 24

**8.1 Potential risks** …………………………………………………………………….. 24

**8.2 Known potential benefits** ……………………………………………………….. 25

**8.3 Data safety and monitoring board** ……………………………………………… 25

**9 FOLLOW-UP** …………………………………………………………………………………. 25

**10 DATA MANAGEMENT** …………………………………………………………………….. 25

**10.1 Data quality assurance and monitoring** ……………………………………… 25

**10.2 Records** ……………………………………………………………………………... 26

**10.3 Data management** ………………………………………………………………... 26

**10.4 Immediate and long-term use of the data** ……………………………………… 26

**11 STATISTICAL CONSIDERATIONS** ……………………………………………………….. 27

**11.1 Study outcome measures** ……………………………………………………….. 27

**11.2 Analysis plan** ………………………………………………………………………… 27

**11.3 Sample size considerations** …………………………………………………… 29

**11.4 Participant enrollment and follow-up** ………………………………………….. 30

**12 QUALITY ASSURANCE** …………………………………………………………………….. 30

**13 EXPECTED OUTCOMES OF THE STUDY** ………………………………………………. 30

**14 DISSEMINATION OF RESULTS AND PUBLICATION POLICY** ……………………. 30

**15 DURATION OF THE PROJECT** ……………………………………………………………. 31

**16 PROBLEMS ANTICIPATED** ………………………………………………………………... 31

**17 ETHICAL CONSIDERATIONS AND PARTICIPANT CONFIDENTIALITY** …………… 31

**REFERENCES** …………………………………………………………………………………. 32

**Appendix A. Informed consent form for study participation**

**Appendix B. Additional consent for stored samples**

**Appendix C. Enrollment form**

**Appendix D. Data collection form, Day 0**

**Appendix E. Data collection form, Subsequent visit**

**Appendix F. Data collection form, Delivery visit**

**1 GENERAL INFORMATION**

**Protocol Title: MALARIA RAPID DIAGNOSTIC TESTS (RDTs) IN PREGNANCY: DETECTION OF PLACENTAL MALARIA**

**Version number and date: 0.9, 27 Jan 2011**

**Investigators:**

1. Heidi Hopkins, MD, MPH

FIND Uganda, Plot 45B/47A Lumumba Ave, PO Box 34663

Kampala, UGANDA

Tel: +256 772 911236

E-mail: [heidi.hopkins@finddiagnostics.org](mailto:heidi.hopkins@finddiagnostics.org)

1. Miriam Nakalembe, MBChB, MMed

Department of Obstetrics & Gynecology, Makerere University Faculty of Medicine

Kampala, UGANDA

Tel: +256 753 857433

Email: ivuds@yahoo.com

1. Jean-Bosco Ouedraogo, MD, PhD

Directeur de Recherche, IRSS, Direction Régionale de l'Ouest

399, Avenue de la Liberté
01 BP 545 Bobo-Dioulasso 01

BURKINA FASO
Tél : +226 20981880  Fax +226 20974868
Email: [jbouedraogo.irss@fasonet.bf](mailto:jbouedraogo.irss@fasonet.bf) or [jbouedraogo@gmail.com](mailto:jbouedraogo@gmail.com)

1. Issaka Zongo, MD

IRSS, Direction Régionale de l'Ouest
(as above)

Email : issaka.zongo@lshtm.ac.uk or zongo_issaka@yahoo.fr

1. David Bell, MBBS, PhD

GMP/WHO

World Health Organization

20, avenue Appia

Geneva, SWITZERLAND

bellda@who.int

Tel: +41 792 691 638

1. Jane Cunningham, MD, MPH

Technical Officer

WHO/IER/TDR/DQR

World Health Organization

20, avenue Appia

1211 Geneva 27, SWITZERLAND

[cunninghamj@who.int](mailto:cunninghamj@who.int)

Tel: +41 (22) 791 2230

1. Wellington Oyibo, PhD

Tropical Disease Research Laboratory, Department of Medical Microbiology and Parasitology

College of Medicine, University of Lagos

Idi-Araba, Lagos , NIGERIA

Tel: +234-8035374004

E-mail: [wellao@yahoo.com](mailto:wellao@yahoo.com)

**Adviser (Uganda site):**

Dr Patrobas Mufubenga

Malaria Control Programme

Uganda Ministry of Health

Kampala, UGANDA

Tel: +256 772 455122

E-mail: pmufubenga2000@yahoo.com

**2 STUDY OVERVIEW AND PROTOCOL SUMMARY**

Study population: Women presenting for routine antenatal care in the second and third trimesters of pregnancy.

Number of Sites: Antenatal clinics at ≥3 sites of varied malaria transmission intensity in Africa; proposed collaborating sites in Burkina Faso, Nigeria, and Uganda. (NB: At present, funds are available to complete the study in two sites, in Burkina Faso and in Uganda; if additional resources permit in the future, the study will also be implemented at the Nigerian site.)

Study Duration: Approximately 12 months from the start of study activities at each site, depending on rates of enrollment.

General objective: To determine whether screening pregnant women for malaria with RDTs may detect placental infection and predict risk of poor birth outcomes due to malaria in areas of varied malaria transmission in Africa.

Specific primary objectives:

1. To compare the results of RDTs, microscopy and PCR performed on peripheral blood from women in the second and third trimesters of pregnancy in malaria-endemic areas of Africa.
2. To determine the positive and negative predictive values of results of RDTs, microscopy and PCR performed on peripheral blood from women in the second and third trimesters of pregnancy, as compared with evidence of placental infection by histological examination after delivery.

Specific secondary objectives:

1. To measure and compare the frequency of pregnancy outcomes (infant birth weights, maternal hemoglobin), prevalence of placental malaria, and risk of symptomatic malaria among women in the study cohort. Participants who test positive by RDT at the time of usual IPTp administration will receive artemisinin-combination treatment (ACT) or quinine; participants who test negative by RDT will receive standard-of-care IPTp with SP.
2. To collect samples for future laboratory analyses related to malaria in pregnancy, including the measurement of the prevalence of molecular markers of antimalarial resistance in the study cohorts.

**3 BACKGROUND AND RATIONALE**

**3.1 Background Information**

*3.1.1 Epidemiology and risks of malaria in pregnancy*

Pregnant women are at particular risk of malaria infection and its consequences. An estimated 50 million women in endemic areas become pregnant each year, and of these approximately half are in sub-Saharan Africa where *Plasmodium falciparum* infections predominate and transmission intensity is significantly higher than in other parts of the world.^1, 2^ Based on review of cross-sectional data, in the absence of preventive efforts it is estimated that approximately one in four pregnant women in areas of stable malaria transmission in Africa have evidence of infection at the time of delivery.^3^ In moderate and high transmission areas, younger women and paucigravidae are typically at higher risk than older women and multigravidae, indicating some protective effect of age- and parity-specific immunity.^4-6^ The risks presented to mother and fetus by malaria infection may be exacerbated by co-existing conditions including malnutrition, other parasitic infections, and HIV infection.^1, 7^

The negative effects of malaria infection in pregnancy have been recognized for decades.^8-11^ The specific effects of malaria in pregnancy depend on host immunity, which depends in turn on malaria transmission intensity.^2^ In areas where malaria transmission is low or unstable, individuals do not acquire immunity with repeated exposure, and pregnant women typically develop symptoms when parasitemic. In these epidemiologic conditions, important risks of malaria in pregnancy include spontaneous abortion, premature delivery, stillbirths, and maternal death from severe malaria. In contrast, in areas where malaria transmission is moderate to high and stable, adults develop partial immunity through repeated exposure, and most malaria infections are asymptomatic. Under these conditions, pregnant women are at risk for anemia (including severe anemia with potential for maternal death)^12^ and placental malaria. Placental malaria infection, while often asymptomatic, has been clearly shown to be associated with low birth weight due to both premature delivery and intrauterine growth retardation; low birth weight in turn is associated with higher rates of infant mortality and poor child development.^1, 2, 13, 14^

*3.1.2 Diagnosis of malaria in pregnancy*

For purposes of epidemiological investigations, the gold standard for diagnosis of malaria in pregnancy is histopathological examination of the placenta after delivery.^15-17^ As recently summarized by Rogerson, et al, microscopic observation of histological changes in the placenta, including the presence of hemozoin deposits, may be used to confirm current and previous malaria infections, and a variety of laboratory protocols have been suggested for preparation of such samples.^16, 18^ A number of studies have documented the low sensitivity of light microscopy of peripheral blood smears for detecting placental infection.^19^ Importantly, some studies have indicated that submicroscopic malaria infections, identified with PCR or antigen-detection tests, may be associated with negative clinical outcomes including maternal anemia^19, 20^ and low birth weight.^21^ However, PCR is not available outside research settings, and antigen-detection tests have not yet been evaluated adequately for use in pregnancy.

Rapid diagnostic tests (RDTs) detect parasite antigen circulating in the blood of infected individuals. Malaria RDTs, which do not require the laboratory infrastructure or expertise of microscopy, are increasingly seen as a reliable alternative for symptomatic case management in virtually all endemic settings. The accuracy of well-made and correctly performed RDTs has been demonstrated in a variety of settings.^22-25^ The WHO currently recommends that parasite-based diagnosis, with either microscopy or rapid diagnostic tests (RDTs), be used for case management in all malaria-endemic areas except in certain circumstances (e.g. severe outbreaks proven to be malaria in resource-poor situations, young children in high transmission settings).^26^ Many malaria control programs in Africa are working to institute national policies of parasite-based diagnosis, and to expand availability of parasite-based diagnosis to more remote areas through the use of RDTs. The quality of commercially produced RDTs is formally evaluated by product testing and lot-testing through the WHO/FIND malaria RDT evaluation program in collaboration with the US Centers for Disease Control and Prevention and a network of other laboratories (<http://www.wpro.who.int/sites/rdt/who_rdt_evaluation/>).^27^ In addition, the WHO/FIND program facilitates lot testing of RDTs procured by national malaria control programs through a network of regional laboratories (<http://www.wpro.who.int/sites/rdt/who_rdt_evaluation/lot_testing.htm>).

We have identified seven publications on six studies that evaluated RDTs for detection of malaria infections in peripheral and/or placental blood at delivery.^19, 28-32^ In general, these studies show that at delivery, the correlation of RDT results (and especially histidine-rich protein 2 [HRP2] RDT results) in peripheral blood with evidence of placental infection falls somewhere between that of microscopy and PCR. However, none of the published studies compared birth outcomes or placental findings with testing performed during gestation (as opposed to at delivery), and none reported results of placental histology. More recently, a study in southwest Uganda monitored pregnant women weekly with microscopy, HRP2-based RDT and PCR, and found an RDT sensitivity of 65% and specificity of 99% when compared with PCR on the same peripheral blood sample.[Mehul Dhorda, personal communication] Another recently-completed study from Ghana compared three treatment groups: one group of pregnant women received IPTp with SP, the second received SP only if the screening RDT was positive, and the third group received artesunate+amodiaquine if the screening RDT was positive. Preliminary results from this study show no statistically significant difference among the groups in severe maternal anemia or risk of low birth weight.[Harry Tagbor, personal communication] In summary, as yet there is no confirmed sensitive and practical approach to diagnosing placental malaria during gestation, at a time that more efficacious preventive measures could be appropriately targeted; but evidence to date on the potential for RDTs to fill this role is promising.

*3.1.3 Current prevention and control methods for malaria in pregnancy*

In light of the heavy burden of malaria in pregnancy, and the lack of practical diagnostic strategies and facilities, the WHO’s strategic framework for prevention and control of malaria in areas of stable transmission – which includes much of sub-Saharan Africa – includes intermittent preventive treatment in pregnancy (IPTp), insecticide-treated bed nets (ITNs), and case management of malaria illness and anemia.^2^ For treatment of symptomatic malaria illness in the second and third trimesters of pregnancy in Africa, the WHO currently recommends artemisinin-based combination treatment (ACT) on the basis of significant evidence of efficacy and safety.^26, 33^ For prevention, the WHO recommends: “In areas of high transmission, IPT with SP should be administered to pregnant women at least twice during the second and third trimesters of pregnancy, and three times in the case of HIV-positive pregnant women. The effectiveness of IPT should be monitored in light of increasing SP resistance.”^34^ By the end of 2006, 33 of the 45 African countries had adopted IPTp as national policy.^34^ The evidence base for benefit of IPTp with SP is broad, supported by a number of studies conducted in the 1990s and early 2000s.^35-39^ Specifically, the intervention has been clearly shown to significantly reduce the prevalence of maternal anemia, the prevalence of placental parasitemia, and the incidence of low birth weight. However, reduced parasite susceptibility to SP appears to threaten the effectiveness of IPTp with this drug.

*3.1.4 Potential loss of efficacy of IPTp with SP due to parasite resistance*

Over the past several years, the spread of resistance to SP as a regimen for treatment of symptomatic malaria infections has been widely recognized. Mutations in the parasite genes for dihydropteroate synthase (DHPS) and dihydrofolate reductase (DHFR) mediate resistance to sulfadoxine and pyrimethamine, respectively, and parasites with multiple mutations in both genes are common throughout East and Southern Africa,^40^ including in study populations of pregnant women,^41^ and appear to correlate negatively with treatment outcomes especially in individuals with less acquired immunity.^42, 43^ A 2007 review paper reported continued benefit of IPTp with SP in areas with high rates of SP treatment failure;^44^ however, a recent study in Tanzania identified a new “triple: triple” mutant parasite and documented associated high failure rates of SP in treatment of symptomatic children and asymptomatic infants,^45^ and data on decreasing effectiveness of IPTp with SP is beginning to emerge.[Stephen Rogerson, unpublished data]

Because of evidence of increasing SP resistance, and potential implications for loss of efficacy of IPTp with SP, as well as evidence for declining malaria transmission rates in many parts of Africa and a consequent need to rethink the risk/benefit ratios of preventive methods, the WHO and others have called for research into new approaches to prevention of malaria in pregnancy.^2, 46-48^

## 3.2 Significance and Scientific Rationale

Malaria prevention measures for pregnant women are critical and available, but the effectiveness of IPTp with SP, a cornerstone in this prevention effort, is declining with increasing parasite resistance. New drugs for IPTp are being considered, but there are disadvantages to presumptive use of the few remaining efficacious antimalarials. An alternative approach may involve screening with diagnostic tests to better target efficacious antimalarial treatment to asymptomatic women with laboratory evidence of malaria infection. Light microscopy of peripheral maternal blood misses a large proportion of cases, and PCR is unavailable in routine health care settings. Preliminary evidence suggests that detection of parasite antigen in peripheral blood may provide an accurate indicator of clinically significant infections and predict pregnancy outcomes. Therefore, screening with RDTs may offer an accurate and practical way to identify pregnant women who will benefit from targeted therapy for placental malaria infection. Antigen detection thresholds vary widely among RDTs, and the distribution of target antigens in peripheral blood circulation is expected to differ; therefore, the potential value of RDTs in this population can best be established by evaluating the detection of placental parasitemia for highly-characterized RDTs, enabling results to be extrapolated to other products and programs. The study described below is proposed to address this question.

1. **STUDY OBJECTIVES**

## 4.1 General objective

To determine whether screening pregnant women for malaria with RDTs may detect placental infection and predict risk of poor birth outcomes due to malaria in areas of varied malaria transmission in Africa.

Specific primary objectives:

1. To compare the results of RDTs, microscopy and PCR performed on peripheral blood from women in the second and third trimesters of pregnancy in malaria-endemic areas of Africa.
2. To determine the positive and negative predictive values of results of RDTs, microscopy and PCR performed on peripheral blood from women in the second and third trimesters of pregnancy, as compared with evidence of placental infection by histological examination after delivery.

Specific secondary objectives:

1. To measure and compare the frequency of pregnancy outcomes (infant birth weights, maternal hemoglobin), prevalence of placental malaria, and risk of symptomatic malaria among women in the study cohort. Participants who test positive by RDT at the time of usual IPTp administration will receive artemisinin-combination treatment (ACT) or quinine; participants who test negative by RDT will receive standard-of-care IPTp with SP.
2. To collect samples for future laboratory analyses related to malaria in pregnancy, including the measurement of the prevalence of molecular markers of antimalarial resistance in the study cohorts.
3. **STUDY DESIGN**

(See Figure 1 on next page.) Women will be enrolled at their initial presentation for antenatal care after quickening, i.e. on the first antenatal visit when they are eligible for IPTp according to WHO recommendations.^2^ On the usual IPTp schedule, typically once in the second and once in the third trimester, blood will be collected from all participants for RDT testing, preparation of thick and thin smears for light microscopy, and PCR. RDTs detecting histidine-rich protein 2 (HRP2) and plasmodium lactate dehydrogenase (pLDH) will be used for testing. All participants with negative RDT results will receive SP, and those with a positive RDT result will receive ACT or quinine (according to national policy for treatment of malaria in pregnancy). All participants will be followed up to delivery. At delivery maternal peripheral and placental blood samples will be collected for RDT testing, light microscopy, and PCR, and placental tissue samples will be processed for histopathological examination. Measurement of maternal hemoglobin and infant birth weight will be obtained.

**Figure 1. Patient flow diagram to evaluate association of RDT results with evidence of placental malaria infection and pregnancy outcomes**

**Pregnant woman presents for routine antenatal care “after quickening” and before onset of labor**

**Study enrollment**

- All selection criteria met
- History & clinical exam
- Venous blood for Hb, RDTs, thick/thin smear, and storage in EDTA, red cell pellet and filter paper for PCR
- Provision of other standard care (ITN, iron + folate supplementation, etc) according to national guidelines

**RDT positive**

**(either HRP2 or pLDH)**

**RDTs negative**

**(both HRP2 and pLDH)**

- **DOT IPTp with SP**
- Management of any symptoms at clinician’s discretion according to national guidelines and standard of care
- **ACT (according to nat’l policy)**
- Management of any symptoms at clinician’s discretion according to national guidelines and standard of care
- **Follow up 2 weeks later for DOT IPTp with SP**

**Subsequent routine antenatal visit/s**

(≥ 1 month after previous visit and before onset of labor)

- History & clinical exam
- Venous blood for Hb, RDTs, thick/thin smear, and storage in EDTA, red cell pellet and filter paper for PCR
- Provision of other standardized care (ITN, iron + folate supplementation, etc) according to national guidelines

**RDT positive**

**(either HRP2 or pLDH)**

**RDTs negative**

**(both HRP2 and pLDH)**

- **DOT IPTp with SP**
- Management of any symptoms at clinician’s discretion according to national guidelines and standard of care
- **ACT (according to nat’l policy)**
- Management of any symptoms at clinician’s discretion according to national guidelines and standard of care
- **Follow up 2 weeks later for DOT IPTp with SP**

**Follow up at delivery**

- Maternal peripheral blood for Hb, RDTs, smear, and PCR
- Placental blood for RDTs, smear, and PCR
- Placental tissue sample to process for histopathological examination
- Measurement of infant birth weight
- (Other data on any maternal or fetal morbidity/mortality)

1. **STUDY POPULATION**

Participants in this evaluation will include pregnant women presenting for routine antenatal services in areas of Africa where IPTp is considered standard of care according to WHO and national malaria control program guidelines. Pregnant women of any parity will be eligible for enrollment. Anticipated sites include antenatal clinics in Burkina Faso, Nigeria, and Uganda. Specific participant selection criteria will include (please see also section 7.2 on participant enrollment):

1. Presenting for care after quickening and before onset of labor (i.e. in the second or third trimester of pregnancy)
2. Age between 16 years and 44 years, inclusive
3. Willingness and ability to follow up with study visits and activities through the duration of pregnancy and at delivery
4. Absence of history of serious adverse reaction to sulfa drugs
5. Absence of history of serious adverse reaction to artemisinin-based drugs or quinine (depending on national policy on treatment of malaria in pregnancy)
6. Absence of HIV infection (both because guidelines for malaria prevention in pregnancy for HIV-infected women differ from those for HIV-negative women, and in order to avoid confounding of pregnancy outcomes by HIV-related complications or treatments in this early evaluation)
7. Absence of history of or current obstetrical complications (e.g. pre-eclampsia, eclampsia, hypertension during pregnancy, post-partum hemorrhage, evidence of multiple gestation)
8. Absence of chronic disease (e.g. diabetes mellitus, sickle cell disease)
9. Absence of evidence of severe acute disease requiring inpatient management or referral
10. Provision of written informed consent
11. Enrollment Hb ≥7 g/dL

**7 METHODS AND STUDY PROCEDURES**

**7.1 Study sites and personnel**

*7.1.1 Selection of study sites*

Study sites in each country will be health centers, either government-run or private/missionary, that provide routine antenatal and peri-natal care. The health centers for this evaluation will be selected on the basis of the following characteristics: representation from a range of malaria transmission and geographic zones within Africa; interest and capacity of local researchers and public health authorities; accessibility from the collaborating research team’s base; interest and capacity of the health center staff; and patient numbers adequate to allow enrollment of the target sample size within a period of approximately 6 months. On the basis of current information and preliminary agreements, selected sites include: Colsama government health center in District de Dô, a peri-urban area near Bobo-Dioulasso, Burkina Faso (seasonal transmission); St Kizito Catholic Hospital and/or Regina Mundi Catholic Hospital, Lagos, Nigeria (low year-round transmission); and Tororo District Hospital in eastern Uganda (high year-round transmission). If for unforeseen reasons we are unable to conduct the evaluation at the specific centers listed, alternative sites will be chosen to preserve the representative range of geography and malaria endemicity.

*7.1.2 Personnel conducting protocol activities*

For the purposes of this study, routine antenatal and delivery care will be provided by the staff of participating health centers, i.e. health workers who are employed by the health center (all participating health centers are either government- or missionary-sponsored established health facilities). Such health workers typically include midwives, nurses, and other similar cadres of health care staff. In this protocol, these personnel are referred to as “health center staff.”

Study-specific activities will be conducted by personnel hired for the purposes of the research study, i.e. individuals employed by the study coordinators. Throughout this protocol, this personnel group is referred to as “study staff.” Study staff will typically be individuals with laboratory and/or clinical background and with prior experience in clinical malaria research. Both health center staff and study staff will be trained in the protocol and relevant study procedures prior to the start of the study, and at each site the personnel groups will work together to achieve the study goals while ensuring smooth continuation of care. Further details for each site will be described in the site-specific protocols.

**7.2 Study enrollment**

*7.2.1 Selection for recruitment and enrollment*

At each site, pregnant women will be invited to enroll in the study when they present to the participating health center for routine antenatal care. At each site, potential participants will be referred by health center staff to study staff, and will be screened and enrolled by study staff according to the selection criteria listed in section 6. At each participating site, information on current health status, obstetric history, and chronic conditions including HIV infection (see selection criteria 6-9 in section 6 above) are collected by health center staff as part of routine antenatal care. HIV counseling and testing is routinely offered to all pregnant women on an opt-out basis at each site. Study staff will conduct screening interviews with each potential participant after she has completed her antenatal visit for that day. Study staff will rely on health center staff’s diagnosis of these conditions in determining whether a potential participant meets all selection criteria. In case of questions about whether a participant meets all criteria on medical grounds (e.g. presence or absence of chronic disease), study staff will confer with health center staff, and where appropriate with the potential participant, to reach a final decision on whether to offer enrollment.

***Uganda site-specific information (as requested by WHO; italic font):*** *At the Uganda study site (Tororo District Hospital, TDH), women presenting for routine antenatal care are first welcomed at the reception desk of the ANC in the hospital compound. At the reception desk, each attendee is registered in the ANC book, including general information such as name, age, pregnancy status, and residence. If an attendee is pregnant and aged 16 to 44 years (inclusive), the health center reception staff will notify her that she may be eligible for this study, and ask if she would like to learn more about the study. If she agrees, she will be invited to talk in a private area of the ANC with a member of the study staff responsible for obtaining informed consent. If she declines to learn more about the study, or declines participation after the consent discussion, she will have no further contact with study staff, and will continue her antenatal visit as usual with health center staff. If she agrees to participate, she will sign or fingerprint the informed consent form (see section 7.2.2) and will continue with her antenatal visit as usual and including the study activities described in section 7.3. Newly enrolled study participants will undergo the usual routine antenatal screening and care provided at TDH (see further details below).*

***(End Uganda site-specific information.)***

Those women who are screened and found to be ineligible for the study on medical grounds (i.e. selection criteria 6-9 and 11) will be managed according to routine practice by health center staff. At each site, HIV counseling and testing is linked to an established treatment program to reduce mother-to-child HIV transmission. Other acute or chronic conditions are managed on-site as capacity allows, or referred to a higher level of the health care system according to local guidelines.

***Uganda site-specific information (as requested by WHO; italic font):*** *As a matter of routine, information pertaining to the age, gestational status, HIV status, and medical and obstetric history is obtained by health center staff during typical antenatal visits at TDH. In particular, as a matter of routine and Uganda national policy, all antenatal attendees are invited to undergo HIV testing. If a potential participant declines routine HIV testing, she will no longer be eligible for the study; she will be so informed, she will not participate in any further study activities, and she will continue with the usual care available at TDH. If a participant agrees to the routine HIV testing and tests negative, she will continue with routine care at TDH and with study activities as described below. If a participant agrees to the routine HIV testing and tests positive, she will no longer be eligible for the study; she will be so informed, she will not participate in any further study activities, and she will be referred to local HIV health care providers for appropriate care. HIV care at the TDH site is typically provided through The Aids Support Organization (TASO), a well-established service which provides counseling, advice and materials for prevention of mother-to-child transmission, prophylaxis of opportunistic infections according to national guidelines, antiretroviral therapy according to national guidelines, and other appropriate services.*

*In addition to HIV, other medical conditions or history may render a potential participant ineligible for the study, including history of adverse reactions to antimalarial drugs, history of obstetrical complications, history or presence of chronic disease, etc (see selection criteria Section 6 and Appendix C Enrollment form). For women who have signed the informed consent form, after completion of the antenatal visit, study staff will confer with the woman and the health center clinician to confirm whether the woman meets all selection criteria. If so, a study staff member with laboratory training will check the participant’s hemoglobin level as in Sections 7.3.2 and 7.6.1. If all selection criteria are met, the participant will be considered fully enrolled and will continue with study activities as described in Section 7.3. If any selection criterion is not met, the participant will no longer be eligible for the study; she will be so informed, she will not participate in any further study activities, and she will continue with the usual care available at TDH.*

***(End Uganda site-specific information.)***

*7.2.2 Provision of written informed consent*

Each participant will provide written informed consent (Appendices A and B) at the time of enrollment. A trained study staff member will conduct individual screening interviews and informed consent discussions with potential study participants. Informed consent will be conducted in the potential participant’s preferred language, with the assistance of a translator if necessary. Consent forms, approved by all relevant ethical review boards, will be available in English (Nigeria, Uganda) or French (Burkina Faso) and in the local language/s for each site. If the participant is unable to read or write, her fingerprint will be substituted for a signature, and a signature from a witness to the informed consent procedures will be obtained. Potential participants will have the opportunity to ask questions and discuss details of the study with study staff, and are free to ask further questions at any time during or after completion of the study activities. In the case of participants under the age of 18, international^49^ and country-specific guidelines on the inclusion of minors in research (including guidelines related to emancipated minors, and/or co-consent from a parent or guardian) will be strictly followed to allow participation by individuals in this vulnerable group, and therefore to allow the future application of data and potential benefits from this study to the same.

***Uganda site-specific information (as requested by WHO; italic font):*** *Ugandan guidelines on parental consent for pregnant minors, and related risks, are as follows. According to the National Guidelines for Research Involving Humans as Research Participants of the Uganda National Council for Science & Technology (UNCST), published March 2007, section 9.3 on Mature and Emancipated Minors states: “Mature minors are individuals 14-17 years of age who have drug or alcohol dependency or a sexually transmitted infection; while emancipated minors are individuals below the age of majority who are pregnant, married, have a child or cater for their own livelihood. Mature and emancipated minors may independently provide informed consent to participate in research if: a. In the view of the IRC the research is not objectionable to parents or guardians (established by the IRC with evidence from the community); b. The research protocol includes clear justification for targeting mature and emancipated minors as participants; and a clear justification for not involving parents or guardians in the consent process.”*

*For the purposes of this study, and as discussed with UNCST personnel, this study will take the following approach: For biological, scientific and ethical reasons, pregnant women/girls aged 16-44 years (who meet all selection criteria) will be eligible for this study.  To be useful, the study results should be applicable to the general population of pregnant women/girls in malaria-endemic areas; as pregnancy before age 18 is not uncommon in these areas, the study should include participants from the younger age range, in order to provide representative results.  In addition, participants may (or may not) benefit from study participation, and that potential benefit should be extended to typical primigravidae as well as older women. Pregnant participants aged 16-17 are minors, and may be considered “mature and emancipated minors” according to the UNCST guideline. For informed consent purposes, we will therefore consider two categories for 16-17 year-olds who wish to participate in the study:*

*a) A prospective minor participant comes to the ANC with her parent, or with a guardian figure (older sibling, husband, etc).  In this case we will seek both written assent from the minor, and written consent from the adult parent/guardian.*

*b) A prospective minor participant comes to the ANC alone, and wishes to consent for her own participation.  In this case, we will seek only her written consent, as she is considered a mature and emancipated minor.*

***(End Uganda site-specific information.)***

If the participant meets all study enrollment criteria, she will be enrolled (Appendix C).

**7.3 Study activities and data collection at initial visit**

*7.3.1 Clinical data collection*

At the time of enrollment, information will be collected from each participant on her medical and pregnancy history, and a brief clinical assessment will be performed by a health center staff clinician (see sample data collection form, Appendix D).

*7.3.2 Laboratory sample collection*

Study staff will collect blood from participants for use in study laboratory procedures, and will prepare the samples and conduct the diagnostic tests described; all other care will be provided by health center staff (i.e. clinicians, typically midwives and nurses, employed by the health center). Each participant will provide a 2 mL (two milliliter) venous blood sample for HemoCue hemoglobin (Hb) measurement, preparation of two RDTs, preparation of thick and thin smears for expert light microscopy, and storage in a microtainer and on filter paper for PCR (see further details in section 7.6 below). Hb and RDT results will be used in clinical management as in section 7.3.3 and 7.6.1 below; expert microscopy and PCR results will not be immediately available and will not be used in management.

*7.3.3 Administration of antimalarial medicines*

If both RDT results are negative, the participant will receive a complete dose of SP by directly observed therapy (DOT) on the day of enrollment. If one or both RDTs are positive, she will receive a complete curative dose of ACT or quinine according to the national policy for treatment of parasitemia in pregnancy; the initial dose will be given by DOT, and the remaining doses will be given to the participant with clear instructions for completing the regimen at home. SP or ACT/quinine will be administered by health center clinical staff. If a patient receives curative treatment with ACT or quinine a return visit (or home visit by study staff) to administer a dose of SP will be scheduled for 2 weeks after the end of the curative treatment. The rationale for use of both curative treatment and SP-IPTp for RDT-positive women is as follows: The mode of action of IPTp is not yet completely understood, but is presumed to be two-fold: an IPTp dose *treats* existing asymptomatic infections, and also *prophylaxes* against new infections due to the long half-life of the drug used (e.g. SP). There is some evidence that the primary benefit may result from “intermittent suppressive chemoprophylaxis.”^50^ Quinine does not have such a prophylactic effect, and while ACTs pair a short-acting artemisinin compound with a longer-acting drug, there is as yet no evidence that ACTs provide a prophylactic effect similar to SP in pregnancy. Therefore, we believe that the safest approach for the current study is to provide both curative treatment, and the currently recommended IPTp drug, i.e. SP. While there are no published data that raise concern for clinically significant drug interactions among these antimalarials, for reasons of tolerability and due to the limited data on pharmacokinetics in pregnant populations we will administer the drugs 2 weeks apart.

*7.3.4 Provision of symptomatic and routine antenatal care*

(See also Uganda site-specific information in section 7.2.) Any symptoms reported by the participant at the time of the initial visit will be investigated and managed according to the local standard of care by the health center staff clinician providing care. Data will be collected on symptoms reported and any treatments given. Each participant will be offered a standard antenatal care package, according to WHO and national guidelines, including iron and folate supplementation, nutritional advice, and a long-lasting insecticide-treated bed net (LLIN); information on provision and use of such care will be recorded at enrollment and in subsequent visits. Each participating health center is affiliated with a network of government- or missionary-sponsored clinics/hospitals providing care free or at reduced cost. The standard of care for any medical problem that exceeds the capacity of the participating center is to refer to a higher level of care. As clearly described during the informed consent discussion, symptom management and referral will be done at the discretion of the health center staff; study staff will not be able to assist financially. Please see paragraph below for Uganda site-specific details on this topic.

***Uganda site-specific information (as requested by WHO; italic font):*** *The usual standard of care for the care and follow-up of pregnant women at TDH follows national guidelines from the Uganda Ministry of Health. Pregnant women are provided, for free according to resource availability, with a standard antenatal care package, including access to the four recommended antenatal visits for basic assessment, symptomatic management (empiric treatment of urinary tract infections and sexually transmitted diseases, analgesics for pain syndromes, and similar), hemoglobin estimation where available, iron and folate supplementation, antihelminth treatment, routine counseling and testing for syphilis and HIV, a long-lasting insecticide-treated bed net (LLIN), and advice and counseling on nutrition and on prevention of sexually transmitted diseases. The standard of care for any medical problem that exceeds the capacity of the participating center is to refer to a higher level of care. Participants in this study will be assured access to components of the basic antenatal package including routine assessment by health center staff, iron and folate supplementation, and a LLIN. As clearly described during the informed consent discussion, symptom management and referral will be done at the discretion of the health center staff. As discussed with the Uganda ethical review committee (Makerere University Faculty of Medicine Research and Ethics Committee), study staff including a clinician may assist with providing standard care to study participants in case of need and in collaboration with health center staff.*

***(End Uganda site-specific information.)***

*7.3.5 Scheduling of subsequent visit/s*

The participant will be encouraged to return to the study site for her subsequent routine antenatal visit/s, and if feasible, for any intervening care-seeking. Subsequent antenatal visits will be scheduled according to the national standard of care, typically including at least one visit in the second trimester and one visit in the third trimester. Routine visits will be scheduled at least 4 weeks apart; participants will not receive more than one dose of SP within any 4-week period. Each participant will be given a card/booklet with dates of future study visits, and in which to record any care or treatment that is received from non-study site providers between routine visits.

**7.4 Study activities at subsequent routine antenatal visits**

*7.4.1 Clinical data collection*

At each subsequent scheduled antenatal visit, information will be collected from the participant on any treatments sought since the previous visit and on current symptoms, and a brief clinical assessment will be performed by a health center staff clinician.

*7.4.2 Laboratory sample collection*

Study staff will collect blood from participants for use in study laboratory procedures, and will prepare the samples and conduct the diagnostic tests described; all other care will be provided by health center staff (i.e. clinicians, typically midwives and nurses, employed by the health center). At each scheduled visit, the participant will provide a 2 mL (two milliliter) venous blood sample for HemoCue Hb measurement, preparation of two RDTs, preparation of thick and thin smears for light microscopy, and storage in a microtainer and on filter paper for PCR (see further details in section 7.6 below). Hb and RDT results will be used in clinical management as in sections 7.4.3 and 7.6.1 below; microscopy and PCR results will not be immediately available and will not be used in management.

*7.4.3 Administration of antimalarial medicines*

If both RDT results are negative, the participant will receive a complete dose of SP by DOT. If one or both RDTs are positive, she will receive a complete curative dose of ACT or quinine (as in section 7.3.3 above); the initial dose will be given by DOT, and the remaining doses will be given to the participant with clear instructions for completing the regimen at home. SP or ACT/quinine will be administered by health center clinical staff. If a patient receives curative treatment with ACT or quinine, a return visit (or home visit by study staff) to administer a dose of SP will be scheduled for 2 weeks after the end of the curative treatment. (See section 7.3.3 for explanation of rationale.)

*7.4.4 Provision of symptomatic and routine antenatal care*

Any symptoms reported by the participant at the visit will be investigated and managed according to the local standard of care by the health center staff clinician providing care, as described in section 7.3.4. Data will be collected on symptoms reported and any treatments given, and information on provision and use of routine antenatal care will be recorded. If, in the judgment of the health center staff, a participant requires referral for medical reasons, this will be done according to usual health center procedures as in section 7.3.4.

**7.5 Study activities at the time of delivery**

At delivery, study staff will collect a 2 mL (two milliliter) blood sample from the participating mother by venipuncture, and from the placenta by standard methods. Maternal peripheral blood will be measured for Hb by Hemocue; both maternal and placental blood will be used to prepare RDTs and blood smears, and for storage in microtainers and on filter paper for PCR (see further details in section 7.6). In addition, placental tissue samples will be processed for histopathological examination using standardized methods. Infant birth weight will be obtained using a calibrated scale. If a participant does not deliver at the study site, study staff will attempt to visit her to obtain data and samples within 24 hours of the birth. Data on date, time and type of birth, any complications, maternal Hb and infant weight, and the date and time of collection of blood and placental samples and outcome data will be recorded.

Testing of mothers for malaria at delivery is not routine, and there is not yet an established standard of care for management of babies born to women testing positive under these circumstances. Congenital malaria remains a poorly understood occurrence.^51, 52^ However, some evidence suggests that risk of congenital malaria is higher for infants of HIV-positive women^53^ (note that this study will not enroll HIV-positive women), and for infants of primigravidae.^54^ Notably, an association between placental infection and clinical malaria in the infant is not yet well established.^54^ For this study, if maternal peripheral blood or placental blood is positive at delivery by RDT, study staff will inform the managing clinician and the mother of the result, and will recommend that the infant be observed for symptoms; if symptoms occur, the infant should be tested (if possible according to local standards of care) and treated for malaria according to national guidelines. In addition, when microscopy results become available, if maternal peripheral or placental blood smears are positive, study staff will notify the mother by phone or home visit. As on previous study visits, if in the judgment of health center clinical staff a participating mother or her infant requires referral for medical reasons, this will be done according to usual health center procedures as in section 7.3.4.

It is recognized that poor birth outcomes may lead to negative and stressful emotions for the woman, those close to her, and her health care providers. In the unfortunate event of a negative birth outcome, study staff will attempt to collect relevant data in a manner that is sensitive, respectful of the woman’s dignity, context-appropriate, and that presents the least inconvenience possible to the woman and her family. Discussion of these ideas, potential scenarios and appropriate methods will be held with study staff and health center staff during pre-study training, and will be based on existing guidance to the extent available.^55, 56^

**7.6 Specific clinical and laboratory assessments**

*7.6.1 Measurement of hemoglobin (Hb) and assessment of anemia*

At the time of each routine antenatal visit, and again at delivery, maternal blood will be used to measure hemoglobin with a HemoCue machine (Quest Diagnostics, Ängelholm, Sweden). Based on WHO definitions, a participant with Hb < 11 g/dL will be considered to be anemic, and Hb < 7 g/dL will be considered to be severe anemia. Anemia will be managed by health center clinical staff according to national guidelines for anemia in pregnancy, which may include additional iron and/or folate supplementation, and/or administration of an anti-helminthic. Data on provision of such care will be recorded (as in section 7.4 above).

*7.6.2 Rapid diagnostic tests (RDTs)*

This study will evaluate the three most commonly used antigen-detection systems in RDTs: *Plasmodium falciparum-*specific plasmodium lactate dehydrogenase (Pf-pLDH), pan-specific plasmodium lactate dehydrogenase (pan-pLDH), and histidine-rich protein 2 (HRP2). According to current test format availability, in order to obtain reliable results for HRP2, pan-pLDH and Pf-pLDH or this study, two different combination RDTs (i.e. each with two test lines detecting different antigens) will be used. RDTs will be obtained directly from their manufacturers, and will be centrally procured. For each of the two types of RDT to be evaluated, all test kits used in the study will be from a single lot. Batch numbers, expiry dates, date received, and date opened will be recorded for each package of RDTs. All study personnel will receive hands-on training in preparation and interpretation of the RDTs before the study begins.

*7.6.2.1 Quality control and storage of RDTs*

Each lot of RDTs will undergo lot testing according to WHO guidelines^57^ before and at the end of the study. RDT testing will take place in the laboratory of Dr John Barnwell at the Centers for Disease Control and Prevention (CDC), Atlanta, Georgia, USA. Prior to the start of the study activities at each site, the RDTs to be evaluated will be stored in their original packaging at room temperature in a central storage space. Stocks of RDTs, adequate to complete training and the study at each site, will be transported to each health center prior to the training and pilot period at each site. Manufacturers recommend that RDTs be stored between 4 and 40° C. Temperature and humidity of the storage areas will not be controlled, but will be monitored and recorded regularly to document storage conditions at each site.

*7.6.2.2 RDTs to be evaluated*

The following criteria will be considered in selecting the RDTs for this evaluation: high performance in WHO/FIND standardized product testing,^27^ ease of use, safety (minimal exposure to blood during test preparation), completeness of packaging, appropriate packaging for transport and storage in tropical environments (each test individually wrapped in foil with plastic liner), reasonable market price, and reliability of the supply of the RDTs.

*7.6.2.3 Preparation of RDTs*

RDTs will be performed on maternal blood obtained by venipuncture at the time of each routine antenatal visit, and at the time of delivery. In addition, RDTs will be performed on placental blood obtained at delivery. Individual RDTs will be labeled with the participant’s unique study number and initials. A member of the study staff will prepare the RDTs according to manufacturer instructions. All personnel will use universal precautions when handling blood samples.

*7.6.2.4 Interpretation and recording of RDT results*

Each RDT will be interpreted by study personnel who are not responsible for clinical management decisions. At the end of the manufacturer-recommended development time for each test (typically 15 minutes), the RDT result will be recorded on the patient’s data form and will be given to the clinician caring for the participant. An RDT result will be considered positive if both the control line and the test line are visible after the development time. A result will be considered negative if the control line is visible, but no test line appears. The result will be considered invalid if no control line is visible, regardless of whether or not a test line appears. If an RDT result is invalid, the test will be repeated using the same sample of venous blood.

*7.6.3 Microscopy*

*7.6.3.1 Preparation and reading of blood smears*

At the time blood is obtained for preparation of RDTs, at each routine antenatal visit and at delivery, a thick and thin blood smear also will be prepared. In addition, at the time of delivery, an impression smear will be prepared from placental blood according to standardized methods. Blood smears will be stained with Giemsa, fixed, and transported to a central laboratory at each participating site for expert reading. Microscopists reading blood smears will be blinded to the RDT results. Thick blood smears will be evaluated for the presence of parasitemia (asexual forms) and gametocytes. Parasite densities are calculated by counting the number of asexual parasites per 200 leukocytes (or per 500, if the count is <10 asexual parasites/200 leukocytes), assuming a leukocyte count of 8,000/μl. A blood smear will be considered negative when the examination of 100 high power fields does not reveal asexual parasites or gametocytes. If a thick smear is positive, the corresponding thin blood smear will be evaluated for parasite species.

*7.6.3.2 Quality control of microscopy results*

Before the commencement of field work, designated microscopists at each site will be pre-qualified through blinded readings on slides prepared according to the WHO slide bank protocol, and required to reach level 1 or 2 expertise level (as described in the WHO Malaria Microscopy Quality Assurance Manual, version 1, Chapter 5).^58^ All slides collected during the study at each site will be read by two microscopists. Slides with discrepant results will be re-read by a third external reader (Level 1 WHO microscopist) and returned to the study site after reading. In addition, 10% of slides from each site will be sent for review at an internationally recognized center of excellence for malaria microscopy. All microscopists will be blinded to RDT results and to results of previous microscopy.

*7.6.4 PCR for detection and speciation of parasitemia*

As for RDT and blood smear preparation, at the time of each routine antenatal visit and at delivery, two drops of maternal blood will be collected onto filter paper and 250uL into a microtainer containing EDTA anticoagulant. In addition, at the time of delivery, placental blood will be collected onto filter paper and into EDTA microtainer/s. Each filter paper sample will be labeled with the participant’s study number, air-dried, and stored in a sealed plastic bag at ambient temperature. Microtainers will be labeled with the participant’s study number and stored at -20°C. Samples will be transferred to a central laboratory and analyzed by PCR to confirm presence or absence of parasitemia, and parasite species. PCR will be performed at the laboratory of Prof Jean-Bosco Ouedraogo in Bobo-Dioulasso, Burkina Faso, and quality assurance of results will be conducted at the laboratory of Dr John Barnwell at the Centers for Disease Control and Prevention, Atlanta, Georgia, USA.

*7.6.5 Estimation of gestational age*

At enrollment, duration of gestation and expected delivery date will be estimated by reported last menstrual period (LMP) and by measurement of fundal height, and these data will be recorded on the participant’s case record forms. At subsequent study visits, fundal height will be measured and recorded.

*7.6.6 Measurement of infant birth weight*

Within 24 hours of delivery, study staff will weigh the infant on a calibrated scale. Birth weight will be recorded in grams; based on WHO definitions, low birth weight (LBW) will be considered < 2500 g.

*7.6.7 Placental histopathology for detection of evidence of prior or current placental malaria*

At the time of delivery, placental blood and tissue sample/s will be collected and processed according to standard methods for smears, storage for molecular analysis, and for histopathological examination. Specific details of placental collection will be described in more detail in site-specific protocols; in general, for births taking place at the health center, health center staff will assist with delivery, including delivery of the placenta, and will then work with study staff to ensure the placenta is transferred to the laboratory area for processing as efficiently as possible to avoid degradation of the samples. As in section 7.5, if a participant does not deliver at the study site, study staff will attempt to visit her to obtain data and samples within 24 hours of the birth. The placenta will be wiped and washed with approximately one liter of phosphate buffered solution (PBS) to remove blood clots, placed umbilical cord side down on a clean, dry cloth, and gently blotted with a paper towel. Placental blood samples and biopsies will be collected and stored for further staining and processing. Placental histology samples will be read by two expert microscopists with discrepant results resolved by a third reader, with a proportion of samples sent to an independent research group for external quality control.

*7.6.8 Collection of data on other maternal and fetal outcomes*

In addition to the specific assessments described above, data on other significant maternal and fetal outcomes will be collected. In particular, the study staff will endeavor to collect data on management of any febrile or afebrile illnesses during pregnancy, and on birth outcomes including requirement and rationale for Cesarean section, any obstetrical complications, and fetal or maternal death. If a participant does not return to the health center at the anticipated time of delivery, study staff will contact the participant by phone and/or conduct a home visit to attempt follow-up. A participant will be considered lost to follow-up if no data can be collected within 7 days of delivery; within the 7-day limit, complete or partial data (e.g. maternal blood and infant weight, but no placenta) may be collected.

**8 SAFETY CONSIDERATIONS**

## 8.1 Potential risks

Risks associated with participation in this evaluation are minimal, essentially no greater than they would be for routine antenatal care in malaria-endemic areas. All participants will receive standard-of-care antenatal services, including provision of IPTp with SP according to WHO and national recommendations. To the routine evaluation of pregnant women at antenatal visits, this study adds the collection of 2 mL (two milliliters) of venous blood, a negligible medical risk. Physical discomfort, transient bleeding and bruising may result when blood is obtained by venipuncture. Aseptic technique and universal precautions against body fluid exposures will be practiced in obtaining blood samples. Some participants will present to the health facility with asymptomatic or symptomatic malaria infection, which is potentially detrimental to both mother and fetus; however, all participants will be managed by qualified clinical staff according to WHO and national standards of care. While loss of privacy is a potential risk associated with participation in any research project, no sensitive information will be collected, and no social risks are foreseen for participating women. Potential for loss of confidentiality will be minimized by use of coded study numbers on data collection forms and laboratory samples, rather than participant names, and all data will be stored in locked filing cabinets and password-protected computer files.

## 8.2 Known potential benefits

All participating women will be provided with basic antenatal services according to the local standard of care. Participants may also benefit from the knowledge that they are assisting in the evaluation of potentially valuable methods for prevention and control of malaria for pregnant women in endemic areas.

## 8.3 Data safety and monitoring board (DSMB)

To ensure the quality and integrity of data, and the safety of participants, an independent study monitor will be contracted for the duration of the study. The monitoring group will be identified and engaged following standard WHO/TDR procedure.

**9 FOLLOW-UP**

No formal study follow-up is planned for participants after delivery, as described in 7.5.

**10 DATA MANAGEMENT**

**10.1 Data quality assurance and monitoring**

Study staff and relevant health center staff will be educated in the study protocol prior to the start of study activities at each site. Data collection forms will be reviewed by the Study Coordinators and Principal Investigators (PIs) for completeness and accuracy. PIs from the different countries will maintain regular contact to ensure consistency in protocol implementation at each site, and PIs will meet regularly with study staff at their site/s to ensure consistency in the collection of data. PIs will participate in regular study group meetings to assess progress of the study, address any difficulties with the RDTs or protocol, and provide feedback to members of the study group. A two-week pilot period will be conducted before beginning the study at each site, which will allow the PIs and study personnel to identify and resolve potential logistical and technological problems prior to beginning data collection. Standardized protocols and SOPs will be followed for quality control/quality assurance of specimen procurement and preparation, smear microscopy, placental histology, and PCR.

**10.2 Records**

All participants will be identified by a unique identifying number throughout the study; participant names will not be recorded or entered into the computerized databases. Data collection forms will be kept in secure files at each study site. All corrections to handwritten information on data collection forms and other study documents will be made by striking through the incorrect entry with a single line and entering the correct information adjacent to it; the correction will be initialed and dated by the study staff member. For any requested information that is not obtained, an explanation will be noted on the data collection form. Electronic databases will be stored in password-protected computer files. Access to the records will be limited to study staff. The investigators and study staff will allow all requested monitoring visits, audits or reviews by relevant ethical review boards.

**10.3 Data management**

Data will be transferred from the handwritten data collection forms into a computerized database by study personnel and will be double-entered to verify accuracy. Back-up files of the database will be created after each data entry session. For quality control, check programs will be written into the databases to limit the entry of incorrect data and ensure entry of data into required fields. Electronic data records will be maintained at each site and will be transmitted to a centralized database for regular cleaning and management.

**10.4 Immediate and long-term use of the data**

Data collected in this study will be compiled, analyzed and made available to collaborating partners, ministries of health in participating countries, the WHO, and will be prepared for publication in a peer-reviewed journal. Study staff and health center staff will be informed of overall study results after data analysis has been completed. Upon completion of the study, all study documents and record forms will be filed and stored at the FIND office in Kampala or Geneva for at least 12 years. Electronic data records will be stored indefinitely by investigator/s in personal computers and external hard drives. Results of the study may be publicized in the future by collaborating partners and stakeholders as part of public health education efforts and malaria control programs.

**11 STATISTICAL CONSIDERATIONS**

Data analysis will be performed by the PIs using Stata statistical software package, with assistance from Michelle Gatton, PhD, of the Queensland Institute of Medical Research and other co-investigators as appropriate.

**11.1 Study outcome measures**

Results of peripheral blood tests performed during gestation will be analyzed for associations with evidence of placental malaria and pregnancy outcomes. The statistical analysis will focus on three primary outcomes: 1) negative predictive power of malaria RDTs, peripheral blood films and PCR performed on peripheral blood to diagnose placental malaria during gestation, 2) association of placental malaria during gestation on mean infant birth weight and 3) association of placental malaria during gestation on mean maternal Hb. These outcomes will be used to assess the potential utility of RDTs as screening tests to identify clinically significant malaria infections at a time when effective preventive measures may be taken. Other factors known to influence the study outcomes, including iron and folate supplementation, bed net use and administration of antihelminthic drugs will be treated as confounders in the analysis. In addition, stratified analysis will be conducted to assess these associations within subgroups based on geographic location, estimates of malaria endemicity, participant age, and parity (gravida class).

**11.2 Analysis plan**

The unit of analysis will be each pregnancy. For each pregnant woman a complete data set consists of the following:

| Variable | Type of data | Variable classification | | | | |
| --- | --- | --- | --- | --- | --- | --- |
|  |  | Confounder | Evidence of placental malaria | Outcome measure | | |
|  |  |  |  | Predictive value of diagnostic | Infant birth weight | Maternal Hb |
| RDT on peripheral blood taken during 2^nd^ trimester | Binary (positive / negative) |  |  | X |  |  |
| Microscopy of peripheral blood taken during 2^nd^ trimester | 1. Binary (positive / negative)  2. Count for positives |  |  | X |  |  |
| PCR on peripheral blood taken during 2^nd^ trimester | Binary (positive / negative) |  | X |  |  |  |
| RDT on peripheral blood taken during 3^rd^ trimester | Binary (positive / negative) |  |  | X |  |  |
| Microscopy of peripheral blood taken during 3^rd^ trimester | 1. Binary (positive / negative)  2. Count for positives |  |  | X |  |  |
| PCR peripheral blood taken during 3^rd^ trimester | Binary (positive / negative) |  | X |  |  |  |
| RDT on peripheral blood taken during or shortly after delivery | Binary (positive / negative) |  |  | X |  |  |
| Microscopy of peripheral blood taken during or shortly after delivery | 1. Binary (positive / negative)  2. Count for positives |  |  | X |  |  |
| PCR result on peripheral blood taken during or shortly after delivery | Binary (positive / negative) |  | X |  |  |  |
| Placental histology for evidence of infection | Binary (positive / negative) |  | X |  |  |  |
| Diagnostic results of any additional visits to clinic (eg symptomatic infection) | Binary (positive / negative) |  | X |  |  |  |
| Maternal Hb at delivery | Continuous |  |  |  |  | X |
| Infant birth weight at delivery | Continuous |  |  |  | X |  |
| Study site | Nominal | X |  |  |  |  |
| Age | Continuous | X |  |  |  |  |
| Gravidity | Ordinal | X |  |  |  |  |
| SP treatment | Binary (yes/no) | X |  |  |  |  |
| Iron & folate supplementation | Binary (yes/no) | X |  |  |  |  |
| Bed net use | Binary (yes/no) | X |  |  |  |  |

The following definitions will be used:

- Evidence of placental malaria: a positive PCR result is obtained for any peripheral blood sample and / or there is evidence of placental infection by histology
- RDT positive: a positive RDT result is obtained from one or more blood samples
- Microscopy positive: parasites are detected by microscopy (thick film) in one or more of the blood samples

The accuracy of RDTs and peripheral microscopy at detecting placental malaria will be assessed by considering the sensitivity, specificity, positive predictive value and negative predictive value of each diagnostic compared to evidence of placental malaria. This analysis will be conducted for all samples combined and also separately for each study location. Marginal generalized linear models will be developed to assess the impact of confounders on predictive values.

The impact of RDT screening and treatment compared to standard IPT on infant birth weight and maternal Hb will be assessed for non-inferiority by testing the hypotheses:

1. Mean birth weight of infants whose mothers were RDT positive & treated with ACT/quinine ≥ mean birth weight of infants whose mothers were RDT negative (and received SP)
2. Mean Hb of mothers who were RDT positive & treated with ACT/quinine ≥ mean Hb of mothers who were RDT negative (and received SP)

Standard statistical techniques such as t-tests, ANOVA and regression modeling will be used to test these hypotheses and investigate the potential impact of confounders on birth weight and maternal Hb.

**11.3 Sample size considerations**

The target sample size for the number of participants at each study site is calculated to test the hypothesis Ho: [PPV ≤ (disease prevalence + α) or NPV ≤ (1-disease prevalence)] with 80% power and 5% significance at each study location. For the higher transmission sites of Uganda and Burkina Faso α=0.4, while α=0.3 for Nigeria. Previously published estimates of sensitivity (65%) and specificity (98%) were used in these calculations.

Using the target sample sizes outlined below the study will be powered to detect changes in infant birth weight and maternal Hb of >85g and 0.34g/dl, respectively. The sample size for the Burkina Faso site should also allow detection of changes in infant birth weight (>123g) with sufficient power. Based on published data, other sites will not be individually powered to detect likely changes in birth weight (~123g) and none of the sites will be individually powered to detected likely changes in maternal Hb (~0.4g/dl). However this will not prohibit any of the statistical analysis planned.

We estimate that the statistical requirements of the study can be met by enrolling 345 women at the high-transmission Ugandan site, 460 women at the low-transmission Nigerian site, and 860 women at the seasonal-transmission site in Burkina Faso, for a total sample size of 1,665 participants. This estimate assumes a 15% loss to follow-up at each study location.

**11.4 Participant enrollment and follow-up**

Recruitment and enrollment will include all eligible pregnant women who present for routine antenatal care in their second or third trimester (sections 6, 7.2.1). Recruitment will continue at each site until the target sample size given in Section 9.3 is reached.

**12 QUALITY ASSURANCE**

Quality control and assurance of all diagnostic tests will be conducted according to SOPs and as briefly described in sections 7.6.2.1, 7.6.3.2, 7.6.4 and 7.6.7. To ensure the quality and integrity of data, and the safety of participants, an independent study monitor will be contracted for the duration of the study as in section 8.3.

**13 EXPECTED OUTCOMES OF THE STUDY**

Malaria prevention measures for use during pregnancy, including insecticide-treated nets and IPTp are available and effective. However, increasing parasite resistance to SP- IPTp raises concerns that this approach will soon lose effectiveness. Screening with rapid diagnostic tests (RDTs) may offer an accurate and practical way to identify pregnant women who will benefit from targeted therapy for placental malaria infection. This study proposes to assess the efficacy of well-characterized RDTs in detection of placental malaria, to provide data necessary for future evaluations of screening and targeted treatment as a potential alternative to the failing regimen of IPTp with SP. If RDTs are shown to be accurate in detecting placental malaria, it will provide new evidence to support interventions such as IST (intermittent screening and treatment) in further research and in malaria control policy.

**14 DISSEMINATION OF RESULTS AND PUBLICATION POLICY**

Data collected in this study will be compiled, analyzed and made available to collaborating partners, ministries of health in participating countries, the WHO, and will be prepared for publication in a peer-reviewed journal. Study staff and health center staff will be informed of overall study results after data analysis has been completed. Summarized study results may be posted in the participating health centers for review by participants. Results of the study may be publicized in the future by collaborating partners and stakeholders as part of public health education efforts and malaria control programs.

**15 DURATION OF THE PROJECT**

The duration of participant recruitment and follow-up is expected to take approximately 12 months from the start of study activities at each site, depending on rates of enrollment.

**Table 1. Proposed study timeline**

| Activity | Mo 1-3 | | Mo 4-6 | Mo 7-9 | Mo 10-12 | Mo 13-15 | Mo 16-18 |
| --- | --- | --- | --- | --- | --- | --- | --- |
| Study/site prep, training |  | |  |  |  |  |  |
| Pilot, begin enrollment |  |  |  |  |  |  |  |
| Enroll to sample size |  |  |  |  |  |  |  |
| Participant follow-up |  | |  |  |  |  |  |
| Quality control of diagnostic test results |  | |  |  |  |  |  |
| Completion of PCR |  | |  |  |  |  |  |
| Data cleaning, analysis |  | |  |  |  |  |  |
| Report writing |  | |  |  |  |  |  |

**16 PROBLEMS ANTICIPATED**

**Table 2. Potential study problems and proposed solutions**

| **Potential problem** | **Proposed solution** |
| --- | --- |
| Insufficient sample size, or slower than expected enrollment | 1) Increase number of clinical sites (within the same geographical/population area)  2) Publicize study through locally appropriate communication channels |
| High (>15%) loss to follow-up | 1) Reinforce importance of full follow-up in informed consent discussion and at study visits  2) Increase home visit staff |
| Major discrepancies in validation and quality control of diagnostic test results | 1) Discuss with co-investigators to identify reason/s: poor equipment/lab supplies, personnel error, etc.  2) Address cause with replacement of equipment/supplies, refresher training, personnel replacement if necessary, or other as appropriate |
| Adverse events and/or protocol violations | Report to appropriate ethical committee and DSMB; prevention as appropriate through protocol amendment and/or personnel training |

**17 ETHICAL CONSIDERATIONS AND PARTICIPANT CONFIDENTIALITY**

Ethical approval will be sought from the WHO and from national/institutional review committees in all participating countries. Written informed consent will be obtained from all participants as described in section 7.2. Participants will be identified by coded study numbers rather than names in all data collection forms and electronic databases. No individual identities will be used in any reports or publications resulting from the study. Only study staff and study investigators will have access to the information collected, for purposes of data entry and analysis. At the time of informed consent for study participation, participants will be informed that participation in a research study may involve a loss of privacy; however, for this study no sensitive information will be collected, and only study personnel will have access to the information collected.

REFERENCES

**1.** Steketee RW, Nahlen BL, Parise ME, Menendez C. The burden of malaria in pregnancy in malaria-endemic areas. *Am J Trop Med Hyg.* Jan-Feb 2001;64(1-2 Suppl):28-35.

**2.** WHO/AFRO. *A strategic framework for malaria prevention and control during pregnancy in the African region.* Brazzaville: World Health Organization, Regional Office for Africa; 2004. AFR/MAL/O4/01.

**3.** Desai M, ter Kuile FO, Nosten F, et al. Epidemiology and burden of malaria in pregnancy. *Lancet Infect Dis.* Feb 2007;7(2):93-104.

**4.** Walker-Abbey A, Djokam RR, Eno A, et al. Malaria in pregnant Cameroonian women: the effect of age and gravidity on submicroscopic and mixed-species infections and multiple parasite genotypes. *Am J Trop Med Hyg.* Mar 2005;72(3):229-235.

**5.** Rogerson SJ, van den Broek NR, Chaluluka E, Qongwane C, Mhango CG, Molyneux ME. Malaria and anemia in antenatal women in Blantyre, Malawi: a twelve-month survey. *Am J Trop Med Hyg.* Mar 2000;62(3):335-340.

**6.** Newman RD, Hailemariam A, Jimma D, et al. Burden of malaria during pregnancy in areas of stable and unstable transmission in Ethiopia during a nonepidemic year. *J Infect Dis.* Jun 1 2003;187(11):1765-1772.

**7.** ter Kuile FO, Parise ME, Verhoeff FH, et al. The burden of co-infection with human immunodeficiency virus type 1 and malaria in pregnant women in sub-saharan Africa. *Am J Trop Med Hyg.* Aug 2004;71(2 Suppl):41-54.

**8.** Brabin BJ. An analysis of malaria in pregnancy in Africa. *Bull World Health Organ.* 1983;61(6):1005-1016.

**9.** Archibald HM. The influence of malarial infection of the placenta on the incidence of prematurity. *Bull World Health Organ.* 1956;15(3-5):842-845.

**10.** Jelliffe EF. Low birth-weight and malarial infection of the placenta. *Bull World Health Organ.* 1968;38(1):69-78.

**11.** Spitz AJ. Malaria infection of the placenta and its influence on the incidence of prematurity in eastern Nigeria. *Bull World Health Organ.* 1959;21:242-244.

**12.** Guyatt HL, Snow RW. The epidemiology and burden of Plasmodium falciparum-related anemia among pregnant women in sub-Saharan Africa. *Am J Trop Med Hyg.* Jan-Feb 2001;64(1-2 Suppl):36-44.

**13.** Guyatt HL, Snow RW. Impact of malaria during pregnancy on low birth weight in sub-Saharan Africa. *Clin Microbiol Rev.* Oct 2004;17(4):760-769, table of contents.

**14.** van Geertruyden JP, Thomas F, Erhart A, D'Alessandro U. The contribution of malaria in pregnancy to perinatal mortality. *Am J Trop Med Hyg.* Aug 2004;71(2 Suppl):35-40.

**15.** Uneke CJ. Diagnosis of Plasmodium falciparum malaria in pregnancy in sub-Saharan Africa: the challenges and public health implications. *Parasitol Res.* Feb 2008;102(3):333-342.

**16.** Rogerson SJ, Mkundika P, Kanjala MK. Diagnosis of Plasmodium falciparum malaria at delivery: comparison of blood film preparation methods and of blood films with histology. *J Clin Microbiol.* Apr 2003;41(4):1370-1374.

**17.** Cottrell G, Mary JY, Barro D, Cot M. Is malarial placental infection related to peripheral infection at any time of pregnancy? *Am J Trop Med Hyg.* Dec 2005;73(6):1112-1118.

**18.** Rogerson SJ, Mwapasa V, Meshnick SR. Malaria in pregnancy: linking immunity and pathogenesis to prevention. *Am J Trop Med Hyg.* Dec 2007;77(6 Suppl):14-22.

**19.** Mockenhaupt FP, Ulmen U, von Gaertner C, Bedu-Addo G, Bienzle U. Diagnosis of placental malaria. *J Clin Microbiol.* Jan 2002;40(1):306-308.

**20.** Mockenhaupt FP, Rong B, Till H, et al. Submicroscopic Plasmodium falciparum infections in pregnancy in Ghana. *Trop Med Int Health.* Mar 2000;5(3):167-173.

**21.** Adegnika AA, Verweij JJ, Agnandji ST, et al. Microscopic and sub-microscopic Plasmodium falciparum infection, but not inflammation caused by infection, is associated with low birth weight. *Am J Trop Med Hyg.* Nov 2006;75(5):798-803.

**22.** Guthmann JP, Ruiz A, Priotto G, Kiguli J, Bonte L, Legros D. Validity, reliability and ease of use in the field of five rapid tests for the diagnosis of Plasmodium falciparum malaria in Uganda. *Trans R Soc Trop Med Hyg.* May-Jun 2002;96(3):254-257.

**23.** Hopkins H, Bebell L, Kambale W, Dokomajilar C, Rosenthal PJ, Dorsey G. Rapid diagnostic tests for malaria at sites of varying transmission intensity in Uganda. *J Infect Dis.* Feb 15 2008;197(4):510-518.

**24.** de Oliveira AM, Skarbinski J, Ouma PO, et al. Performance of malaria rapid diagnostic tests as part of routine malaria case management in Kenya. *Am J Trop Med Hyg.* Mar 2009;80(3):470-474.

**25.** Houze S, Boly MD, Le Bras J, Deloron P, Faucher JF. PfHRP2 and PfLDH antigen detection for monitoring the efficacy of artemisinin-based combination therapy (ACT) in the treatment of uncomplicated falciparum malaria. *Malar J.* 2009;8:211.

**26.** WHO. *Guidelines for the treatment of malaria* 2006. WHO/HTM/MAL/2006.1108.

**27.** WHO/FIND/CDC/TDR. *Malaria rapid diagnostic test performance: Results of WHO product testing of malaria RDTs: Round 1 (2008).* http://apps.who.int/tdr/svc/publications/tdr-research-publications/rdt-performance 2009.

**28.** Leke RF, Djokam RR, Mbu R, et al. Detection of the Plasmodium falciparum antigen histidine-rich protein 2 in blood of pregnant women: implications for diagnosing placental malaria. *J Clin Microbiol.* Sep 1999;37(9):2992-2996.

**29.** Mankhambo L, Kanjala M, Rudman S, Lema VM, Rogerson SJ. Evaluation of the OptiMAL rapid antigen test and species-specific PCR to detect placental Plasmodium falciparum infection at delivery. *J Clin Microbiol.* Jan 2002;40(1):155-158.

**30.** Singer LM, Newman RD, Diarra A, et al. Evaluation of a malaria rapid diagnostic test for assessing the burden of malaria during pregnancy. *Am J Trop Med Hyg.* May 2004;70(5):481-485.

**31.** Malhotra I, Dent A, Mungai P, Muchiri E, King CL. Real-time quantitative PCR for determining the burden of Plasmodium falciparum parasites during pregnancy and infancy. *J Clin Microbiol.* Aug 2005;43(8):3630-3635.

**32.** Mockenhaupt FP, Bedu-Addo G, von Gaertner C, et al. Detection and clinical manifestation of placental malaria in southern Ghana. *Malar J.* 2006;5:119.

**33.** WHO/TDR. *Assessment of the safety of artemisinin compounds in pregnancy* 2006. WHO/CDS/MAL/20903.1094, WHO/GMP/TDR/Artemisinin/07.1.

**34.** WHO. *World Malaria Report 2008*: World Health Organization; 2008. WHO/HTM/GMP/2008.1.

**35.** Schultz LJ, Steketee RW, Macheso A, Kazembe P, Chitsulo L, Wirima JJ. The efficacy of antimalarial regimens containing sulfadoxine-pyrimethamine and/or chloroquine in preventing peripheral and placental Plasmodium falciparum infection among pregnant women in Malawi. *Am J Trop Med Hyg.* Nov 1994;51(5):515-522.

**36.** Verhoeff FH, Brabin BJ, Chimsuku L, Kazembe P, Russell WB, Broadhead RL. An evaluation of the effects of intermittent sulfadoxine-pyrimethamine treatment in pregnancy on parasite clearance and risk of low birthweight in rural Malawi. *Ann Trop Med Parasitol.* Mar 1998;92(2):141-150.

**37.** Parise ME, Ayisi JG, Nahlen BL, et al. Efficacy of sulfadoxine-pyrimethamine for prevention of placental malaria in an area of Kenya with a high prevalence of malaria and human immunodeficiency virus infection. *Am J Trop Med Hyg.* Nov 1998;59(5):813-822.

**38.** Shulman CE, Dorman EK, Cutts F, et al. Intermittent sulphadoxine-pyrimethamine to prevent severe anaemia secondary to malaria in pregnancy: a randomised placebo-controlled trial. *Lancet.* Feb 20 1999;353(9153):632-636.

**39.** Njagi JK, Magnussen P, Estambale B, Ouma J, Mugo B. Prevention of anaemia in pregnancy using insecticide-treated bednets and sulfadoxine-pyrimethamine in a highly malarious area of Kenya: a randomized controlled trial. *Trans R Soc Trop Med Hyg.* May-Jun 2003;97(3):277-282.

**40.** Kublin JG, Dzinjalamala FK, Kamwendo DD, et al. Molecular markers for failure of sulfadoxine-pyrimethamine and chlorproguanil-dapsone treatment of Plasmodium falciparum malaria. *J Infect Dis.* Feb 1 2002;185(3):380-388.

**41.** Alker AP, Mwapasa V, Purfield A, et al. Mutations associated with sulfadoxine-pyrimethamine and chlorproguanil resistance in Plasmodium falciparum isolates from Blantyre, Malawi. *Antimicrob Agents Chemother.* Sep 2005;49(9):3919-3921.

**42.** Omar SA, Adagu IS, Warhurst DC. Can pretreatment screening for dhps and dhfr point mutations in Plasmodium falciparum infections be used to predict sulfadoxine-pyrimethamine treatment failure? *Trans R Soc Trop Med Hyg.* May-Jun 2001;95(3):315-319.

**43.** Staedke SG, Sendagire H, Lamola S, Kamya MR, Dorsey G, Rosenthal PJ. Relationship between age, molecular markers, and response to sulphadoxine-pyrimethamine treatment in Kampala, Uganda. *Trop Med Int Health.* May 2004;9(5):624-629.

**44.** ter Kuile FO, van Eijk AM, Filler SJ. Effect of sulfadoxine-pyrimethamine resistance on the efficacy of intermittent preventive therapy for malaria control during pregnancy: a systematic review. *JAMA.* Jun 20 2007;297(23):2603-2616.

**45.** Gesase S, Gosling RD, Hashim R, et al. High resistance of Plasmodium falciparum to sulphadoxine/pyrimethamine in northern Tanzania and the emergence of dhps resistance mutation at Codon 581. *PLoS ONE.* 2009;4(2):e4569.

**46.** WHO. *Technical expert group meeting on intermittent preventive treatment in pregnancy (IPTp).* Geneva: World Health Organization; 11-13 July 2007 2007.

**47.** Greenwood B, Alonso P, ter Kuile FO, Hill J, Steketee RW. Malaria in pregnancy: priorities for research. *Lancet Infect Dis.* Feb 2007;7(2):169-174.

**48.** Brabin BJ, Wasame M, Uddenfeldt-Wort U, Dellicour S, Hill J, Gies S. Monitoring and evaluation of malaria in pregnancy - developing a rational basis for control. *Malar J.* 2008;7 Suppl 1:S6.

**49.** Council for International Organization of Medical Sciences. International Ethical Guidelines for Biomedical Research Involving Human Subjects. http://www.cioms.ch/frame_guidelines_nov_2002.htm. Accessed April, 2010.

**50.** White NJ. Intermittent presumptive treatment for malaria. *PLoS Med.* Jan 2005;2(1):e3.

**51.** Menendez C, Mayor A. Congenital malaria: the least known consequence of malaria in pregnancy. *Semin Fetal Neonatal Med.* Jun 2007;12(3):207-213.

**52.** Falade C, Mokuolu O, Okafor H, et al. Epidemiology of congenital malaria in Nigeria: a multi-centre study. *Trop Med Int Health.* Nov 2007;12(11):1279-1287.

**53.** Perrault SD, Hajek J, Zhong K, et al. Human immunodeficiency virus co-infection increases placental parasite density and transplacental malaria transmission in Western Kenya. *Am J Trop Med Hyg.* Jan 2009;80(1):119-125.

**54.** Mwangoka GW, Kimera SI, Mboera LE. Congenital Plasmodium falciparum infection in neonates in Muheza District, Tanzania. *Malar J.* 2008;7:117.

**55.** Global Forum for Health Research and WHO. *Research issues in sexual and reproductive health for low- and middle-income countries* 2007.

**56.** McCosker H. Undertaking sensitive research: issues and strategies for meeting the safety needs of all participants. *Forum: Qualitative Social Research.* Feb 2001 2001;2(1).

**57.** WHO/FIND/CDC. Methods manual for laboratory quality control testing of malaria rapid diagnostic tests. Aug 2008; version 5(a):Manual of SOPs for:lab-based quality control testing of malaria RDTs using stored dilutions of malaria parasites; and preparation of quality control samples from malaria parasite field collections. Available at: http://www.wpro.who.int/NR/rdonlyres/B5446BF5-BCFA-427D-B9FE-CEA57D36B92B/0/RDTQCMethodsManualV4final3WEBVERSION.pdf. Accessed 1 Sep, 2008.

**58.** WHO. Malaria Microscopy Quality Assurance Manual version 1. Feb 2009.
