# Supplementary material for: HRP2 and pLDH-Based Rapid Diagnostic Tests, Expert Microscopy, and PCR for Detection of Malaria Infection during Pregnancy and at Delivery in Areas of Varied Transmission: A Prospective Cohort Study in Burkina Faso and Uganda
Source: PLoS One. 2016 Jul 5;11(7):e0156954. doi: 10.1371/journal.pone.0156954 (PMC4933335; doi:10.1371/journal.pone.0156954)
Supplement: S2 File — (DOC) [file pone.0156954.s003.doc]

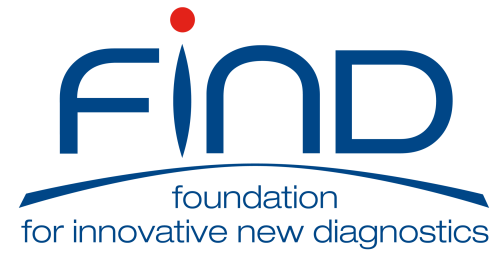


| **PROTOCOLE DE L’ETUDE:**  **Site d’étude du Burkina Faso** |
| --- |
|  |
| **Tests de diagnostic rapide du paludisme (TDR) pendant la grossesse:**  **la détection du paludisme placentaire** |
|  |
| Version et date: 0.7 / 5 avril 2010  (approuvee par le Comite d’Ethique de la Ministere de la Sante, Centre Muraz, Burkina Faso, 18 oct 2010) |
| Sites de l’étude: Burkina Faso, Nigeria, Ouganda |

| Principal Investigateur : Prof JB OUEDRAOGO  Directeur de Recherche, IRSS, Direction Régionale de l'Ouest  399, Avenue de la Liberté  01 BP 545 Bobo-Dioulasso 01, BURKINA FASO  Tél : +226 20981880  Fax +226 20974868  Email: jbouedraogo.irssbobo@fasonet.bf *or* [jbouedraogo@gmail.com](mailto:jbouedraogo@gmail.com) | |
| --- | --- |
|  |  |
|  | |


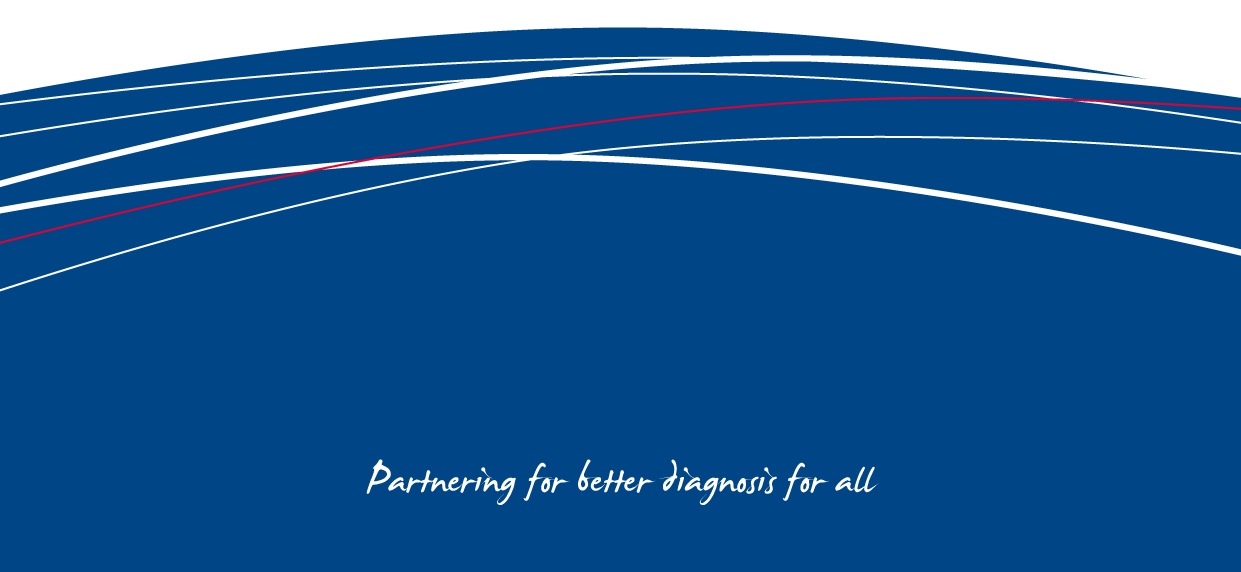

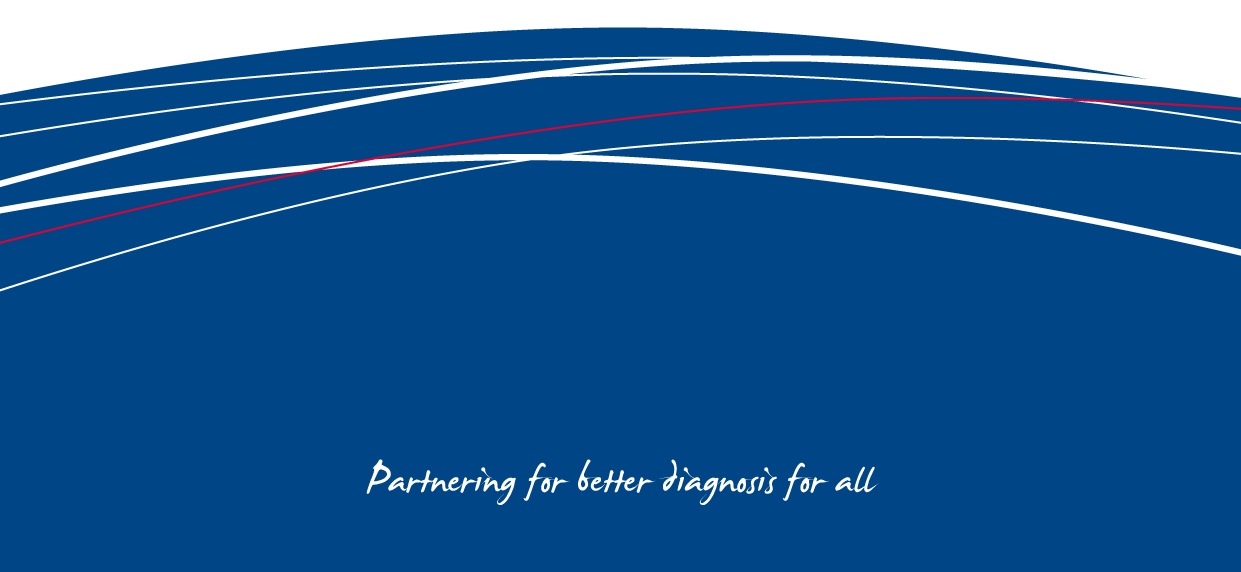


*Technical and Financial Agency:*

**FIND** Geneva tel: +41 22 710 0590 fax: +41 22 710 0599 www.finddiagnostics.org

**L'énoncé de confidentialité:**

The information contained in this document, especially unpublished data, is the property of FIND (or under its control) and may not be reproduced, published or disclosed to others without written authorization.Les informations contenues dans ce document, données non publiées en particulier, est la propriété de FIND (ou sous son contrôle) et ne peut être reproduite, publiée ou divulguée à des tiers sans autorisation écrite.

**1 INFORMATIONS GENERALES**

**Titre Protocole: Les tests de diagnostic rapide (TDR) pendant la grossesse: la détection du paludisme placentaire**

**Numéro et date de la version et date: 0.7 du 5 avril 2010**

**Chercheurs:**

1. Jean-Bosco Ouedraogo, MD, PhD

Directeur de Recherche, IRSS, Direction Régionale de l'Ouest

399, Avenue de la Liberté
01 BP 545 Bobo-Dioulasso 01, BURKINA FASO
Tél : +226 20981880  Fax +226 20974868
Email: [jbouedraogo.irss@fasonet.bf](mailto:jbouedraogo.irss@fasonet.bf) ou [jbouedraogo@gmail.com](mailto:jbouedraogo@gmail.com)

1. Issaka Zongo, MD

IRSS, Direction Régionale de l'Ouest
399, Avenue de la Liberté
01 BP 545 Bobo-Dioulasso 01, BURKINA FASO
Tél : +226 20981880  Fax +226 20974868

Email : issaka.zongo@lshtm.ac.uk ou zongo_issaka@yahoo.fr

1. Heidi Hopkins, MD, MPH

FIND Uganda, Plot 45B/47A Lumumba Ave, PO Box 34663

Kampala, UGANDA

Tel: +256 772 911236

E-mail: [heidi.hopkins@finddiagnostics.org](mailto:heidi.hopkins@finddiagnostics.org)

1. Miriam Nakalembe, MBChB

Department of Obstetrics & Gynecology, Makerere University Faculty of Medicine

Kampala, UGANDA

Tel: +256 753 857433

Email: ivuds@yahoo.com

1. David Bell, MBBS, PhD

GMP/WHO

World Health Organization/Organisation Mondiale de la Santé

20, avenue Appia

Geneva, SWITZERLAND

bellda@who.int

Tel: +41 792 691 638

1. Jane Cunningham, MD, MPH

Technical Officer

WHO/IER/TDR/DQR

World Health Organization/Organisation Mondiale de la Santé

20, avenue Appia

1211 Geneva 27, SWITZERLAND

[cunninghamj@who.int](mailto:cunninghamj@who.int)

Tel: +41 (22) 791 2230

1. Wellington Oyibo, PhD

Tropical Disease Research Laboratory, Department of Medical Microbiology and Parasitology

College of Medicine, University of Lagos

Idi-Araba, Lagos , NIGERIA

Tel: +234-8035374004

E-mail: [wellao@yahoo.com](mailto:wellao@yahoo.com)

**PAGE DES CERTIFICATIONS ET SIGNATURES DES INVESTIGATEURS ET SPONSORS**

En signant cette page, je suis d'accord pour réaliser l'étude en conformité avec la version actuelle du protocole, avec la réglementation applicable et avec la politique institutionnelle.

Je veillerai à ce que les exigences relatives à l'obtention de l’approbation du comité d'éthique institutionnel sont remplies. Je vais signaler sans tarder au comité d’éthique institutionnel tous les changements dans l'activité de recherche.

Je suis d’accord pour informer mes collègues FIND, TDR et le co-chercheurs avant d'apporter des changements dans le protocole.

Je respecterai la confidentialité du protocole de recherche.
Je suis d'accord pour que tous les collègues et les employés à la conduite de l'étude soient informés de leurs obligations à respecter les exigences ci-dessus.

_________________________________________________________________________________

Prof Jean-Bosco Ouedraogo, Investigateur Principal, Site Burkina Faso Date

_________________________________________________________________________________

Dr Heidi Hopkins, Coordinatrice des Sites Date

_________________________________________________________________________________

Dr David Bell, Représentatif du Sponsor: FIND Date

_________________________________________________________________________________

Dr Jane Cunningham, Représentative du Sponsor: TDR Date

**2 APERCU DE L’ETUDE ET RESUME DU PROTOCOLE**

La population d'étude: Les femmes se présentant pour des soins prénatals de routine dans aux deuxième et troisième trimestres de la grossesse.

Number of Sites: Antenatal clinics at ≥3 sites of varied malaria transmission intensity in Africa; proposed collaborating sites in Burkina Faso, Nigeria, and Uganda. Nombre de sites : 3 formations sanitaires en Afrique où l'intensité de la transmission du paludisme varie; les sites collaborateurs proposés sont le Burkina Faso, le Nigeria et l'Ouganda. (NB : A présent les fonds sont disponibles pour faire l’étude dans deux sites, au Burkina Faso et en Ouganda ; si à l’avenir les le ressources permettent, on poursuivra aussi l’étude sur le site nigerian.)

Study Duration: Approximately 12 months from the start of study activities at each site, depending on rates of enrollment. Durée de l'étude: Environ 12-18 mois après le début de l'étude des activités de chaque site, selon le taux de recrutement.

General objective: To determine whether screening pregnant women for malaria with RDTs may detect placental infection and predict risk of poor birth outcomes due to malaria in areas of varied malaria transmission in Africa. Objectif général: Pour déterminer si le dépistage du paludisme chez les femmes enceintes avec les tests de diagnostic rapides (TDRs) peut détecter l'infection placentaire et prédire le risque de résultats défavorables de l'accouchement à cause du paludisme dans les zones de transmission variable du paludisme en Afrique.

Les objectifs primaires:

1. Comparer les résultats des TDR, de la microscopie et de la polymerase chain reaction (PCR) effectuée sur du sang périphérique de femmes aux deuxième et troisième trimestres de la grossesse dans les zones d’endémie palustre d'Afrique.
2. To determine the positive and negative predictive values of results of RDTs, microscopy and PCR performed on peripheral blood from women in the second and third trimesters of pregnancy, as compared with evidence of placental infection by histological examination after delivery.Déterminer les valeurs prédictives positives et négatives (VPP et VPN) des résultats des TDR, de la microscopie et de la PCR effectuées sur du sang périphérique de femmes aux deuxième et troisième trimestres de la grossesse.
3. Comparer les VPP et VPN avec les signes d'infection placentaire mis en evidence par l'examen histologique du placenta après l'accouchement.

Les objectifs secondaires:

1. Comparer des résultats de la grossesse (poids à la naissance du nouveau-né, l'hémoglobine maternelle ect.), la prévalence du paludisme placentaire et le risque de paludisme clinique chez les femmes ayant un test de diagnostic rapide positif à celles ayant un test négatif au moment de l'administration du traitement intermittent préventif (TPI) aux deuxième et troisième trimestres de la grossesse. Les femmes ayant un test de diagnostic positif recevront un traitement à base de quinine ou à base d’artemisinine, celles ayant un test de diagnostic rapide négatif recevront le TPI à base de sulfadoxine-pyrimethamine (SP).
2. To collect samples for future laboratory analyses related to malaria in pregnancy, including the measurement of the prevalence of molecular markers of antimalarial resistance in the study cohorts.Prélever des échantillons pour des analyses ultérieures de laboratoire en rapport avec le paludisme pendant la grossesse, y compris la mesure de la prévalence des marqueurs moléculaires de la résistance aux antipaludiques.

**3 CONTEXTE ET JUSTIFICATION**

***3.1 Background Information3.1 Renseignements de base***

*3.1.1 Epidemiology and risks of malaria in pregnancy3.1.1 Epidémiologie et risques de paludisme pendant la grossesse*

Pregnant women are at particular risk of malaria infection and its consequences.  An estimated 50 million women in endemic areas become pregnant each year, and of these approximately half are in sub-Saharan Africa where *Plasmodium falciparum* infections predominate and transmission intensity is significantly higher than in other parts of the world. ^1, 2^ Based on review of cross-sectional data, in the absence of preventive efforts it is estimated that approximately one in four pregnant women in areas of stable malaria transmission in Africa have evidence of infection at the time of delivery. ^3^ In moderate and high transmission areas, younger women and paucigravidae are typically at higher risk than older women and multigravidae, indicating some protective effect of age- and parity-specific immunity. ^4-6^ The risks presented to mother and fetus by malaria infection may be exacerbated by co-existing conditions including malnutrition, other parasitic infections, and HIV infection. ^1, 7^ Les femmes enceintes sont particulièrement à risque d'infection par le paludisme et ses conséquences. Environ 50 millions de femmes dans les zones endémiques tombent enceintes chaque année dont la moitié en Afrique sub-saharienne où prédominent les infections à *Plasmodium falciparum* et ou l'intensité de la transmission est la plus élevée au monde. ^1, 2^ Une revue de données épidémiologiques révèle qu’en l'absence d'efforts de prévention, environ une femme enceinte sur quatre dans les zones de transmission stable du paludisme en Afrique auront des signes d'infection palustre au moment de l’accouchement^3^. Dans les zones à transmission intense à modérée, les jeunes femmes et paucigravidae sont généralement plus à risque que les femmes âgées et multipares, ce qui indique un certain effet protecteur de l'âge et de l'immunité spécifique liée à la parité^4-6^.. Les risques d’accès palustre de la mère et du fœtus peuvent être exacerbés par les états morbides pré-existantes, y compris la malnutrition, les infections parasitaires et l'infection à VIH ^1, 7^.

The negative effects of malaria infection in pregnancy have been recognized for decades. ^8-11^ The specific effects of malaria in pregnancy depend on host immunity, which depends in turn on malaria transmission intensity. ^2^ In areas where malaria transmission is low or unstable, individuals do not acquire immunity with repeated exposure, and pregnant women typically develop symptoms when parasitemic.  In these epidemiologic conditions, important risks of malaria in pregnancy include spontaneous abortion, premature delivery, stillbirths, and maternal death from severe malaria. In contrast, in areas where malaria transmission is moderate to high and stable, adults develop partial immunity through repeated exposure, and most malaria infections are asymptomatic.  Under these conditions, pregnant women are at risk for anemia (including severe anemia with potential for maternal death) ^12^ and placental malaria. Placental malaria infection, while often asymptomatic, has been clearly shown to be associated with low birth weight due to both premature delivery and intrauterine growth retardation; low birth weight in turn is associated with higher rates of infant mortality and poor child development. ^1, 2, 13, 14^ Les effets néfastes de l'infection palustre pendant la grossesse ont été documentés depuis des décennies ^8-11^. Les effets spécifiques du paludisme pendant la grossesse dépendent de l’immunité de l'hôte qui dépend à son tour de l'intensité de la transmission du paludisme. ^2^ Dans les zones où la transmission du paludisme est faible ou instable, il n’y a pas d’acquisition de l’immunité par une exposition répétée aux piqures infestantes. Les femmes enceintes développent des symptômes généralement lorsqu’elles sont parasitées. Dans ces conditions épidémiologiques, les conséquences de l'infection palustre pendant la grossesse sont l'avortement spontané, l’accouchement prématuré, la mort in utéro et la mortalité maternelle. En revanche, dans les zones où la transmission du paludisme est modérée à élevée et stable, les adultes développent une immunité partielle due à l‘exposition répétée et la plupart des infections palustres sont asymptomatiques ; ces formes assymptomatiques sont source d'anémie (y compris l'anémie sévère avec un risque de décès maternel) ^12^ et le paludisme placentaire. L’infection placentaire bien que souvent asymptomatique est clairement associée au faible poids de naissance (due à l’accouchement prématuré et au retard de croissance intra-utérin), le faible poids de naissance à son tour étant associé à des taux plus élevés de mortalité infantile et un retard de croissance de l’enfant. ^1, 2 , 13, 14^

*3.1.2 Le diagnostic du paludisme pendant la grossesse*

For purposes of epidemiological investigations, the gold standard for diagnosis of malaria in pregnancy is histopathological examination of the placenta after delivery. ^15-17^ As recently summarized by Rogerson, et al, microscopic observation of histological changes in the placenta, including the presence of hemozoin deposits, may be used to confirm current and previous malaria infections, and a variety of laboratory protocols have been suggested for preparation of such samples. ^16, 18^ A number of studies have documented the low sensitivity of light microscopy of peripheral blood smears for detecting placental infection. ^19^ Importantly, some studies have indicated that submicroscopic malaria infections, identified with PCR or antigen-detection tests, may be associated with negative clinical outcomes including maternal anemia ^19, 20^ and low birth weight. ^21^ However, PCR is not available outside research settings, and antigen-detection tests have not yet been evaluated adequately for use in pregnancy. Pour les enquêtes épidémiologiques, l’examen de référence pour le diagnostic du paludisme pendant la grossesse est l‘examen histopathologique du placenta après l'accouchement. ^15-17^ Rogerson, et al, ont montré que l'observation microscopique des modifications histologiques dans le placenta, y compris la présence des dépôts d’hemozoin peuvent être utilisées pour confirmer les infections antérieures et actuelles et des protocoles de laboratoire ont été proposés pour la préparation de tels échantillons. ^16, 18^ Par ailleurs des études ont démontré la faible sensibilité de la microscopie optique à détecter le paludisme placentaire à partir de frottis fait sur du sang périphérique maternelle. ^19^ Des études ont montré aussi que les infections palustres sub-microscopiques, identifiées par la PCR ou par les antigènes des tests de détection, peuvent être associées à une anémie maternelle ^19, 20^ et à un faible poids de naissance. ^21^ Cependant, la PCR n'est disponible que dans les centres de recherche et sa réalisation en routine n’est pas aisée alors que les tests de détection d'antigènes pendant la grossesse n’ont pas encore été évalués et validés.

Rapid diagnostic tests (RDTs) detect parasite antigen circulating in the blood of infected individuals. Malaria RDTs, which do not require the laboratory infrastructure or expertise of microscopy, are increasingly seen as a reliable alternative for symptomatic case management in virtually all endemic settings. The accuracy of well-made and correctly performed RDTs has been demonstrated in a variety of settings. ^22-25^ The WHO currently recommends that parasite-based diagnosis, with either microscopy or rapid diagnostic tests (RDTs), be used for case management in all malaria-endemic areas except in certain circumstances (eg severe outbreaks proven to be malaria in resource-poor situations, young children in high transmission settings). ^26^ Many malaria control programs in Africa are working to institute national policies of parasite-based diagnosis, and to expand availability of parasite-based diagnosis to more remote areas through the use of RDTs.  The quality of commercially produced RDTs is formally evaluated by product testing and lot-testing through the WHO/FIND malaria RDT evaluation program in collaboration with the US Centers for Disease Control and Prevention and a network of other laboratories ( [http://www.wpro.who.int/sites/rdt/who_rdt_evaluation/](http://translate.google.com/translate?hl=en&sl=en&tl=fr&prev=_t&u=http://www.wpro.who.int/sites/rdt/who_rdt_evaluation/) ). ^27^ In addition, the WHO/FIND program facilitates lot testing of RDTs procured by national malaria control programs through a network of regional laboratories ( [http://www.wpro.who.int/sites/rdt/who_rdt_evaluation/lot_testing.htm](http://translate.google.com/translate?hl=en&sl=en&tl=fr&prev=_t&u=http://www.wpro.who.int/sites/rdt/who_rdt_evaluation/lot_testing.htm) ).Les Tests de Diagnostic Rapides du paludisme (TDR) permettent de détecter l'antigène circulant du parasite dans le sang des sujets parasités. Les TDR du paludisme ne nécessitant pas d'infrastructure de laboratoire ou l'expertise de la microscopie sont de plus en plus considérés comme une alternative fiable pour le diagnostic et la prise en charge des cas de paludisme clinique dans toutes les zones d’endémie. La précision et la performance des test de diagnostic du paludisme bien faits ont été bien démontrées dans diverses conditions épidémiologique^22-25^. L'OMS recommande la confirmation des cas (soit par le diagnostic microscopique, soit par les TDRs) pour la prise en charge du paludisme dans les zones d’endémie sauf dans certaines circonstances (exemple : graves flambées avérées de paludisme dans les zones ou les ressources sont limitées, chez les jeunes enfants dans des régions à transmission élevée). ^26^ De nombreux programmes de lutte contre le paludisme en Afrique travaillent à la mise en place de politiques nationales de prise en charge du paludisme basée sur la confirmation des cas ; ils travaillent aussi à la disponibilisation des outils diagnostics surtout les TDRs dans les régions les plus réculées. La qualité des TDRs utilisés est formellement assurée par les tests des produits et des tests de lots par le biais du programme d’évaluation de la Fondation for Innovative New Diagnosis (FIND) de l’OMS/TDR en collaboration avec le Centre de Contrôle et de prévention des maladies des Etats Unis et d’autres réseaux de laboratoires partenaires ([http://www.wpro.who.int/sites/rdt/who_rdt_evaluation/](http://translate.google.com/translate?hl=en&sl=en&tl=fr&prev=_t&u=http://www.wpro.who.int/sites/rdt/who_rdt_evaluation/) ). ^27^ En outre, le programme OMS / FIND facilite le test des lots de TDRs fournis aux programmes nationaux à travers un réseau de laboratoires régionaux ( [http://www.wpro.who.int/sites/rdt/who_rdt_evaluation/lot_testing.htm](http://translate.google.com/translate?hl=en&sl=en&tl=fr&prev=_t&u=http://www.wpro.who.int/sites/rdt/who_rdt_evaluation/lot_testing.htm) ).

We have identified seven publications on six studies that evaluated RDTs for detection of malaria infections in peripheral and/or placental blood at delivery. ^19, 28-32^ In general, these studies show that at delivery, the correlation of RDT results (and especially histidine-rich protein 2 [HRP2] RDT results) in peripheral blood with evidence of placental infection falls somewhere between that of microscopy and PCR.   However, none of the published studies compared birth outcomes or placental findings with testing performed during gestation (as opposed to at delivery), and none reported results of placental histology.  More recently, a study in southwest Uganda monitored pregnant women weekly with microscopy, HRP2-based RDT and PCR, and found an RDT sensitivity of 65% and specificity of 99% when compared with PCR on the same peripheral blood sample.[Mehul Dhorda, personal communication]  Another recently-completed study from Ghana compared three treatment groups: one group of pregnant women received IPTp with SP, the second received SP only if the screening RDT was positive, and the third group received artesunate+amodiaquine if the screening RDT was positive.  Preliminary results from this study show no statistically significant difference among the groups in severe maternal anemia or risk of low birth weight.[Harry Tagbor, personal communication]  In summary, as yet there is no confirmed sensitive and practical approach to diagnosing placental malaria during gestation, at a time that more efficacious preventive measures could be appropriately targeted; but evidence to date on the potential for RDTs to fill this role is promising.

Sept publications sur six études ayant évaluées les TDRs du paludisme pour la détection des infections palustres sur du sang périphérique maternelle et sur le sang placentaire à l’accouchement ont été identifiées.^19 , 28-32^ En général, ces études montrent que lors de l'accouchement, la corrélation des résultats des TDRs (et surtout les résultats de la histidine-riche protéine 2 [HRP2] dans le sang périphérique avec des signes d'infection placentaire se situe entre celle de la microscopie et de la PCR ; cependant aucune des études publiées n’a comparé les parametres de l’accouchement (poids de naissance par exemple) et les résultats de l’examen histologique du placenta avec ceux obtenus au cours de la grossesse (microscopie de sang périphérique maternel). Aucune étude n’a rapporté les résultats de l’histologie du placenta. Plus récemment, une étude menée au Sud-Ouest de l'Ouganda ayant suivi des femmes enceintes par semaine avec la microscopie, le TDR par HRP2 et la PCR a montré une sensibilité du TDR de 65% et une spécificité de 99% par rapport à la PCR du même échantillon de sang périphérique. [Méhul Dhorda, communication personnelle]. Une étude récente au Ghana a comparé trois groupes de traitement: un premier groupe de femmes enceintes ayant reçu le TPI avec la SP, un second groupe ayant reçu la SP si le TDR est positif, et un troisième groupe ayant reçu de l’artésunate + amodiaquine si le TDR de dépistage est positif. Les résultats préliminaires de cette étude ne montrent pas de différence statistiquement significative entre les groupes pour l'anémie sévère et le risque de faible poids de naissance. [Harry Tagbor, communication personnelle]. Cependant ils ne sont pas encore confirmés et il n’y a pas d’approche pratique et sensible de diagnostic du paludisme placentaire pendant la grossesse pour permettre la mise en place de mesures préventives ciblées mais le potentiel des TDRs pour ce diagnostic est prometteur.

*3.1.3 La prévention et les méthodes de contrôle du paludisme pendant la grossesse*

Compte tenu des effets néfastes du paludisme pendant la grossesse, de l’insuffisance/inadéquation des infrastructures de santé et des stratégies de contrôle du paludisme dans les zones à transmission stable en Afrique Sub-Saharienne, l’Organisation Mondiale de la Santé (OMS) recommande la stratégie suivante pour la prévention et la lutte contre le paludisme : le traitement préventif intermittent pendant la grossesse (TPI), l’utilisation de moustiquaires et matériaux imprégnés d’insecticides à longue durée d’action (MII), et la prise en charge des cas d’anémie et de paludisme par des antimalariques efficaces. ^2^ Pour le traitement curatif du paludisme aux deuxième et troisième trimestres de la grossesse en Afrique, l'OMS recommande actuellement l’utilisation des combinaisons à base d'artémisinine (CTA) sur la base des résultats d’étude cliniques d'efficacité et de sécurité.^26, 33^ Pour la prévention du paludisme, l'OMS recommande que dans les zones de forte transmission, le TPI avec la SP soit administré aux femmes enceintes au moins deux fois au cours du deuxième et troisième trimestres de la grossesse, et trois fois dans le cas des femmes séropositives enceintes. L'efficacité du TPI doit être surveillée à la lumière de la résistance croissante à la SP.^34^ En fin 2006, 33 pays d’Afrique sur 45 avaient adopté une politique nationale de traitement intermittent du paludisme chez la femme enceinte.^34^ Le bénéfice du TPI avec la SP pendant la grossesse a été démontré par des études menées dans les années 1990 et au début des années 2000.^35-39^ L'intervention a clairement été associée à une baisse sensible de la prévalence de l'anémie maternelle, de la prévalence de la parasitémie placentaire, et de l'incidence du faible poids de naissance. Toutefois, une baisse de la sensibilité du parasite à la SP compromet l'efficacité du TPI avec ce médicament.

*3.1.4 Perte potentielle de l'efficacité du TPI avec l’augmentation de la résistance à la SP* Au cours de ces dernières années, la propagation de la résistance à la SP a été démontrée. Les mutations dans les gènes de la synthase dihydropteroate (DHPS) et de la dihydrofolate réductase (DHFR) du parasite conduisent à la résistance à la sulfadoxine pyriméthamine ; les parasites avec de multiples mutations dans les deux gènes sont frequents dans toute l'Afrique orientale et australe,^40^ y compris dans les populations des femmes enceintes,^41^ et semblent avoir une corrélation négative (traitement moins efficace) en particulier chez les personnes ayant acquis peu d’immunité contre le paludisme. ^42, 43^ Une revue d’études faite en 2007 a montré la persistance de l’effet bénéfique du TPI avec la SP meme dans des régions ou le taux d’échec est très élevé. ^44^ Toutefois, une récente étude menée en Tanzanie a identifié et documenté une nouvelle «triple mutation associée à un taux d'échec élevé à la SP au cours de traitement de nourrissons et d’enfants malades.^45^ Enfin des données sur la baisse de l'efficacité du TPI à la SP commencent à émerger. [Stephen Rogerson, données non publiées]

L’apparition et la propagation de la . résistance à la SP ^2, 46-48^ ayant pour conséquence potentielle qui est la perte de l'efficacité du TPI à la SP, ainsi que l’apparition dans la littérature de données sur la baisse de la transmission du paludisme dans de nombreuses régions d'Afrique obligent à repenser les rapports risques / bénéfices des méthodes de prévention, d’où l’appel de l'OMS et d'autres partenaires à une recherche sur de nouvelles approches pour la prévention du paludisme pendant la grossesse. ^2, 46-48^

## 3.2 Justification de l’étude

Les mesures de prévention du paludisme chez les femmes enceintes sont essentielles et disponibles, mais l’augmentation de la résistance à la SP compromet dangereusement l'efficacité du TPI à base de cette molécule ; des médicaments alternatifs sont alors envisagés. Cependant l’utilisation à grande échelle des traitements disponibles pour des cas présomptifs risque d’augmenter la pression médicamenteuse et précipiter la baisse de l’éfficacité de ces médicaments. Une approche alternative serait de procéder à un dépistage au moyen des tests de diagnostic rapide pour mieux cibler les sujets qui en ont réellement besoin (sujets ou femmes enceintes assymptomatiques). La microscopie optique du sang périphérique maternel sous-estime les cas alors que la PCR n’est pas disponible en routine dans les formations sanitaires. Des résultats préliminaires montrent que la détection de l’antigène du parasite dans le sang périphérique maternel peut fournir un indicateur fiable et prédire les résultats de la grossesse. Par conséquent, la détection des cas par les tests de diagnostic rapide peut offrir une alternative précise et pratique pour identifier les femmes enceintes qui vont bénéficier de thérapies ciblées contre l’infection palustre placentaire. Le seuil de détection des antigènes varient considérablement d'un test de diagnostic rapide (TDR) à un autre, et la répartition des antigènes cibles dans la circulation sanguine périphérique pourrait varier, par conséquent, la valeur potentielle des TDR du paludisme placentaire ne peut etre évaluée qu’en utilisant des tests bien caractérisés ; cela aura pour avantage de pouvoir extrapoler les résultats aux autres produits et programmes. L'étude décrite ci-dessous se propose de répondre à cette question.

1. **OBJECTIFS DE L’ETUDE**

## 4.1 Objectif général

Déterminer si la détection du paludisme des femmes enceintes par les tests de diagnostic rapides (TDR) permet de détecter l'infection placentaire et prédire le risque de résultats défavorables à l'accouchement (faible poids de naissance, anémie, etc.)

**4.2 Les objectifs primaires:**

1. Comparer les résultats des TDRs, de la microscopie et de la PCR effectuée sur du sang périphérique de femmes aux deuxième et troisième trimestres de la grossesse dans les zones d’endémie palustre d'Afrique.
2. Déterminer les valeurs prédictives positives et negatives (VPP et VPN) des résultats des TDRs, de la microscopie et de la PCR effectuée sur du sang périphérique de femmes aux deuxième et troisième trimestres de la grossesse.
3. Comparer les VPP et VPN avec les signes d'infection placentaire mis en evidence par l'examen histologique du placenta après l'accouchement.

**4.3 Les objectifs secondaires:**

1. Comparer des résultats de la grossesse (poids à la naissance pour nourrissons, l'hémoglobine maternelle), la prévalence du paludisme placentaire et le risque de paludisme clinique chez les femmes ayant un test de diagnostic rapide du paludisme positif à celles ayant un résultat négatif par les RDTs au moment de l'administration du TPI aux deuxième et troisième trimestres de la grossesse. Les femmes ayant un TDR positif recevront un traitement à base de quinine ou à base d’artemisinine ; celles avec un TDR négatif recevront le traitement préventif intermittent à base de sulfadoxine-pyrimethamine (SP).
2. Prélever des échantillons pour des analyses ultérieures de laboratoire en rapport avec le paludisme pendant la grossesse, y compris la mesure de la prévalence des marqueurs moléculaires de la résistance aux antipaludiques.
3. **CONCEPTION DE L’ETUDE**

(Voir figure 1 à la page suivante.) Les participantes de l’étude seront recrutées au cours de leur consultation prénatale de routine. Seront éligibles celles qui sont éligibles pour le traitement préventif intermittent (TPI) du paludisme à base de la SP selon les recommandations de l’Organisation Mondiale de la Santé (première visite prénatale après le début de la perception des mouvements actifs du fœtus)^2^ et qui auront donné leur consentement éclairé daté et signé ou avec l’empreinte digitale et signature de témoins. Le calendrier habituel du TPI prévoit une dose de SP au deuxième trimestre et une autre dose au troisieme trimestre. Lors de l’administration de chacune de ces deux doses (donc à chaque visite de consultation prénatale), un prélevement veineux d’environ 2 ml sera effectué chez les participantes de l’étude pour la réalisation d’une goutte épaisse/frottis mince, d’un confettis (2-3 spots de sang collectés sur du papier buvard), et deux tests de diagnostic rapide du paludisme (la Histidine Rich Protein 2 (HRP2) et la lactate déshydrogénase plasmodium (pLDH)) qui seront utilisés dans le cas de cette étude. Toutes les participantes qui auront un résultat négatif aux deux (2) tests recevront de la SP ; celles qui auront un résultat positif à au moins un des tests (HRP2 ou pLDH) recevront un traitement à base de quinine ou à base d’artemisinine (selon la politique nationale pour le traitement du paludisme pendant la grossesse au Burkina Faso). Toutes les participantes seront suivies jusqu'à l’accouchement. A l’accouchement de la femme 3 types de prélevement seront réalisés : un prélèvement de 2 ml de sang veineux de la mère – un prélèvement de 2 ml de sang placentaire – et une biopsie placentaire (en 2-3 points). Le sang veineux maternel et placentaire serviront à la confection de goutte épaisse/frottis mince et les confettis (2-3 spots de sang recueillis sur du papier Whatmann) et les tests de diagnostic rapides HRP2 et pLDH; le sang veineux maternel servira aussi à la mesure de l’hémoglobine maternelle. La biopsie du tissu placentaire servira à l’examen histopathologique. Enfin le poids de naissance du nouveau-né sera mesuré.

**Figure 1. Diagramme de flux des patientes pour évaluer l'association des résultats de TDR avec les preuves de l'infection palustre placentaire et l’issue de la grossesse**

**Une femme enceinte se présente pour des soins prénatals de routine,**

**après les premiers mouvements fœtaux et avant le début de l’accouchement**

**Recrutement dans l’étude**

- Obtention du consentement de la participante
- Tous les critères de sélection sont réunis
- Interrogatoire et examen clinique
- Prélevement de sang veineux pour l’hémoglobine, TDRs, goutte épaisse & frottis mince, et stockage en EDTA, culot de cellules rouges et papier-filtre pour PCR
- Prestation de soins standards (moustiquaire imprégnés, fer + folate suppléments, etc) selon les directives nationales

**RDT positif/s**

**(soit HRP2 ou pLDH)**

**TDRs négatifs**

**(HRP2 et pLDH les deux)**

- **Artémisinine ou quinine (selon la politique nationale)**
- La gestion de tout symptôme à la discrétion du clinicien selon les directives nationales et selon les normes de diligence
- **Suivi 2 semaines plus tard pour TPI avec SP**
- **TPI (directement observé**) **avec SP**
- La gestion de tout symptôme à la discrétion du clinicien selon les directives nationales et selon les normes de diligence

**Visite/s ultérieures pour des soins prénatals de routine**

(≥ 1 mois après la visite précédente, et avant le début de l’accouchement)

- Interrogatoire et examen clinique
- Prélevement de sang veineux pour l’hémoglobine, TDRs, goutte épaisse & frottis mince, et stockage en EDTA, culot de cellules rouges et papier-filtre pour PCR
- Prestation de soins standards (moustiquaire imprégné, fer + folate suppléments, etc) selon les directives nationales

**RDT positif/s**

**(soit HRP2 ou pLDH)**

**TDRs négatifs**

**(HRP2 et pLDH les deux)**

- **TPI (directement observé**) **avec SP**
- La gestion de tout symptôme à la discrétion du clinicien selon les directives nationales et selon les normes de diligence
- **Artémisinine ou quinine (selon la politique nationale)**
- La gestion de tout symptôme à la discrétion du clinicien selon les directives nationales et selon les normes de diligence
- **Suivi 2 semaines plus tard pour TDO TPI avec SP**

**Activités à l’accouchement**

- Prélevement de sang maternel veineux (2 ml) pour l’hémoglobine, TDRs, goutte épaisse & frottis mince, et stockage en EDTA, culot de cellules rouges et papier-filtre pour PCR
- Prélevement de sang placentaire (2 ml) pour les TDRs, les frottis, papier filtre pour PCR et stockage
- Echantillon de tissu placentaire pour préparer à l’examen histopathologique
- Mesure de poids à la naissance de l’enfant et de l’hémoglobine maternel
- Mesure d’autres données sur la morbidité/mortalité maternelle/fœtale

1. **POPULATION DE L’ETUDE**

Les participantes à cette étude sont les femmes enceintes se présentant dans les services de consultation prénatale de routine de formations sanitaires publiques ou privées ou missionnaires dans des régions d’Afrique où le traitement intermittent préventif à la sulfadoxine-pyrimethamine fait partie intégrante de la politique de soins de la femme enceinte (selon les directives de l’OMS et la politique nationale de prise en charge du paludisme des programmes nationaux de lutte contre le paludisme). Toute femme enceinte au deuxième ou troisieme trimestre de sa grossesse est éligible pour participer à l’étude. Les sites participant à l‘étude comprennent les services soins et de consultation prénatale au Burkina Faso, au Nigeria et en Ouganda. Des critères spécifiques de sélection des participantes seront les suivantes :

1) Se présentant pour des CPN au deuxième trimestre (après la perception des mouvements actifs du fœtus) ou au troisième trimestre et avant le début du travail. Age between 16 years and 44 years, inclusiv

2) Âge compris entre 16 ans et 44 ans (inclus)

3) Etre prete et capable de participer à toutes les visites et les activités de l’étude pour la durée de la grossesse et à l’accouchement

4) Absence d’histoire d’une réaction sévère aux médicaments à base de sulfamides

5) Absence d’histoire d’une réaction sévere à la quinine ou aux médicaments à base d’artemisinine (selon la politique nationale pour le traitement du paludisme en grossesse)

6) )Absence d'infection à VIH (l’exclusion de ce groupe de participantes se justifie par le fait que les lignes directrices pour la prévention du paludisme pendant la grossesse pour les femmes infectées par le VIH sont diffèrentes de celles des femmes VIH-négatives, et aussi pour éviter que les complications liées au VIH ne soient des facteurs de confusion pour les résultats de la grossesse et des traitements administrés)

7) Absence d’histoire ou de complications obstétricales récentes (par exemple la pré-éclampsie, l’éclampsie, l’hypertension pendant la grossesse, l'hémorragie du post-partum, la preuve de grossesse multiples ect.)

8) Absence de maladies chroniques (e.g. la diabète, la drépanocytose)

9) Absence de signes de maladie aiguë grave nécessitant une hospitalisation ou une référence à un échellon supérieur.

10) Provision of written informed consentProvision d’un consentement éclairé daté et signé ou une empreinte digitale suivie de la date et de la signature d’un témoin.

11) Enrollment Hb ≥7 g/dLTaux d’hémoglobine au recrutement ≥ 7 g / dL

**7 METHODES ET PROCEDURES D’ETUDE**

**7.1 Sites d’étude et personnel**

*7.1.1 Sélection des sites d'étude*

Les sites d'étude dans chaque pays seront des formations sanitaires publiques ou privées ou missionnaires, qui offrent des soins et des consultations pré et périnatale de routine. Les formations sanitaires pour cette évaluation seront sélectionnées sur la base des critères suivants: Difference de l’intensité de la transmission du paludisme des différents sites, l’intérêt des chercheurs locaux et les autorités de santé publique pour l’objectif de la recherche, l'accessibilité du site par l’équipe de recherche, l'intérêt et la capacité du personnel de la formation sanitaire, et le niveau de fréquentation des services de consultation pré et périnatale (pour permettre l'obtention de la taille de l'échantillon désirée) sur une période d'environ 6 mois.

Sur la base des informations actuelles et des accords préliminaires, les sites sélectionnés comprennent: le centre de santé et de promotion sociale de Colsama dans le district sanitaire de Do en zone urbaine de Bobo-Dioulasso (transmission perenne du paludisme avec une recrudescence saisonnière); l’Hopital catholique St Kizito Hôpital et / ou l’hopital catholique Regina Mundide de Lagos au Nigeria (transmission faible et perenne) et l'Hôpital du district de Tororo à l’Est de l'Ouganda (transmission intense et perenne). Si, pour des raisons imprévues, l’étude ne peut etre conduite sur les sites sélectionnés, d'autres sites seront choisis pour préserver la représentativité géographique et l'endémicité du paludisme.

*7.1.2 Le personnel réalisant les activités de cette étude*

Dans cette étude, les activités de soins, consultations prénatales de routine et les accouchements seront prodiguées par le personnel régulièrement employé par les centres de santé participants (formation sanitaire publique ou privée ou missionnaire). Ce personnel comprend habituellement des sages-femmes, des infirmières et autres cadres similaires du personnel de soins de santé. Dans ce protocole, ce personnel est dénommé "personnel du centre de santé."

Les activités spécifiques de l’étude seront réalisées par du personnel embauché pour les besoins de l'étude ou des personnes employées par les coordonnateurs de l'étude. Tout au long de ce protocole, ce groupe de personnel est dénommé "personnel de l'étude." Le personnel de l'étude comprend : des sages-femmes, des infirmieres, des techniciens microscopistes, des biologistes, un gynéco-obstétricien. Tout ce personnel est qualifié par formation et / ou par expérience pour effectuer ce type d’activités mais aussi ont une expérience antérieure en matiere de recherche clinique sur le paludisme.

Le personnel du centre de santé et le personnel de l'étude seront formés pour maitriser le protocole ainsi que toutes les procédures pertinentes avant le début de l'étude. Dans chaque site les deux groupes du personnel travailleront ensemble pour atteindre les objectifs de l'étude. De plus amples détails pour chaque site seront décrits dans les protocoles propres à chaque site.

**7.2 Inclusion dans l’étude**

***7.2.1. Procédure de sélection des participantes***

La consultation prénatale de routine sera assurée par le personnel du centre de santé et de promotion sociale de Colsama. En effet, l’accueil de la patiente et le conseil de groupe seront assurés par une sage femme ou une infirmière membre du personnel du centre. Les conseils de groupe comportent des thèmes aussi variés comme les infections sexuellement transmissibles et le sida, la prévention de la transmission de la mère à l’enfant du VIH (PTME), la planification familiale, la consultation post-natale, le paludisme, l’hygiène et l’assainissement, la diarrhée et le traitement par réhydratation orale; puis interviendra la CPN proprement dite (examen général, gynéco-obstétrique, la recherche de proteine dans les urines etc.) toujours par le personnel du centre (infirmière ou sage-femme). La prise du traitement intermittent préventif à la SP, la provision du fer acide folique et autres anthelminthiques sont les dernières étapes de la visite prénatale de routine. Les femmes aux deuxième ou troisième trimestres de la grossesse étant éligibles, c’est avant la prise des médicaments (SP, fer, acide folique, albendazole) que la potentielle participante sera référée au personnel de l’étude par le personnel du centre.

***7.2.2. Procédure d’inclusion dans l’étude***

Le personnel de l’étude accueil alors la femme dans une salle séparée de la salle de consultation prénatale de routine. Pour obtenir le consentement de la patiente, le personnel de l’étude s’attachera à expliquer le but et les objectifs de l’étude, les procédures de suivi, les examens à faire, la durée du suivi, le respect du protocole et la nécessité d’accoucher de préférence dans la formation sanitaire ou est basée l’équipe de l’étude. Au cas ou elle accepte de joindre l’étude, elle fournira son consentement éclairé signé et daté (ou empreinte digitale avec signature datée d’un témoin lettré). Le personnel de l’étude évalue alors la femme enceinte au regard des critères d’inclusion détaillés à la section 6. Deux situations peuvent se présenter:

***1. La femme répond à tous les critères d’inclusion***

Elle est interrogée pour recueillir des informations socio-démographiques et les antécédents médicaux et gynéco-obstétricaux ; puis elle bénéficie d’un examen clinique.

Ensuite elle est référée au laboratoire pour le prélèvement de 2 ml de sang veineux pour la réalisation des tests de diagnostic rapide du paludisme, la confection de la goutte épaisse frottis mince et d’un confettis (2-3 gouttes de sang collectés sur du papier Whatman).

En cas de TDR positif (HRP2 positif et/ou pLDH positif), les femmes recevront un traitement à base de quinine ou à base d’artemisinine ; la première dose du traitement sera administrée dans le centre, les doses ultérieures seront remises à la femme avec des instructions claires pour une prise à domicile. En cas de TDRs négatifs (HRP2 négatif ET pLDH négatif) les femmes recevront le traitement intermittent préventif (TPI) à base d’une dose de sulfadoxine-pyrimethamine. La prise est directement observée par le personnel de l’étude. Dans l’un ou l’autre des cas, un traitement adjuvent peut etre associé (antipyrétique, antispasmodique, antalgique etc.) Dans tous les cas, après l’administration des traitements (quinine, derivés de l’artemisinine ou TPI-SP), la femme sera remerciée avec des conseils et une indication sur la date de la prochaine visite ou la date probable du terme de la grossesse. Elle sera aussi encouragée à consulter le personnel de l’étude au cas où elle ne se sentira pas bien. Les femmes enceintes ayant bénéficié de traitement curatif à base de quinine/derivés de l’artemisinine seront revues 2 semaines à compter de la date de la dernière dose du traitement pour recevoir leur TPI-SP.

***2. La femme ne répond pas à au moins un des critères d’inclusion***

Elle ne bénéficiera pas des examens de laboratoire, elle recoit son TPI-SP ainsi que son fer + acide folique et le déparasitage (si nécessaire) par l’équipe de l’étude qui la remercie pour sa patience, elle quitte alors l’étude et pourra etre à nouveau référée au personnel du centre pour toute autre prise en charge dictée par son état clinique. Dans le cas d’une femme seropositive, se référer à la section réseau social de prise en charge des femmes enceintes vivant avec le VIH. Pour chaque femme qui est référée mais qui ne répond pas a tous les criteres d’inclusion, la raison pour laquelle elle n’est pas inclue dans l’etude sera inscrite dans le log des sujets sélectionnés non inclues.

***Note sur le depistage du VIH, et cas particulier des femmes enceintes seropositives***

Les conseils lors de la CPN prennent en compte le dépistage du VIH mais le dépistage est tout de meme volontaire. Il fait partie d’un ensemble de themes discutés en groupe avec les femmes enceintes venues pour la consultation prénatale avant la visite proprement dite. Elle est menée par un personnel formé appartenant au centre de santé et aux structures associatives de prise en charge des personnes vivant avec le VIH. Le personnel assurant le conseil-dépistage du VIH est membre d’un réseau social qui assure le lien entre la formation sanitaire et le réseau social de prise en charge des personnes vivant avec le VIH. Les femmes séropositives référées adhèrent à prix modique pour bénéficier d’une prise en charge gratuite (examens biologiques complémentaires et traitement anti retrovirale). Au cours de la consultation prénatale, la femme bénéficie encore d’un conseil personnalisé et approfondi (dans une salle séparée) au cours duquel elle a l’occasion de poser des questions. Pour les femmes qui acceptent de se soumettre au test, deux cas de figure apparaissent :

1. Si la femme accepte de se soumettre au test de dépistage du VIH, elle en saura les résultats ; de ce fait elle ne sera pas référée si elle est séropositive puisque les membres du centre seront formés au protocole et aux critères d’inclusion/exclusion. En ce cas elle est référée par le personnel du poste de santé aux réseaux sociaux de prise en charge des personnes vivants avec le VIH.Si son test est négatif, elle sera référée à l’équipe de l’étude qui continuera son évaluation à la lumière des autres critères d’inclusion.

2. Si elle refuse le test, elle n’est pas éligible à participer à l’étude et elle ne sera pas référée à l’équipe de l’étude, mais elle reste éligible pour tous autres soins de routine fournis par le poste de santé.

***7.2.3 Provison d’un consentement* é*clair*é**

Chaque participante devra fournir un consentement éclairé écrit, signé et daté (annexes A et B) au moment de la sélection. L’administration du consentement sera assurée par un membre qualifié de l’étude. Ce membre réalisera les interviews dans la langue de la femme. Les fiches d’informations du patient ainsi que les fiches de consentement seront approuvées par les comités d’éthiques locaux ainsi que celui de l’OMS. Le formulaire d’information et la fiche de consentement seront disponibles en Anglais (Nigeria, Ouganda) et en français (Burkina Faso) et au besoin dans la langue locale pour chaque site. Si la participante est incapable de lire ou d'écrire, la signature sera remplacée par les empreintes digitales de la patiente et une signature d'un témoin de la procédure de consentement éclairé sera obtenue. Les participantes potentielles auront l'occasion de poser des questions et de discuter des détails de l'étude avec le personnel de l'étude, et sont libres de poser des questions supplémentaires à tout moment pendant ou à la fin des activités de l'étude. Selon les directives burkinabees, dans le cas des participantes dont l’âge est inférieur à 20 ans (16-19 ans pour cette étude), la femme est considérée comme une mineur émancipée et peut consentir de participer sans l’avis d’un parent ou tuteur [*Le code de la famille*, Burkina Faso]; en tout état de cause,^49^ les lignes directrices spécifiques du pays sur l'inclusion des mineurs dans la recherche (y compris les lignes directrices relatives aux mineurs émancipés, et / ou co-consentement d'un parent ou un tuteur) seront strictement suivies pour permettre la participation des individus dans ce groupe vulnérable, et donc de permettre l'application future de données et les avantages potentiels de cette étude au même groupe. Si la participante répond à toutes les critères d'inscription, elle sera inscrite (Annexe C).

**7.3 Activités de l’étude lors de la visite initiale et collection de données**

*7.3.1 Collection de données cliniques*

At the time of enrollment, information will be collected from each participant on her medical and pregnancy history, and a brief clinical assessment will be performed by a health center staff clinician (see sample data collection form, Appendix D). La participante sera référée par l’équipe du centre qui aura prodigué tous les soins standard offerts lors d’une CPN à l’exception de l’administration du TPI-SP, du fer acide folique et des anthelmintiques. A l’inclusion, des informations seront recueillies auprès de chaque participante sur ses antécédents médicaux, chirurgicaux, gynéco-obstétriques et sur la grossesse actuelle. Une brève évaluation clinique sera effectuée par le personnel sage-femme ou infirmier du projet (voir formulaire de collecte de données, Annexe D).

*7.3.2 Laboratory sample collection7.3.2 Collection des échantillons de laboratoire*

Study staff will collect blood from participants for use in study laboratory procedures, and will prepare the samples and conduct the diagnostic tests described; all other care will be provided by health center staff (ie clinicians, typically midwives and nurses, employed by the health center).  Each participant will provide a 2 mL (two milliliter) venous blood sample for HemoCue hemoglobin (Hb) measurement, preparation of two RDTs, preparation of thick and thin smears for expert light microscopy, and storage in a microtainer and on filter paper for PCR (see further details in section 7.6 below). Hb and RDT results will be used in clinical management as in section 7.3.3 and 7.6.1 below; expert microscopy and PCR results will not be immediately available and will not be used in management. Le personnel de l'étude prélevera le sang de la participante pour la réalisation des examens de laboratoire et tests prévus dans le présent protocole ; Tout autre prélèvement en dehors de ceux spécifiés dans le présent protocole sera réalisé par le personnel du centre. Chaque participante devra fournir deux mililitres (2 ml) de sang veineux dans un tube EDTA pour la mesure du taux d’hémoglobine avec un HemoCue (hémoglobine Hb), la préparation de deux TDRs, la préparation d’une goutte épaisse / frottis mince pour la microscopie optique, la réalisation d’un confettis (2-3 gouttes de sang sur du papier Whatman) et le stockage du culot dans un Microtainer et pour la réalisation de la PCR (voir détails dans la section 7.6 ci-dessous). Le taux d’hémoglobine et les résultats des TDRs seront utilisés pour la prise en charge des patientes ; le résultat de la microscopie et les résultats de la PCR ne seront pas immédiatement disponibles et ne seront pas utilisés dans la prise en charge de la patiente.

*7.3.3 Administration of antimalarial medicines7.3.3 Administration des médicaments antipaludiques par le personnel du projet*

If both RDT results are negative, the participant will receive a complete dose of SP by directly observed therapy (DOT) on the day of enrollment.  If one or both RDTs are positive, she will receive a complete curative dose of ACT or quinine according to the national policy for treatment of parasitemia in pregnancy; the initial dose will be given by DOT, and the remaining doses will be given to the participant with clear instructions for completing the regimen at home.  SP or ACT/quinine will be administered by health center clinical staff.  If a patient receives curative treatment with ACT or quinine a return visit (or home visit by study staff) to administer a dose of SP will be scheduled for 2 weeks after the end of the curative treatment.  The rationale for use of both curative treatment and SP-IPTp for RDT-positive women is as follows:  The mode of action of IPTp is not yet completely understood, but is presumed to be two-fold: an IPTp dose treats existing asymptomatic infections, and also prophylaxes against new infections due to the long half-life of the drug used (eg SP).  There is some evidence that the primary benefit may result from “intermittent suppressive chemoprophylaxis.” ^50^ Quinine does not have such a prophylactic effect, and while ACTs pair a short-acting artemisinin compound with a longer-acting drug, there is as yet no evidence that ACTs provide a prophylactic effect similar to SP in pregnancy.  Therefore, we believe that the safest approach for the current study is to provide both curative treatment, and the currently recommended IPTp drug, ie SP.  While there are no published data that raise concern for clinically significant drug interactions among these antimalarials, for reasons of tolerability and due to the limited data on pharmacokinetics in pregnant populations we will administer the drugs 2 weeks La participante recevra une dose complète de SP à l’inclusion (dose directement observée) au cas où les deux tests de diagnostic rapides (HRP2 et pLDH) sont négatifs. Elle recevra un traitement à base de quinine ou à base d’artemisinine curatif à base de quinine (selon la politique nationale de traitement du paludisme simple chez la femme enceinte dans le pays) si au moins un des tests est positif. Le reste de la dose curative sera remise à la participante pour prise à domicile avec des instructions claires pour les prises. Si un patient reçoit un traitement curatif, une visite à domicile sera prévue pour administrer la dose de TPI à base de SP dans un délai de 14 jours à compter de la date de la dernière dose curative.

La justification du TPI deux semaines après le traitement curatif chez les femmes ayant un test de diagnostic rapide du paludisme positif est : Le mode d'action du TPI n'est pas encore complètement élucidé, mais semble etre lié à deux mécanismes : le traitement des infections existantes au moment de la dose de TPI mais aussi la prévention de nouvelles infections (effet prophylactique due à la longue demi-vie de la SP) et selon certaines études, le principal avantage serait une prophylaxie intermittente et suppressive. ^50^ La quinine et les traitements à base d’artemisinine n’offrent pas un effet prophylactique similaire à la SP pendant la grossesse. Par conséquent, nous estimons que la meilleure approche pour les participantes de la présente étude est de fournir à la fois un traitement curatif suivi d’une dose de TPI deux semaines plus tard. Bien qu'il n'existe pas de données publiées qui suscitent des préoccupations pour des interactions médicamenteuses cliniquement significatives entre ces antipaludiques, l’administration du TPI deux semaines après la dose curative permettra d’ameliorer la tolérabilité du traitement.

7.3.4 Provision of symptomatic and routine antenatal care

*7.3.4 Consultation prénatales de routine et prise en charge des participantes*

Le premier contact de toute femme enceinte sera le service de consultation prénatale ou elle est recue par le personnel du centre ; des informations seront recueillies sur les antécédents médicaux, chirurgicaux, gynéco-obstétriques et sur la grossesse actuelle de chaque femme ainsi que tout traitement récent. Une brève évaluation clinique sera effectuée par le personnel sage-femme ou infirmier du centre. Chaque participante bénéficiera de la CPN de routine y compris les suppléments de fer et acide folique, des conseils nutritionnels et une moustiquaire imprégnée à longue durée d’action. Les informations sur la provision et l’utilisation de ces outils seront seront enrégistrées à l’inclusion et durant les visites ultérieures. A la dernière étape de la consultation qui est l’administration du TPI-SP, la femme sera référée au personnel du projet pour la réalisation des activités inscrites dans le présent protocole : interrogatoire, examen clinique, prélèvement veineux et traitement (quinine/artemisinine ou TPI-SP).

Tout autre acte médical en dehors de ceux spécifiés dans le présent protocole est du ressort du personnel du centre. Le centre de santé de Colsama est une formation sanitaire publique offrant des soins gratuits de qualité conforme aux normes nationales et à moindre cout. Tout problème médical dépassant la compétence du centre sera référé à un échellon supérieur. Comme sera clairement decrit pendant le processus de consentement, la prise en charge médicale et eventuellement la référence relève en premier lieu du personnel du centre; le projet et son personnel ne pourront pas assister financièrement.

*7.3.5 Suivi des patientes et planification des visites ultérieures*Les participantes seront invitées à revenir sur le site d'étude pour leur prochaine visite prénatale de routine et, si possible, pour toutes autres consultations. Les visites ultérieures seront conformes à la norme nationale de soins qui comprend généralement au moins une visite de CPN aux deuxième et troisième trimestres de la grossesse. Des visites de routine seront prévues au moins 4 semaines d’intervalle ; selon les directives le delai entre deux prises de SP est de 4 semaines au minimun. Chaque participante recevra une carte / livret avec le calendrier des prochaines visites ; dans ce carnet sera consignés toute l’histoire médicale et l’itinéraire therapeutique de la participante, les dates de futures visites d'étude, et dans laquelle d'enregistrer tous les soins ou un traitement qui est reçu par les fournisseurs de site non-étude entre les visites de routine.

**7.4 Activités au cours des visites prénatales** **ultérieures**

*7.4.1 Clinical data collection7.4.1 Collecte de données cliniques*

At each subsequent scheduled antenatal visit, information will be collected from the participant on any treatments sought since the previous visit and on current symptoms, and a brief clinical assessment will be performed by a health center staff clinician.

At the time of enrollment, information will be collected from each participant on her medical and pregnancy history, and a brief clinical assessment will be performed by a health center staff clinician (see sample data collection form, Appendix D)Lors des visites ultérieures, des informations seront recueillies auprès de chaque participante sur ses antécédents médicaux, chirurgicaux, gynéco-obstétriques sur la grossesse actuelle et sur toute prise médicamenteuse récente. Une brève évaluation clinique sera effectuée par le personnel sage-femme ou infirmier du projet après qu’elle ait eu sa CPN de routine (voir formulaire de collecte de données, Annexe D).

*7.4.2 Laboratory sample collection*

*7.4.2 Collecte d'échantillons de laboratoire*

Study staff will collect blood from participants for use in study laboratory procedures, and will prepare the samples and conduct the diagnostic tests described; all other care will be provided by health center staff (ie clinicians, typically midwives and nurses, employed by the health center).  At each scheduled visit, the participant will provide a 2 mL (two milliliter) venous blood sample for HemoCue Hb measurement, preparation of two RDTs, preparation of thick and thin smears for light microscopy, and storage in a microtainer and on filter paper for PCR (see further details in section 7.6 below).  Hb and RDT results will be used in clinical management as in sections 7.4.3 and 7.6.1 below; microscopy and PCR results will not be immediately available and will not be used in management. Le personnel de l'étude prélevera le sang de la participante pour la réalisation des examens de laboratoire et tests prévus dans le présent protocole ; tout autre prélèvement en dehors de ceux spécifiés dans le présent protocole sera réalisé par le personnel du centre. Chaque participante devra fournir deux mililitres (2 ml) de sang veineux dans un tube EDTA pour la mesure du taux d’hémoglobine avec un HemoCue (hémoglobine Hb), la préparation de deux TDRs, la préparation d’une goutte épaisse / frottis mince pour la microscopie optique, la réalisation d’un confettis (2-3 gouttes de sang sur du papier Whatman) et le stockage du culot dans un Microtainer et pour la réalisation de la PCR (voir détails dans la section 7.6 ci-dessous). Le taux d’hémoglobine et les résultats des TDRs seront utilisés pour la prise en charge des patientes ; le résultat de la microscopie et les résultats de la PCR ne seront pas immédiatement disponibles et ne seront pas utilisés dans la prise en charge de la patiente.

*7.4.3 Administration des médicaments antipaludiques*

If both RDT results are negative, the participant will receive a complete dose of SP by directly observed therapy (DOT) on the day of enrollment.  If one or both RDTs are positive, she will receive a complete curative dose of ACT or quinine according to the national policy for treatment of parasitemia in pregnancy; the initial dose will be given by DOT, and the remaining doses will be given to the participant with clear instructions for completing the regimen at home.  SP or ACT/quinine will be administered by health center clinical staff.  If a patient receives curative treatment with ACT or quinine a return visit (or home visit by study staff) to administer a dose of SP will be scheduled for 2 weeks after the end of the curative treatment.  The rationale for use of both curative treatment and SP-IPTp for RDT-positive women is as follows:  The mode of action of IPTp is not yet completely understood, but is presumed to be two-fold: an IPTp dose treats existing asymptomatic infections, and also prophylaxes against new infections due to the long half-life of the drug used (eg SP).  There is some evidence that the primary benefit may result from “intermittent suppressive chemoprophylaxis.” ^50^ Quinine does not have such a prophylactic effect, and while ACTs pair a short-acting artemisinin compound with a longer-acting drug, there is as yet no evidence that ACTs provide a prophylactic effect similar to SP in pregnancy.  Therefore, we believe that the safest approach for the current study is to provide both curative treatment, and the currently recommended IPTp drug, ie SP.  While there are no published data that raise concern for clinically significant drug interactions among these antimalarials, for reasons of tolerability and due to the limited data on pharmacokinetics in pregnant populations we will administer the drugs 2 weeks La participante recevra une dose complète de SP à l’inclusion (dose directement observée) au cas où les deux tests de diagnostic rapides (HRP2 et pLDH) sont négatifs. Elle recevra un traitement curatif à base de quinine ou d’artemisinine selon la politique nationale de traitement du paludisme simple chez la femme enceinte dans le pays si au moins un des tests est positif. Le reste de la dose curative sera remise à la participante pour prise à domicile avec des instructions claires pour les prises. Si un patient reçoit un traitement curatif, une visite à domicile sera prévue pour administrer la dose de TPI à base de SP dans un délai de 14 jours à compter de la date de la dernière dose curative. (Voir la section 7.3.3 pour la justification.)

*7.4.4 Suivi des patientes et planification des visites ultérieures*

Pour toute visite ultérieure, le service de consultation prénatale sera le premier contact de toute femme enceinte y comprise celles participant à la présente étude. Tous les soins standard de la consultation prénatale de routine seront offerts le personnel du centre. Les participantes au projet seront munies d’une carte d’identification et seront référées au personnel du projet juste avant l’étape d’administration du TPI-SP. La participante au moment de la visite de consultation prénatale ultérieure sera prise en charge selon les normes locales de soins par le personnel du centre de santé, comme dans la section 7.3.4. Des informations seront collectées sur les symptômes developpés par la participante ainsi que les traitements recus. Tout probleme médical dépassant la compétence du centre sera référé à un échellon supérieur selon la pratique en vigueur, comme dans la section 7.3.4.

**7.5 Study activities at the time of delivery** **7.5 Activités de l'étude pendant l’accouchement**

At delivery, study staff will collect a 2 mL (two milliliter) blood sample from the participating mother by venipuncture, and from the placenta by standard methods. Maternal peripheral blood will be measured for Hb by Hemocue; both maternal and placental blood will be used to prepare RDTs and blood smears, and for storage in microtainers and on filter paper for PCR (see further details in section 7.6).  In addition, placental tissue samples will be processed for histopathological examination using standardized methods.  Infant birth weight will be obtained using a calibrated scale. A l’accouchement, le personnel de l’étude prélevera deux mililitres (2 ml) de sang veineux de la mère participante et deux mililitres (2 ml) du sang placentaire seront également prélevés par les méthodes standard. Du sang périphérique maternel sera prélevé pour la mesure du taux d’hémoglobine avec un HemoCue; le sang maternel et placentaire sera utilisé à la fois pour préparer les TDRs et les gouttes épaisses / frottis mince, et pour le stockage dans les microtainers et sur du papier filtre (buvard) pour les analyses PCR (voir détails dans la section 7.6). En outre, des échantillons de tissu placentaire seront prélevés et traités pour un examen histopathologique selon des méthodes standardisées. Le poids du nouveau-né à la naissance sera obtenu à l'aide d’une balance graduée. If a participant does not deliver at the study site, study staff will attempt to visit her to obtain data and samples within 24 hours of the birth.  Data on date, time and type of birth, any complications, maternal Hb and infant weight, and the date and time of collection of blood and placental samples and outcome data will be recorded. Si pour une raison quelconque, une participante n’accouche pas à la maternité du centre abritant l’étude, l’équipe s’efforcera de rentrer en contact avec elle pour obtenir les échantillons décrits plus haut dans un délai de 24 heures. La date et l’heure ainsi que le type d’accouchement seront enrégistrées ; la date et l’heure de réalisation de tous les prélevements seront enrégistrées ainsi que l’identité de la personne ayant assuré les dits prélevements. Enfin l’hémoglobine maternelle et le poids du nouveau-né seront enrégistrés ainsi que toute complication survenue au cours de l’accouchement. Testing of mothers for malaria at delivery is not routine, and there is not yet an established standard of care for management of babies born to women testing positive under these circumstances.  Congenital malaria remains a poorly understood occurrence. ^51, 52^ However, some evidence suggests that risk of congenital malaria is higher for infants of HIV-positive women ^53^ (note that this study will not enroll HIV-positive women), and for infants of primigravidae. ^54^ Notably, an association between placental infection and clinical malaria in the infant is not yet well established. ^54^ For this study, if maternal peripheral blood or placental blood is positive at delivery by RDT, study staff will inform the managing clinician and the mother of the result, and will recommend that the infant be observed for symptoms; if symptoms occur, the infant should be tested (if possible according to local standards of care) and treated for malaria according to national guidelines. In addition, when microscopy results become available, if maternal peripheral or placental blood smears are positive, study staff will notify the mother by phone or home visit.  As on previous study visits, if in the judgment of health center clinical staff a participating mother or her infant requires referral for medical reasons, this will be done according to usual health center procedures as in section 7.3.4.

Le test des mères à l'accouchement pour le diagnostic du paludisme n'est pas une pratique de routine, et il n'y a pas encore de norme établie pour la prise en charge des bébés nés des femmes positives dans ces circonstances. Le paludisme congénital reste un événement mal compris. ^51, 52^ Cependant, certaines données suggèrent que le risque de paludisme congénital est plus élevé pour les nourrissons de mères VIH-positives ^53^ (noter que cette étude n’incluera pas des femmes VIH positive), et pour les enfants de primigestes. ^54^ Notamment, une association entre l'infection placentaire et le paludisme clinique chez l'enfant n’est pas encore bien établie. ^54^ Pour cette étude, si le sang périphérique de la mère ou le sang placentaire est positif (à l’HRP2 ou le pLDH) à l’accouchement, le clinicien et la mère devront etre informés du résultat ; il est alors recommandé de mettre en observation le nouveau-né. Si des symptômes apparaissent, l'enfant doit être testé (si possible selon les normes locales de soins) et traités contre le paludisme conformément aux directives nationales. En outre, lorsque les résultats de la microscopie seront disponibles et que le sang maternel ou placentaire sont positifs le personnel de l'étude en informera la mère par téléphone ou par visite à domicile. Comme pour les visites d'étude précédente, si une mère ou son enfant doit etre référé à un échellon supérieur, cela se fera selon les procédures habituelles du centre de santé comme dans la section 7.3. 4.

Il est reconnu que les résultats défavorables de l'accouchement peut conduire à des émotions négatives et stressantes pour la femme, ses proches et même pour le personnel du centre. Dans le cas malheureux d'un résultat négatif de naissance (par exemple mort du nouveau-né) le personnel de l'étude s’efforcera de recueillir des données pertinentes en temps opportun. Ce recueil doit etre respectueux de la dignité de la femme, adapté au contexte, et doit présenter le moins d'inconvénients possible à la femme et sa famille. La discussion de ces idées, les scénarios possibles et les moyens appropriés seront organisées avec le personnel d'étude et le personnel du centre de santé pendant la formation avant le début de l’étude et sera basé dans la mesure du possible sur les directives en vigueur. ^55, 56^

**7.6 Spécifiques évaluations cliniques et de laboratoire**

*7.6.1 Measurement of hemoglobin (Hb) and assessment of anemia7.6.1 Mesure de l'hémoglobine (Hb) et de l'évaluation de l'anémie*

At the time of each routine antenatal visit, and again at delivery, maternal blood will be used to measure hemoglobin with a HemoCue machine (Quest Diagnostics, Ängelholm, Sweden). Based on WHO definitions, a participant with Hb < 11 g/dL will be considered to be anemic, and Hb < 7 g/dL will be considered to be severe anemia.  Anemia will be managed by health center clinical staff according to national guidelines for anemia in pregnancy, which may include additional iron and/or folate supplementation, and/or administration of an anti-helminthic.  Data on provision of such care will be recorded (as in section 7.4 above). A chaque visite prénatale de routine et à l’accouchement, le sang maternel sera utilisé pour mesurer le taux l'hémoglobine avec une machine HemoCue (Quest Diagnostics, Ängelholm, Suède). Sur la base de définitions de l'OMS, toute participante dont le taux d’hémoglobine est inférieur à 11 g / dL sera considéré comme anémiée, et Hb <7 g / dL sera considéré comme une anémie sévère. L’anémie sera pris en charge par le personnel du centre de santé conformément aux directives nationales pour le traitement de l'anémie pendant la grossesse, ce qui peut inclure une supplémentation en fer acide folique, et / ou l'administration d'un anti-helminthique. Toutes ces données seront enregistrées (comme dans la section 7.4 ci-dessus).

*7.6.2 Rapid diagnostic tests (RDTs)7.6.2 Tests de diagnostic rapide (TDR)*

This study will evaluate the three most commonly used antigen-detection systems in RDTs: *Plasmodium falciparum-* specific plasmodium lactate dehydrogenase (Pf-pLDH), pan-specific plasmodium lactate dehydrogenase (pan-pLDH), and histidine-rich protein 2 (HRP2).  According to current test format availability, in order to obtain reliable results for HRP2, pan-pLDH and Pf-pLDH or this study, two different combination RDTs (ie each with two test lines detecting different antigens) will be used.  RDTs will be obtained directly from their manufacturers, and will be centrally procured. For each of the two types of RDT to be evaluated, all test kits used in the study will be from a single lot.  Batch numbers, expiry dates, date received, and date opened will be recorded for each package of RDTs.  All study personnel will receive hands-on training in preparation and interpretation of the RDTs before the study begins.Cette étude permettra d'évaluer les trois systemes de détection de l'antigène les plus couramment utilisés dans les TDR: *Plasmodium falciparum*-spécifique plasmodium lactate déshydrogénase (Pf-pLDH), pan-spécifique de la plasmodium lactate déshydrogénase (pan-pLDH), et riches en protéines histidine 2 (HRP2). Selon le format des tests disponibles afin d'obtenir des résultats fiables pour HRP2, pan-pLDH et Pf-pLDH, au cours de cette étude, deux differentes combinaisons de TDR (cest-à-dire chacun avec deux lignes de détection de différents antigènes) seront utilisées. Les TDR seront obenus directement chez le fabricant, et seront gérés au niveau central. Pour chacun des deux types de TDR à évaluer, tous les kits de test utilisés dans l'étude seront d'un seul lot ; le numéro de lot, les dates d'expiration, la date de réception, et la date d'ouverture seront enregistrés pour chaque paquet de TDR.

*7.6.2.1 Quality control and storage of RDTs7.6.2.1 Contrôle de la qualité et le stockage des TDR*

Each lot of RDTs will undergo lot testing according to WHO guidelines ^57^ before and at the end of the study.  RDT testing will take place in the laboratory of Dr John Barnwell at the Centers for Disease Control and Prevention (CDC), Atlanta, Georgia, USA.  Prior to the start of the study activities at each site, the RDTs to be evaluated will be stored in their original packaging at room temperature in a central storage space.  Stocks of RDTs, adequate to complete training and the study at each site, will be transported to each health center prior to the training and pilot period at each site.  Manufacturers recommend that RDTs be stored between 4 and 40° C.  Temperature and humidity of the storage areas will not be controlled, but will be monitored and recorded regularly to document storage conditions at each site. Chaque lot de TDR sera testé en lot conformément aux directives de l'OMS ^57^ avant et à la fin de l'étude. Le test des TDR aura lieu dans le laboratoire du Dr John Barnwell dans les Centers for Disease Control and Prevention (CDC), Atlanta, Géorgie, États-Unis. Avant le début des activités de l'étude de chaque site, les TDR seront évalués et stockés dans leur emballage d'origine à température ambiante dans un espace de stockage central (IRSS-DRO pour le site burkinabé). Un stock suffisant de TDR de déploiement rapide, adéquate pour compléter la formation et l'étude de chaque site, seront transportés à chaque centre de santé avant la période de formation et l’étude pilote sur chaque site. Les fabricants recommandent que les TDRs soient conservés entre 4 et 40 ° C. La température et l'humidité des zones de stockage ne seront pas contrôlés, mais ils seront surveillés et enregistrés régulièrement afin de documenter les conditions de stockage sur chaque site.

*7.6.2.2 RDTs to be evaluated7.6.2.2 TDR à évaluer*

The following criteria will be considered in selecting the RDTs for this evaluation: high performance in WHO/FIND standardized product testing, ^27^ ease of use, safety (minimal exposure to blood during test preparation), completeness of packaging, appropriate packaging for transport and storage in tropical environments (each test individually wrapped in foil with plastic liner), reasonable market price, and reliability of the supply of the RDTs. Les critères suivants seront pris en compte dans la sélection des TDR pour la présente étude:

- La haute performance sur la base des tests normalisés de l'OMS, ^27^

- La facilité d'utilisation,

- La sécurité (exposition minimum au sang lors de la préparation),

- L'intégralité de l'emballage, un emballage approprié pour le transport et le stockage en milieu tropical (chaque test enveloppé individuellement dans du papier d'aluminium avec revêtement en plastique),

- Le prix de marché raisonnable, et la fiabilité de l'approvisionnement du TDR.

*7.6.2.3 Preparation of RDTs7.6.2.3 Préparation des TDR*

RDTs will be performed on maternal blood obtained by venipuncture at the time of each routine antenatal visit, and at the time of delivery.  In addition, RDTs will be performed on placental blood obtained at delivery.  Individual RDTs will be labeled with the participant's unique study number and initials. A member of the study staff will prepare the RDTs according to manufacturer instructions.  All personnel will use universal precautions when handling blood samples. Les TDR seront effectués sur le sang maternel obtenu par ponction veineuse au cours de chaque visite prénatale de routine, et au moment de l’accouchement. En outre, les tests seront effectués sur le sang placentaire obtenus lors de l'accouchement. Les TDRs seront identifiés avec le numéro unique de suivi et des initiales de la participante. Un membre du personnel de l'étude s’occupera de préparer les TDRs selon les instructions du fabricant. Tout le personnel utilisera les précautions universelles lors de la manipulation des échantillons de sang.

*7.6.2.4 Interpretation and recording of RDT results7.6.2.4 Interprétation et enregistrement des résultats des TDRs*

Each RDT will be interpreted by study personnel who are not responsible for clinical management decisions. At the end of the manufacturer-recommended development time for each test (typically 15 minutes), the RDT result will be recorded on the patient's data form and will be given to the clinician caring for the participant.  An RDT result will be considered positive if both the control line and the test line are visible after the development time.  A result will be considered negative if the control line is visible, but no test line appears.  The result will be considered invalid if no control line is visible, regardless of whether or not a test line appears.  If an RDT result is invalid, the test will be repeated using the same sample of venous blood. Chaque TDR sera interprété par le personnel de l'étude qui n’est pas responsable de la prise en charge clinique des participantes. A la fin du temps requis par le fabricant pour la lecture (en général 15 minutes), les résultats du TDR seront enregistrées sur le formulaire de données de la patiente (voir les annexes) et sera mis à la disposition du personnel chargé de la prise en charge clinique des participantes. Un résultat de TDR sera considéré comme positif si la ligne du contrôle et la ligne de l’échantillon à tester sont visibles après le temps de développement. Un résultat sera considéré comme négatif si la ligne de contrôle est visible, mais aucune ligne de test n’apparaît. Le résultat sera considéré comme invalide si aucune ligne de contrôle est visible, indépendamment de savoir si ou non une ligne de test apparaît. Si un résultat TDR n'est pas valide, le test sera répété en utilisant le même échantillon de sang veineux.

*7.6.3 Microscopy7.6.3 Microscopie*

*7.6.3.1  Préparation et la lecture des gouttes épaisses et frottis sanguins*

At the time blood is obtained for preparation of RDTs, at each routine antenatal visit and at delivery, a thick and thin blood smear also will be prepared.  In addition, at the time of delivery, an impression smear will be prepared from placental blood according to standardized methods.  Blood smears will be stained with Giemsa, fixed, and transported to a central laboratory at each participating site for expert reading.  Microscopists reading blood smears will be blinded to the RDT results.  Thick blood smears will be evaluated for the presence of parasitemia (asexual forms) and gametocytes.  Parasite densities are calculated by counting the number of asexual parasites per 200 leukocytes (or per 500, if the count is <10 asexual parasites/200 leukocytes), assuming a leukocyte count of 8,000/ m l. A blood smear will be considered negative when the examination of 100 high power fields does not reveal asexual parasites or gametocytes. If a thick smear is positive, the corresponding thin blood smear will be evaluated for parasite species. Au cours de chaque visite prénatale de routine et à l’accouchement, du sang veineux sera collecté pour la réalisation des TDRs, la goutte épaisse/frottis mince. En outre, au moment de l’accouchement, une goutte épaisse / un frottis mince sera préparé à partir de sang placentaire selon des méthodes standardisées. Goutte épaisse / frottis sanguins seront colorés au Giemsa, fixé, et transportés dans un laboratoire central sur chaque site participant pour la lecture par un microscopiste. Les microscopistes chargés de la lecture des gouttes épaisses / frottis sanguins ne connaîtront pas les résultats des TDRs. Les gouttes épaisses seront évaluées pour la présence de formes asexuées et de formes gamétocytes. Les densités parasitaires sont calculées en comptant le nombre de parasites asexués par 200 leucocytes (ou par 500, si le nombre est <10 asexuée parasites/200 leucocytes), en supposant un nombre de leucocytes de 8.000 / ml de sang. Un frottis de sang sera considéré comme négatif lorsque l'examen de 100 champs de forte puissance ne révèle pas de parasites asexués ou gamétocytes. Si une goutte épaisse est positive, le frottis sanguin mince correspondant sera évalué pour l’identification des espèces plasmodiales.

*7.6.3.2 Quality control of microscopy results7.6.3.2 Contrôle de la qualité des résultats de la microscopie*

Before the commencement of field work, designated microscopists at each site will be pre-qualified through blinded readings on slides prepared according to the WHO slide bank protocol, and required to reach level 1 or 2 expertise level (as described in the WHO Malaria Microscopy Quality Assurance Manual, version 1, Chapter 5). ^58^ All slides collected during the study at each site will be read by two microscopists.  Slides with discrepant results will be re-read by a third external reader (Level 1 WHO microscopist) and returned to the study site after reading.Avant le début des travaux de terrain, les microscopistes désignés sur chaque site seront pré-qualifiés à travers des lectures aveugles des lames préparées selon la banque de lames de l'OMS, et il est nécessaire pour etre qualifié d’atteindre le niveau 1 ou 2 niveau d'expertise (comme décrit dans le Manuel d’assurance de la qualite de microscopie de l’OMS, version 1, chapitre 5). ^58^ Tous les lames recueillies au cours de l'étude sur chaque site seront lues par deux microscopistes ; les lames qui auront des résultats discordants seront relues par un lecteur externe (niveau 1 des microscopistes) et renvoyés au site d'étude après la lecture. In addition, 10% of slides from each site will be sent for review at an internationally recognized center of excellence for malaria microscopy.  All microscopists will be blinded to RDT results and to results of previous microscopy. En outre, 10% des lames positives à partir de chaque site seront envoyés pour examen à un centre internationalement reconnu d'excellence pour la microscopie du paludisme. Tous les microscopistes ne connaîtront pas les résultats de TDR ou les résultats de la microscopie précédente.

*7.6.4 PCR for detection and speciation of parasitemia* *7.6.4 PCR pour la détection et la spéciation des parasites*

As for RDT and blood smear preparation, at the time of each routine antenatal visit and at delivery, two drops of maternal blood will be collected onto filter paper and 250uL into a microtainer containing EDTA anticoagulant.  In addition, at the time of delivery, placental blood will be collected onto filter paper and into EDTA microtainer/s.  Each filter paper sample will be labeled with the participant's study number, air-dried, and stored in a sealed plastic bag at ambient temperature.  Microtainers will be labeled with the participant's study number and stored at -20°C. Samples will be transferred to a central laboratory and analyzed by PCR to confirm presence or absence of parasitemia, and parasite species. PCR will be performed at the laboratory of Prof Jean-Bosco Ouedraogo in Bobo-Dioulasso, Burkina Faso, and quality assurance of results will be conducted at the laboratory of Dr John Barnwell at the Centers for Disease Control and Prevention, Atlanta, Georgia, USA. Le TDR du paludisme ainsi qu’une goutte épaisse / frottis mince seront réalisés au moment de chaque visite prénatale de routine et à l’accouchement ; deux gouttes de sang de la mère seront recueillis sur papier filtre et deux mililitres de sang maternel recueillidans un Microtainer contenant un anticoagulant EDTA. En outre, au moment de l’accouchement, du sang placentaire sera prélevé sur du papier filtre et dans un Microtainer contenant de l’EDTA. Chaque échantillon de papier filtre sera étiqueté avec le numéro d'étude de la participante ; il sera séché à l'air, et stocké dans un sac en plastique scellé à la température ambiante. Les microtainers seront étiquetés avec le numéro unique des participantes et stockés -20°C. Les échantillons seront transférés dans le laboratoire central et analysés par PCR pour confirmer la présence ou l'absence des parasites et pour l’identification des espèces plasmodiale. La PCR sera réalisée au laboratoire du Professeur Jean-Bosco Ouedraogo à Bobo-Dioulasso, Burkina Faso, et l'assurance de la qualité des résultats sera effectuée au laboratoire du Dr John Barnwell dans les Centers for Disease Control and Prevention, Atlanta, Géorgie, Etats-Unis.

*7.6.5 Estimation of gestational age7.6.5 Estimation de l'âge gestationnel*

At enrollment, duration of gestation and expected delivery date will be estimated by reported last menstrual period (LMP) and by measurement of fundal height, and these data will be recorded on the participant's case record forms.  At subsequent study visits, fundal height will be measured and recorded.Lors la sélection, l’age de la grossesse, la date probable de l’accouchement seront estimés par rapport dernières règles (LMP) et par la mesure de la hauteur utérine, et ces données seront enregistrées sur la fiche de collecte de données de la participante (voir les annexes). Lors des visites d'études ultérieures, la hauteur utérine sera mesurée et enregistrée.

*7.6.6 Measurement of infant birth weight7.6.6 Mesure du poids à la naissance*

Within 24 hours of delivery, study staff will weigh the infant on a calibrated scale.  Birth weight will be recorded in grams; based on WHO definitions, low birth weight (LBW) will be considered < 2500 g.Dans les 24 heures suivant l'accouchement, le personnel de l'étude évaluera l'enfant sur une échelle étalonnée. Le poids à la naissance sera mesuré en grammes et enregistré sur la fiche de collecte des données de la patiente. Un poids de naissance < 2500 g sera considéré comme un faible poids de naissance selon la définition de l’OMS.

*7.6.7 Placental histopathology for detection of evidence of prior or current placental malaria 7.6.7 Histopathologie placentaire pour la recherche d’infection ancienne et présente*

At the time of delivery, placental blood and tissue sample/s will be collected and processed according to standard methods for smears, storage for molecular analysis, and for histopathological examination.  Specific details of placental collection will be described in more detail in site-specific protocols; in general, for births taking place at the health center, health center staff will assist with delivery, including delivery of the placenta, and will then work with study staff to ensure the placenta is transferred to the laboratory area for processing as efficiently as possible to avoid degradation of the samples.  As in section 7.5, if a participant does not deliver at the study site, study staff will attempt to visit her to obtain data and samples within 24 hours of the birth.  The placenta will be wiped and washed with approximately one liter of phosphate buffered solution (PBS) to remove blood clots, placed umbilical cord side down on a clean, dry cloth, and gently blotted with a paper towel. Placental blood samples and biopsies will be collected and stored for further staining and processing.  Placental histology samples will be read by two expert microscopists with discrepant results resolved by a third reader, with a proportion of samples sent to an independent research group for external quality control. Au moment de l’accouchement, le sang placentaire et un échantillon de tissu placentaire seront collectés et traités selon les méthodes standards pour les frottis, le stockage pour l'analyse moléculaire, et pour un examen histopathologique. Les détails précis de la collecte du placenta seront décrits selon le site. En général, pour les naissances qui ont lieu au centre de santé, le personnel du centre de santé collabore à la prestation, y compris la délivrance du placenta, et ensuite travailler avec le personnel d'étude pour s'assurer que le placenta est transféré vers une zone du laboratoire pour un traitement efficient évitant toute dégradation des échantillons. Comme dans la section 7.5, si une participante n’accouche pas au centre de santé abritant l’équipe de l’étude, celle-ci s’efforcera de visiter la participante dans un délai de 24 heures. Le placenta sera lavé avec environ un litre de solution tamponnée au phosphate (PBS) pour enlever les caillots sanguins ; le placenta devra etre placées le côté du cordon ombilical vers le bas sur un chiffon propre et sec, et lentement nettoyé avec une serviette en papier. Un échantillon de sang placentaire et des biopsies seront collectées et stockées pour la coloration et le traitement. L’histologie de l’échantillon placentaire sera lue par deux microscopistes expert. Les résultats discordants seront résolus par un troisième lecteur, avec une partie des échantillons envoyés à un groupe de recherche indépendant pour le contrôle de qualité externe.

*7.6.8 Collection of data on other maternal and fetal outcomes* *7.6.8 Collecte de données sur d'autres résultats maternels et fœtaux*

In addition to the specific assessments described above, data on other significant maternal and fetal outcomes will be collected.  In particular, the study staff will endeavor to collect data on management of any febrile or afebrile illnesses during pregnancy, and on birth outcomes including requirement and rationale for Cesarean section, any obstetrical complications, and fetal or maternal death.  If a participant does not return to the health center at the anticipated time of delivery, study staff will contact the participant by phone and/or conduct a home visit to attempt follow-up.  A participant will be considered lost to follow-up if no data can be collected within 7 days of delivery; within the 7-day limit, complete or partial data (eg maternal blood and infant weight, but no placenta) may be collected. En plus des évaluations spécifiques décrites ci-dessus, l’équipe s’efforcera de collecter toute donnée importante sur la mère pendant la grossesse ; ce sont notamment des informations sur la prise en charge de toute pathologie pendant la grossesse (pathologie fébrile ou non), les raisons d’une intervention en cas césarienne, toute complication obstétricale intercurente, toute issue fatale de la mère ou de l’enfant. Si une participante ne retourne pas au centre de santé à l'heure prévue de l'accouchement, le personnel de l'étude prendra immédiatement contact avec la participante par téléphone et / ou une visite à domicile pour s’enquérir de l’état de la participante et l’encourager à venir au centre pour son accouchement. Une participante sera considérée comme perdue pour le suivi en l'absence de données collectées dans les 7 jours suivant l’accouchement; dans ce délai de 7 jours, des informations complète ou partielle (par exemple le sang maternel et le poids de bébé, mais pas de placenta) peuvent être collectées.

**8 SÉCURITÉ DES PARTICIPANTES**

**Potential risks8.1 Risques potentiels**

Risks associated with participation in this evaluation are minimal, essentially no greater than they would be for routine antenatal care in malaria-endemic areas.  All participants will receive standard-of-care antenatal services, including provision of IPTp with SP according to WHO and national recommendations. To the routine evaluation of pregnant women at antenatal visits, this study adds the collection of 2 mL (two milliliters) of venous blood, a negligible medical risk.  Physical discomfort, transient bleeding and bruising may result when blood is obtained by venipuncture. Aseptic technique and universal precautions against body fluid exposures will be practiced in obtaining blood samples.  Some participants will present to the health facility with asymptomatic or symptomatic malaria infection, which is potentially detrimental to both mother and fetus; however, all participants will be managed by qualified clinical staff according to WHO and national standards of care.  While loss of privacy is a potential risk associated with participation in any research project, no sensitive information will be collected, and no social risks are foreseen for participating women.  Potential for loss of confidentiality will be minimized by use of coded study numbers on data collection forms and laboratory samples, rather than participant names, and all data will be stored in locked filing cabinets and password-protected computer files.Les risques associés à la participation à cette évaluation sont minimes, essentiellement pas plus qu'ils ne le seraient pour des soins prénatals de routine dans les régions où le paludisme est endémique. Toutes les participantes recevront des soins dont la qualité est équivalente à ceux dispensés en routine dans le centre de santé : provision de TPI avec SP selon les directives du programme national de lutte contre le paludisme et l'OMS. En plus de l'évaluation de routine des femmes enceintes lors des visites prénatales, deux millilitres (2 ml) de sang veineux seront recueillis. Ce prélevement peut provoquer : un inconfort physique, un saignement transitoire, et des ecchymoses. Notre équipe est expérimentée dans ce type de prélevement et avec une aseptie rigoureuse du point de prélevement et mettra en œuvre les précautions universelles contre les expositions aux liquides biologiques. Certaines participantes présentant au centre de santé seront malades ou parasitées (symptomatiques ou assymptomatiques) ce qui est potentiellement préjudiciable pour la mère et le fœtus, mais tous les participantes seront gérées par un personnel qualifié selon les normes de l'OMS et les normes nationales de soins. Bien que la perte de la confidentialité soit un risque potentiel associé à la participation à tout projet de recherche, aucune information sensible ne sera recueillie, et aucune charge ou risque social n’est associée à la participation des femmes à ce projet. Les risques de perte de confidentialité seront minimisés par l'utilisation de codes sur des formulaires de collecte de données et des échantillons de laboratoire, plutôt que les noms des participantes, et toutes les données seront stockées dans des classeurs et armoires verrouillées. Les fichiers informatiques seront protégés par des mots de passe.

**8.2 Known potential benefits8.2 Avantages potentiels pour les participantes**

All participating women will be provided with basic antenatal services according to the local standard of care. Participants may also benefit from the knowledge that they are assisting in the evaluation of potentially valuable methods for prevention and control of malaria for pregnant women in endemic areas.Toutes les femmes participantes bénéficieront de consultation prénatale selon les normes de qualité des soins en vigueur. Le traitement des cas de paludisme sera gratuit. En participant à cette évaluation, les participantes contribueront à l’avancée de la recherche et à l'évaluation des méthodes potentiellement utiles pour la prévention et le contrôle du paludisme pour les femmes enceintes dans les zones d’endémie palustres.

**8.3 Data safety and monitoring board (DSMB)8.3 Le comité de contrôle pour la sécurité des données (DSMB)**

To ensure the quality and integrity of data, and the safety of participants, an independent study monitor will be contracted for the duration of the study.  The monitoring group will be identified and engaged following standard WHO/TDR procedure. Pour assurer la qualité et l'intégrité des données et la sécurité des participantes, un moniteur indépendant sera engagé pour la durée de l'étude. Les groupes de moniteurs seront identifiés et engagés selon les normes et procédures de l'OMS / TDR.

**9  FOLLOW-UP 9 Suivi après l’accouchement**

No formal study follow-up is planned for participants after delivery, as described in 7.5.Le suivi des participantes prend fin avec le recueil des informations à l’accouchement et il n’y a pas de plan de suivi des participantes au dela de l’accouchement comme décrit dans la section 7.5.

**10 DATA MANAGEMENT10 GESTION DES DONNEES**

**10.110.1** **Data quality assurance and monitoring** **Assurance de la qualité des données et le suivi**

Le Study staff  and relevant health center staff will be educated in the study protocol prior to the start of study activities at each site.  Data collection forms will be reviewed by the Study Coordinators and Principal Investigators (PIs) for completeness and accuracy.  PIs from the different countries will maintain regular contact to ensure consistency in protocol implementation at each site, and PIs will meet regularly with study staff at their site/s to ensure consistency in the collection of data.personnel de l'étude et le personnel compétent du centre de santé seront formés sur le protocole d'étude avant le début des activités de l’étude sur chaque site. Les formulaires deconsentement et de collecte de données seront examinés par le coordonateur des activités de terrain en appui au principal investigateur pour s’assurer de leur complétude. Les investigateurs principaux des différents pays entretiendront des échanges réguliers afin d'assurer la cohérence dans la mise en œuvre du protocole dans chaque site. Le principal investigateur (PI) organisera régulièrement des réunions avec le personnel de l'étude sur leur site pour assurer la cohérence dans la collecte des données. PIs will participate in regular study group meetings to assess progress of the study, address any difficulties with the RDTs or protocol, and provide feedback to members of the study group.  A two-week pilot period will be conducted before beginning the study at each site, which will allow the PIs and study personnel to identify and resolve potential logistical and technological problems prior to beginning data collection.  Standardized protocols and SOPs will be followed for quality control/quality assurance of specimen procurement and preparation, smear microscopy, placental histology, and PCR. Le principal investigateur participera à des réunions régulières du groupe d'étude pour évaluer les progrès de l'étude et pour trouver des solutions à des difficultés avec les TDRs ou avec le protocole, et fournir un feedback aux membres du groupe d'étude. Une étude pilote de 2 semaines sera initié sur chaque site pour permettre aux principaux investigateurs et aux membres de l’équipe d'identifier et de résoudre les éventuels problèmes logistiques et technologiques avant de commencer la collecte des données. Le protocole normalisé ainsi que les SOPs seront scrupuleusement respectés pour l’assurance / contrôle qualité pour le prélèvement, la préparation des échantillons, la lecture des lames, l'histologie du placenta et la réalisation de la PCR.

**10.210.2** **Records** **Archivages**

All participants will be identified by a unique identifying number throughout the study; participant names will not be recorded or entered into the computerized databases. Data collection forms will be kept in secure files at each study site. All corrections to handwritten information on data collection forms and other study documents will be made by striking through the incorrect entry with a single line and entering the correct information adjacent to it; the correction will be initialed and dated by the study staff member.  For any requested information that is not obtained, an explanation will be noted on the data collection form.  Electronic databases will be stored in password-protected computer files. Access to the records will be limited to study staff.Toutes les participantes seront identifiées par un numéro d'identification unique tout au long de l'étude; les noms des participantes ne seront pas enregistrés ou introduits dans les bases de données informatisées. Les formulaires de collecte de données seront conservés dans des fichiers sécurisés sur chaque site d'étude. Toutes les corrections sur les formulaires de collecte de données et autres documents de l'étude seront faites par la suppression de l'entrée incorrecte avec un seul trait suivi de l’information correcte à côté ; la correction sera datée et signée par le membre du personnel faisant la correction. Pour toute information demandée qui n'est pas disponible, une explication sera indiquée sur le formulaire de collecte de données. Les bases de données électroniques seront stockées dans des fichiers informatiques et protégées par un mot de passe. L'accès aux documents sera limité aux personnels de l'étude mais aussi au moniteur clinique, à l’auditeur, à l’inspecteur, aux membres du comité d’éthique.The investigators and study staff will allow all requested monitoring visits, audits or reviews by relevant ethical review boards.

**10.3 Data management10.3 Gestion des données**

Data will be transferred from the handwritten data collection forms into a computerized database by study personnel and will be double-entered to verify accuracy.  Back-up files of the database will be created after each data entry session.  For quality control, check programs will be written into the databases to limit the entry of incorrect data and ensure entry of data into required fields.  Electronic data records will be maintained at each site and will be transmitted to a centralized database for regular cleaning and management. Les données seront saisies sur une base de données informatisée par le personnel de l'étude et sera à double entrée pour vérifier l'exactitude. Un système de back-up de la base de données sera créé après chaque session de saisie. Pour le contrôle de qualité, les programmes de controle devront être inscrits dans les bases de données pour limiter l'entrée de données erronées et assurer l'entrée des données dans les champs requis. L’enregistrement de données électroniques sera maintenu au niveau de chaque site et sera transmis à une base de données centralisée pour le nettoyage régulier et l’analyse.

**10.4 Immediate and long-term use of the data10.4 Utilisation immédiate et à long terme des données**

Data collected in this study will be compiled, analyzed and made available to collaborating partners, ministries of health in participating countries, the WHO, and will be prepared for publication in a peer-reviewed journal.  Study staff and health center staff will be informed of overall study results after data analysis has been completed.  Upon completion of the study, all study documents and record forms will be filed and stored at the FIND office in Kampala or Geneva for at least 12 years.  Electronic data records will be stored indefinitely by investigator/s in personal computers and external hard drives.  Results of the study may be publicized in the future by collaborating partners and stakeholders as part of public health education efforts and malaria control programs. Les données recueillies dans cette étude seront compilées, analysées et mises à la disposition des partenaires, des collaborateurs, des ministères de la santé des pays participants et l'OMS. Les résultats de la recherche seront valorisés par la soumission d’un manuscrit pour publication dans un journal international. Tout le personnel de l’étude sera informé des résultats globaux à la fin des analyses. À la fin de l'étude, tous les documents d'étude y compris les formulaires de collecte seront déposés et conservés dans les bureaux de FIND à Kampala ou à Genève pendant au moins 12 ans. La base électronique contenant les données sera conservée indéfiniment par les collaborateurs et dans des disques durs externes. Les résultats de l'étude peuvent être publiés dans l'avenir par les partenaires collaborateurs et les parties prenantes dans le cadre des efforts d'éducation du public en matière de santé et de programmes de lutte antipaludique.

**11 STATISTICAL CONSIDERATIONS11 CONSIDERATIONS STATISTIQUES**

Data analysis will be performed by the PIs using Stata statistical software package, with assistance from Michelle Gatton, PhD, of the Queensland Institute of Medical Research and other co-investigators as appropriate.L'analyse des données sera effectuée par les chercheurs principaux à l'aide du progiciel statistique Stata, avec l'aide de Michelle Gatton, PhD, de l'Institut de recherche médicale du Queensland avec l’appui d'autres chercheurs, le cas échéant.

**11.1 Study outcome measures11.1 Résultats l'études**

Results of peripheral blood tests performed during gestation will be analyzed for associations with evidence of placental malaria and pregnancy outcomes.  The statistical analysis will focus on three primary outcomes: 1) negative predictive power of malaria RDTs, peripheral blood films and PCR performed on peripheral blood to diagnose placental malaria during gestation, 2) association of placental malaria during gestation on mean infant birth weight and 3) association of placental malaria during gestation on mean maternal Hb.  These outcomes will be used to assess the potential utility of RDTs as screening tests to identify clinically significant malaria infections at a time when effective preventive measures may be taken. Other factors known to influence the study outcomes, including iron and folate supplementation, bed net use and administration of antihelminthic drugs will be treated as confounders in the analysis.  In addition, stratified analysis will be conducted to assess these associations within subgroups based on geographic location, estimates of malaria endemicity, participant age, and parity (gravida class). Les résultats des tests sanguins périphériques effectués pendant la gestation seront analysés pour déterminer les associations avec les éléments de preuve du paludisme placentaire et l’issue de la grossesse. L'analyse statistique se concentrera sur trois objectifs principaux: 1) la valeur prédicitve négative des TDR du paludisme, de la microscopie du sang périphérique et la PCR effectué sur du sang périphérique pour diagnostiquer le paludisme placentaire pendant la gestation, 2) l'association de paludisme placentaire pendant la gestation avec le poids de naissance moyen du nouveau-né et 3) l'association de paludisme placentaire pendant la gestation et le taux d’hémoglobine maternel. Ces résultats seront utilisés pour évaluer l'utilité potentielle des TDR du paludisme comme des tests de dépistage à meme d’identifier les infections palustre cliniquement significative à un moment où des mesures préventives efficaces peuvent être prises. Les autres facteurs connus pour influencer les résultats de l'étude, y compris les suppléments de fer et de folate, l’utilisation des moustiquaires à longue durée d’action et l'administration de médicaments antihelminthiques seront traités comme des facteurs de confusion dans l'analyse. En outre, l’analyse stratifiée sera réalisée afin d'évaluer ces associations au sein de sous-groupes en fonction du lieu géographique, de l'endémicité du paludisme, l'âge des participantes, et la parité.

**11.2 Analysis plan11.2 Plan d’analyse de l’étude**

*Th* e unit of analysis will be each pregnancy.  For each pregnant woman a complete data set consists of the following:L’unité d'analyse sera chaque grossesse. Pour chaque femme enceinte un ensemble de données complet comprend les éléments suivants:

| **Variable** | **Type de données** | **Classification de la variable** | | | | |
| --- | --- | --- | --- | --- | --- | --- |
|  |  | Confondeur | Témoin du paludisme placentaire | Mesure des résultats | | |
|  |  |  |  | Valeur prédictive du diagnostic | Poids à la naissance | Hb maternelle |
| TDR du sang périphérique pris au cours de 2^ème^ trimestre | Binaire (positif/négatif) |  |  | X |  |  |
| Microscopie du sang pris au cours de 2^ème^ trimestre | 1. Binaire (positif/ négatif)  2. Compter les positifs |  |  | X |  |  |
| PCR du sang pris au cours de 2^ème^ trimestre | Binaire (positif/négatif) |  | X |  |  |  |
| RDT du sang pris au cours de 3^ème^ trimestre | Binaire (positif/négatif) |  |  | X |  |  |
| Microscopie du sang pris au cours de 3^ème^ trimestre | 1. Binaire (positif/ négatif)  2. Compter les positifs |  |  | X |  |  |
| PCR du sang prises au cours de 3^ème^ trimestre | Binaire (positif/négatif) |  | X |  |  |  |
| RDT du sang périphérique prélevé au cours ou peu de temps après l'accouchement | Binaire (positif/négatif) |  |  | X |  |  |
| Microscopie de sang périphérique prélevés au cours ou peu de temps après l'accouchement | 1. Binaire (positif/ négatif)  2. Compter les positifs |  |  | X |  |  |
| PCR du sang périphérique prélevé au cours ou peu de temps après l'accouchement | Binaire (positif/négatif) |  | X |  |  |  |
| Signes histologiques d’infection placentaire | Binaire (positif/négatif) |  | X |  |  |  |
| Résultats du diagnostic des visites supplémentaires à la clinique (par exemple une infection symptomatique) | Binaire (positif/négatif) |  | X |  |  |  |
| Hb maternelle à l'accouchement | Continu |  |  |  |  | X |
| Poids à la naissance du nouveau-né | Continu |  |  |  | X |  |
| Site d’étude | Nominal | X |  |  |  |  |
| Age | Continu | X |  |  |  |  |
| Gravidité | Ordinal | X |  |  |  |  |
| Traitement SP | Binaire (oui/non) | X |  |  |  |  |
| Complément en fer et en acide folique | Binaire (oui/non) | X |  |  |  |  |
| L’utilisation des moustiquaires imprégnées | Binaire (oui/non) | X |  |  |  |  |

Les définitions suivantes seront utilisées:

- Evidence of placental malaria: a positive PCR result is obtained for any peripheral blood sample and / or there is evidence of placental infection by histologyTémoin du paludisme placentaire: un résultat positif de PCR est obtenu pour des échantillons de sang périphérique et / ou il existe des preuves d'infection placentaire par histologie
- RDT positive: a positive RDT result is obtained from one or more blood samplesTDR positif: un résultat positif de TDR est obtenu à partir de sang ou de plusieurs échantillons de sang
- Microscopy positive: parasites are detected by microscopy (thick film) in one or more of the blood sampleMicroscopie positive: les parasites sont détectés par microscopie (goutte épaisse) dans un ou plusieurs des échantillons de sang.

The accuracy of RDTs and peripheral microscopy at detecting placental malaria will be assessed by considering the sensitivity, specificity, positive predictive value and negative predictive value of each diagnostic compared to evidence of placental malaria. La précision des TDR et la microscopie à détecter le paludisme placentaire sera évaluée en tenant compte de la sensibilité, de la spécificité, des valeurs prédictives positive et négative de chaque diagnostic par rapport à l’évidence du paludisme placentaire. This analysis will be conducted for all samples combined and also separately for each study location. Cette analyse sera effectuée pour tous les échantillons combinés et aussi séparément pour chaque site d'étude. Marginal generalized linear models will be developed to assess the impact of confounders on predictive values. Des modèles marginaux linéaires généralisés seront élaborés pour évaluer l'impact des facteurs de confusion sur les valeurs prédictives.

The impact of RDT screening and treatment compared to standard IPTp on infant birth weight and maternal Hb will be assessed for non-inferiority by testing the hypotheses: L'impact du dépistage (par les TDR) suivi de traitement par rapport au traitement TPI standard sur le poids à la naissance du nouveau-né et l’hémoglobine maternelle sera évalué pour l’hypothèse de non-infériorité en testant les hypothèses:

1. Mean birth weight of infants whose mothers were RDT positive & treated with ACT/quinine ≥ mean birth weight of infants whose mothers were RDT negative (and received SP) Poids moyen de naissance des nouveau-nés dont les mères étaient TDR positifs et traités avec ACT/quinine versus poids moyen de naissance des nouveau-nés dont les mères étaient TDR négatif (et reçu TPI-SP)
2. Mean Hb of mothers who were RDT positive & treated with ACT/quinine ≥ mean Hb of mothers who were RDT negative (and received SP) Taux moyen d'hémoglobine de mères qui ont été TDR positive et traités avec ACT /quinine versus taux moyen d'hémoglobine de mères qui ont été TDR négatif (et reçu TPI-SP)

Standard statistical techniques such as t-tests, ANOVA and regression modeling will be used to test these hypotheses and investigate the potential impact of confounders on birth weight and maternal Hb.Les tests statistics standards comme par exemple les t-tests, l’analyse de variance et les régressions seront utilisées pour tester ces hypothèses et d'enquêter sur l'impact potentiel des facteurs de confusion sur le poids à la naissance et l’hémoglobine maternelle.

**11 .3 Sample size considerations** **11.3 Considérations sur la taille de l'échantillon**

The target sample size for the number of participants at each study site is calculated to test the hypothesis Ho: [PPV ≤ (disease prevalence + α) or NPV ≤ (1-disease prevalence)] with 80% power and 5% significance at each study location. La taille requise de l'échantillon pour le nombre de participantes sur chaque site d'étude est calculée pour tester l'hypothèse Ho: [PPV ≤ (la prévalence de la maladie + α) ou VPN ≤ (1-la prévalence de la maladie)] avec une puissance de 80% et un niveau de significance de 5% sur chaque sited'étude. For the higher transmission sites of Uganda and Burkina Faso α=0.4, while α=0.3 for Nigeria.  Previously published estimates of sensitivity (65%) and specificity (98%) were used in these calculations. Pour les sites de transmission plus élevées de l'Ouganda et le Burkina Faso α = 0,4, tandis que α = 0,3 pour le Nigeria. Des estimations de 65% de sensibilité et 98% de spécificité publiées dans la littérature ont servi aux calculs de la taille de l’échantillon.

Using the target sample sizes outlined below the study will be powered to detect changes in infant birth weight and maternal Hb of >85g and 0.34g/dl, respectively.  The sample size for the Burkina Faso site should also allow detection of changes in infant birth weight (>123g) with sufficient power.  Based on published data, other sites will not be individually powered to detect likely changes in birth weight (~123g) and none of the sites will be individually powered to detected likely changes in maternal Hb (~0.4g/dl). However this will not prohibit any of the statistical analysis planned. La taille de l'échantillon décrite ci-dessous permet à l’étude de détecter les changements de poids de naissance du nouveau-né et de l’hémoglobine maternelle > 85 g et 0.34 g/dl, respectivement. La taille de l'échantillon pour le site du Burkina Faso devrait également permettre de détecter des changements à la naissance du poids du nouveau-né de l’ordre de (> 123g) avec une puissance suffisante. Sur la base de données publiées, d'autres sites n’auront pas individuellement la puissance nécessaire pour détecter les changements probables dans les poids de naissance (~ 123g) et aucun des sites n’a une puissance suffisante individuelle pour détecter les changements probables de la moyenne de l’hémoglobine maternelle (~ 0.4g/dl). Cependant toutes les analyses prévues peuvent etre conduites.

We estimate that the statistical requirements of the study can be met by enrolling 345 women at the high-transmission Ugandan site, 460 women at the low-transmission Nigerian site, and 860 women at the seasonal-transmission site in Burkina Faso, for a total sample size of 1,665 participants.  This estimate assumes a 15% loss to follow-up at each study location.Nous estimons que l'inscription de 345 femmes en Ouganda, 460 femmes au Nigeria, et 860 femmes au Burkina Faso, soit un échantillon global de 1.665 participantes, permettra de répondre aux exigences statistiques et d’atteindre les objectifs du projet. Cette estimation suppose une perte de 15% au cours du suivi sur chaque site d'études.

***11* .4  Participant enrollment and follow-up** **11.4 L'inclusion et suivi des participantes**

Recruitment and enrollment will include all eligible pregnant women who present for routine antenatal care in their second or third trimester (sections 6, 7.2.1). Toutes les femmes enceintes qui se présentent pour des soins prénatals de routine dans leur deuxième ou troisième trimestre de la grossesse sont éligibles pour participer à l’étude (sections 6, 7.2.1). Recruitment will continue at each site until the target sample size given in Section 9.3 is reached. Le recrutement se poursuivra sur chaque site jusqu'à ce que la taille de l'échantillon donnée à la section 9.3 soit atteinte.

**12 ASSURANCE DE LA QUALITE**

Quality control and assurance of all diagnostic tests will be conducted according to SOPs and as briefly described in sections 7.6.2.1, 7.6.3.2, 7.6.4 and 7.6.7.  To ensure the quality and integrity of data, and the safety of participants, an independent study monitor will be contracted for the duration of the study as in section 8.3. Le contrôle et l’assurance de la qualité de tous les tests de diagnostic sera mené conformément aux SOP et comme brièvement décrits dans les sections 7.6.2.1, 7.6.3.2, 7.6.4 et 7.6.7. Afin d'assurer la qualité et l'intégrité des données et la sécurité des participantes, un moniteur étude indépendant sera engagé pour la durée de l'étude à la section 8.3.

**13 EXPECTED OUTCOMES OF THE STUDY13 RESULTATS ATTENDUS DE L’ETUDE**

Malaria prevention measures for use during pregnancy, including insecticide-treated nets and IPTp are available and effective.  However, increasing parasite resistance to SP- IPTp raises concerns that this approach will soon lose effectiveness. Screening with rapid diagnostic tests (RDTs) may offer an accurate and practical way to identify pregnant women who will benefit from targeted therapy for placental malaria infection.  This study proposes to assess the efficacy of well-characterized RDTs in detection of placental malaria, to provide data necessary for future evaluations of screening and targeted treatment as a potential alternative to the failing regimen of IPTp with SP.  If RDTs are shown to be accurate in detecting placental malaria, it will provide new evidence to support interventions such as IST (intermittent screening and treatment) in further research and in malaria control policy. Les mesures de prévention du paludisme pendant la grossesse, y compris des moustiquaires imprégnées d'insecticide et le TPI, sont disponibles et efficaces. Cependant, la résistance croissante du parasite à la SP-TPI soulève des inquiétudes que cette approche va bientôt perdre de son efficacité. Le depistage avec les TDR peut offrir une manière précise et pratique afin d'identifier les femmes enceintes qui bénéficieront d'un traitement ciblé de l'infection par le paludisme placentaire. Cette étude propose d'évaluer l'efficacité des TDRs dans la détection du paludisme placentaire, de fournir les données nécessaires pour les évaluations futures de dépistage et de traitement ciblés en tant qu’une alternative potentielle au régime à défaut du TPI à la SP. Si les TDRs sont indiqués pour être précis dans la détection du paludisme placentaire, il fournira de nouvelles preuves à l'appui des interventions telles que TSI (dépistage et le traitement intermittent) en recherche et dans la politique de lutte contre le paludisme.

**14 DISSEMINATION OF RESULTS AND PUBLICATION POLICY14 DIFFUSION DES RESULTATS ET DE LA POLITIQUE DE PUBLICATION**

Data collected in this study will be compiled, analyzed and made available to collaborating partners, ministries of health in participating countries, the WHO, and will be prepared for publication in a peer-reviewed journal.  Study staff and health center staff will be informed of overall study results after data analysis has been completed.  Summarized study results may be posted in the participating health centers for review by participants.  Results of the study may be publicized in the future by collaborating partners and stakeholders as part of public health education efforts and malaria control programs. Les données recueillies dans cette étude seront compilées, analysées et mises à la disposition des partenaires collaborateurs, les ministères de la santé dans les pays participants, l'OMS, et seront préparées pour publication dans un journal revu par des pairs. Le personnel de l'étude et le personnel des centres de santé seront informés des résultats globaux de l'étude à la fin des analyses. Le résumé des résultats de l'étude peut être affiché dans les centres de santé participants aux fins d'information pour les participantes. Les résultats de l'étude peuvent être publiés dans l'avenir par les partenaires collaborateurs et les parties prenantes dans le cadre des efforts d'éducation sanitaire du public et des programmes de contrôle du paludisme.

**15 DURATION OF THE PROJECT15 DUREE DU PROJET**

The duration of participant recruitment and follow-up is expected to take approximately 12 months from the start of study activities at each site, depending on rates of enrollment. La durée du recrutement des participantes et le suivi devrait prendre environ 12 mois après le début des activités de l'étude sur chaque site selon le taux de recrutement.

**Tableau 1. Calendrier proposé de l’étude**

| Activité | Mo 1-3 | | Mo 4-6 | Mo 7-9 | Mo 10-12 | Mo 13-15 | Mo 16-18 |
| --- | --- | --- | --- | --- | --- | --- | --- |
| Prep du site, formation |  | |  |  |  |  |  |
| Pilot, commencer le recrutement |  |  |  |  |  |  |  |
| Inscrire à la taille de l’échantillon |  |  |  |  |  |  |  |
| Suivi des participantes |  | |  |  |  |  |  |
| Contrôle de la qualité des résultats des tests de diagnostic |  | |  |  |  |  |  |
| Achèvement de PCR |  | |  |  |  |  |  |
| Nettoyage des données, l’analyse |  | |  |  |  |  |  |
| Ecriture du rapport |  | |  |  |  |  |  |

**16 PROBLEMES ANTICIPES**

**Tableau 2. Problèmes potentiels de l’étude et des solutions proposées**

| **Problème potentiel** | **Proposed solution** **Solution proposée** |
| --- | --- |
| Insufficient sample size, or slower than expected enrollmentTaille de l'échantillon insuffisant, ou plus lente que prévu d'inscription | 1)  Increase number of clinical sites (within the same geographical/population area) 1) Augmentation du nombre de sites cliniques (dans le même zone géographique / population)  2)  Publicize study through locally appropriate communication channels 2) Faire connaître l'étude par le biais des canaux de communication appropriés au niveau local |
| THigh (>15%) loss to follow-upTaux élevée (> 15%) de la perte au suivi | 1)  Reinforce importance of full follow-up in informed consent discussion and at study visits 1) Renforcer l'importance du suivi complet dans la discussion de consentement éclairé et à des visites d'étude  2)  Increase home visit staff 2) Augmentation du personnel de visites à domicile |
| Major discrepancies in validation and quality control of diagnostic test resultsDes divergences majeures dans la validation et le contrôle de la qualité des résultats des tests de diagnostic | 1)  Discuss with co-investigators to identify reason/s: poor equipment/lab supplies, personnel error, etc. 1) Discuter avec les co-chercheurs à identifier la raison / s: un mauvais équipement de fournitures de laboratoire /, l'erreur du personnel, etc  2)  Address cause with replacement of equipment/supplies, refresher training, personnel replacement if necessary, or other as appropriate 2) Adresser la cause avec le remplacement du matériel / des fournitures, la formation, le remplacement du personnel si nécessaire, ou d'autres, le cas échéant |
| Adverse events and/or protocol violationsLes événements indésirables et / ou des violations de protocole | Report to appropriate ethical committee and DSMB; prevention as appropriate through protocol amendment and/or personnel training Rapport au comité d'éthique approprié et aux moniteurs, la prévention le cas échéant par une modification du protocole et / ou la formation du personnel |

**17 Considérations éthiques et confidentialité**

Ethical approval will be sought from the WHO and from national/institutional review committees in all participating countries.  Written informed consent will be obtained from all participants as described in section 7.2.  Participants will be identified by coded study numbers rather than names in all data collection forms and electronic databases.  No individual identities will be used in any reports or publications resulting from the study. Only study staff and study investigators will have access to the information collected, for purposes of data entry and analysis.  At the time of informed consent for study participation, participants will be informed that participation in a research study may involve a loss of privacy; however, for this study no sensitive information will be collected, and only study personnel will have access to the information collected. L'approbation éthique sera sollicitée auprès de l'OMS et des comités d’éthique nationaux de tous les pays participants. Un consentement éclairé écrit sera obtenu de toutes les participantes tel que décrits dans la section 7.2. Les participantes seront identifiées par des codes d’étude plutôt que des noms dans tous les formulaires de collecte de données et dans les bases de données électroniques. Aucune identité individuelle ne sera utilisée dans toutes les publications issues de l'étude. Seule l’équipe de l’étude, le moniteur clinique, les inspecteurs, les auditeurs, le comité d’éthique ainsi que toute autorité reglementaire auront accès à l'information recueillie, à des fins de contrôle, de saisie et d'analyse. Au moment du consentement éclairé pour la participation à l'étude, les participantes seront informées que leur participation à l’étude est entièrement volontaire et que cela peut entraîner une perte de confidentialité, mais pour cette étude, aucune information sensible ne sera recueillie.

**18 BUDGET DE L’ETUDE**

| **Poste de dépense** | **Cout ($)** |
| --- | --- |
| Prise en charge des participantes (traitement, remboursement du transport) | 91 420 |
| Frais de supervision | 16 900 |
| Materiel de laboratoire | 20 181 |
| Equipe de recherche | 42 450 |
| Bureautique/Communication | 14 425 |
| Activités de la PCR | 36 020 |
| Autres dépenses imprévues | 7 500 |
| Overhead | 34 334 |
| Total | 263 231$ |

REFERENCES

**1.** Steketee RW, Nahlen BL, Parise ME, Menendez C. The burden of malaria in pregnancy in malaria-endemic areas. *Am J Trop Med Hyg.* Jan-Feb 2001;64(1-2 Suppl):28-35.

**2.** WHO/AFRO. *A strategic framework for malaria prevention and control during pregnancy in the African region.* Brazzaville: World Health Organization, Regional Office for Africa; 2004. AFR/MAL/O4/01.

**3.** Desai M, ter Kuile FO, Nosten F, et al. Epidemiology and burden of malaria in pregnancy. *Lancet Infect Dis.* Feb 2007;7(2):93-104.

**4.** Walker-Abbey A, Djokam RR, Eno A, et al. Malaria in pregnant Cameroonian women: the effect of age and gravidity on submicroscopic and mixed-species infections and multiple parasite genotypes. *Am J Trop Med Hyg.* Mar 2005;72(3):229-235.

**5.** Rogerson SJ, van den Broek NR, Chaluluka E, Qongwane C, Mhango CG, Molyneux ME. Malaria and anemia in antenatal women in Blantyre, Malawi: a twelve-month survey. *Am J Trop Med Hyg.* Mar 2000;62(3):335-340.

**6.** Newman RD, Hailemariam A, Jimma D, et al. Burden of malaria during pregnancy in areas of stable and unstable transmission in Ethiopia during a nonepidemic year. *J Infect Dis.* Jun 1 2003;187(11):1765-1772.

**7.** ter Kuile FO, Parise ME, Verhoeff FH, et al. The burden of co-infection with human immunodeficiency virus type 1 and malaria in pregnant women in sub-saharan Africa. *Am J Trop Med Hyg.* Aug 2004;71(2 Suppl):41-54.

**8.** Brabin BJ. An analysis of malaria in pregnancy in Africa. *Bull World Health Organ.* 1983;61(6):1005-1016.

**9.** Archibald HM. The influence of malarial infection of the placenta on the incidence of prematurity. *Bull World Health Organ.* 1956;15(3-5):842-845.

**10.** Jelliffe EF. Low birth-weight and malarial infection of the placenta. *Bull World Health Organ.* 1968;38(1):69-78.

**11.** Spitz AJ. Malaria infection of the placenta and its influence on the incidence of prematurity in eastern Nigeria. *Bull World Health Organ.* 1959;21:242-244.

**12.** Guyatt HL, Snow RW. The epidemiology and burden of Plasmodium falciparum-related anemia among pregnant women in sub-Saharan Africa. *Am J Trop Med Hyg.* Jan-Feb 2001;64(1-2 Suppl):36-44.

**13.** Guyatt HL, Snow RW. Impact of malaria during pregnancy on low birth weight in sub-Saharan Africa. *Clin Microbiol Rev.* Oct 2004;17(4):760-769, table of contents.

**14.** van Geertruyden JP, Thomas F, Erhart A, D'Alessandro U. The contribution of malaria in pregnancy to perinatal mortality. *Am J Trop Med Hyg.* Aug 2004;71(2 Suppl):35-40.

**15.** Uneke CJ. Diagnosis of Plasmodium falciparum malaria in pregnancy in sub-Saharan Africa: the challenges and public health implications. *Parasitol Res.* Feb 2008;102(3):333-342.

**16.** Rogerson SJ, Mkundika P, Kanjala MK. Diagnosis of Plasmodium falciparum malaria at delivery: comparison of blood film preparation methods and of blood films with histology. *J Clin Microbiol.* Apr 2003;41(4):1370-1374.

**17.** Cottrell G, Mary JY, Barro D, Cot M. Is malarial placental infection related to peripheral infection at any time of pregnancy? *Am J Trop Med Hyg.* Dec 2005;73(6):1112-1118.

**18.** Rogerson SJ, Mwapasa V, Meshnick SR. Malaria in pregnancy: linking immunity and pathogenesis to prevention. *Am J Trop Med Hyg.* Dec 2007;77(6 Suppl):14-22.

**19.** Mockenhaupt FP, Ulmen U, von Gaertner C, Bedu-Addo G, Bienzle U. Diagnosis of placental malaria. *J Clin Microbiol.* Jan 2002;40(1):306-308.

**20.** Mockenhaupt FP, Rong B, Till H, et al. Submicroscopic Plasmodium falciparum infections in pregnancy in Ghana. *Trop Med Int Health.* Mar 2000;5(3):167-173.

**21.** Adegnika AA, Verweij JJ, Agnandji ST, et al. Microscopic and sub-microscopic Plasmodium falciparum infection, but not inflammation caused by infection, is associated with low birth weight. *Am J Trop Med Hyg.* Nov 2006;75(5):798-803.

**22.** Guthmann JP, Ruiz A, Priotto G, Kiguli J, Bonte L, Legros D. Validity, reliability and ease of use in the field of five rapid tests for the diagnosis of Plasmodium falciparum malaria in Uganda. *Trans R Soc Trop Med Hyg.* May-Jun 2002;96(3):254-257.

**23.** Hopkins H, Bebell L, Kambale W, Dokomajilar C, Rosenthal PJ, Dorsey G. Rapid diagnostic tests for malaria at sites of varying transmission intensity in Uganda. *J Infect Dis.* Feb 15 2008;197(4):510-518.

**24.** de Oliveira AM, Skarbinski J, Ouma PO, et al. Performance of malaria rapid diagnostic tests as part of routine malaria case management in Kenya. *Am J Trop Med Hyg.* Mar 2009;80(3):470-474.

**25.** Houze S, Boly MD, Le Bras J, Deloron P, Faucher JF. PfHRP2 and PfLDH antigen detection for monitoring the efficacy of artemisinin-based combination therapy (ACT) in the treatment of uncomplicated falciparum malaria. *Malar J.* 2009;8:211.

**26.** WHO. *Guidelines for the treatment of malaria* 2006. WHO/HTM/MAL/2006.1108.

**27.** WHO/FIND/CDC/TDR. *Malaria rapid diagnostic test performance: Results of WHO product testing of malaria RDTs: Round 1 (2008).* http://apps.who.int/tdr/svc/publications/tdr-research-publications/rdt-performance 2009.

**28.** Leke RF, Djokam RR, Mbu R, et al. Detection of the Plasmodium falciparum antigen histidine-rich protein 2 in blood of pregnant women: implications for diagnosing placental malaria. *J Clin Microbiol.* Sep 1999;37(9):2992-2996.

**29.** Mankhambo L, Kanjala M, Rudman S, Lema VM, Rogerson SJ. Evaluation of the OptiMAL rapid antigen test and species-specific PCR to detect placental Plasmodium falciparum infection at delivery. *J Clin Microbiol.* Jan 2002;40(1):155-158.

**30.** Singer LM, Newman RD, Diarra A, et al. Evaluation of a malaria rapid diagnostic test for assessing the burden of malaria during pregnancy. *Am J Trop Med Hyg.* May 2004;70(5):481-485.

**31.** Malhotra I, Dent A, Mungai P, Muchiri E, King CL. Real-time quantitative PCR for determining the burden of Plasmodium falciparum parasites during pregnancy and infancy. *J Clin Microbiol.* Aug 2005;43(8):3630-3635.

**32.** Mockenhaupt FP, Bedu-Addo G, von Gaertner C, et al. Detection and clinical manifestation of placental malaria in southern Ghana. *Malar J.* 2006;5:119.

**33.** WHO/TDR. *Assessment of the safety of artemisinin compounds in pregnancy* 2006. WHO/CDS/MAL/20903.1094, WHO/GMP/TDR/Artemisinin/07.1.

**34.** WHO. *World Malaria Report 2008*: World Health Organization; 2008. WHO/HTM/GMP/2008.1.

**35.** Schultz LJ, Steketee RW, Macheso A, Kazembe P, Chitsulo L, Wirima JJ. The efficacy of antimalarial regimens containing sulfadoxine-pyrimethamine and/or chloroquine in preventing peripheral and placental Plasmodium falciparum infection among pregnant women in Malawi. *Am J Trop Med Hyg.* Nov 1994;51(5):515-522.

**36.** Verhoeff FH, Brabin BJ, Chimsuku L, Kazembe P, Russell WB, Broadhead RL. An evaluation of the effects of intermittent sulfadoxine-pyrimethamine treatment in pregnancy on parasite clearance and risk of low birthweight in rural Malawi. *Ann Trop Med Parasitol.* Mar 1998;92(2):141-150.

**37.** Parise ME, Ayisi JG, Nahlen BL, et al. Efficacy of sulfadoxine-pyrimethamine for prevention of placental malaria in an area of Kenya with a high prevalence of malaria and human immunodeficiency virus infection. *Am J Trop Med Hyg.* Nov 1998;59(5):813-822.

**38.** Shulman CE, Dorman EK, Cutts F, et al. Intermittent sulphadoxine-pyrimethamine to prevent severe anaemia secondary to malaria in pregnancy: a randomised placebo-controlled trial. *Lancet.* Feb 20 1999;353(9153):632-636.

**39.** Njagi JK, Magnussen P, Estambale B, Ouma J, Mugo B. Prevention of anaemia in pregnancy using insecticide-treated bednets and sulfadoxine-pyrimethamine in a highly malarious area of Kenya: a randomized controlled trial. *Trans R Soc Trop Med Hyg.* May-Jun 2003;97(3):277-282.

**40.** Kublin JG, Dzinjalamala FK, Kamwendo DD, et al. Molecular markers for failure of sulfadoxine-pyrimethamine and chlorproguanil-dapsone treatment of Plasmodium falciparum malaria. *J Infect Dis.* Feb 1 2002;185(3):380-388.

**41.** Alker AP, Mwapasa V, Purfield A, et al. Mutations associated with sulfadoxine-pyrimethamine and chlorproguanil resistance in Plasmodium falciparum isolates from Blantyre, Malawi. *Antimicrob Agents Chemother.* Sep 2005;49(9):3919-3921.

**42.** Omar SA, Adagu IS, Warhurst DC. Can pretreatment screening for dhps and dhfr point mutations in Plasmodium falciparum infections be used to predict sulfadoxine-pyrimethamine treatment failure? *Trans R Soc Trop Med Hyg.* May-Jun 2001;95(3):315-319.

**43.** Staedke SG, Sendagire H, Lamola S, Kamya MR, Dorsey G, Rosenthal PJ. Relationship between age, molecular markers, and response to sulphadoxine-pyrimethamine treatment in Kampala, Uganda. *Trop Med Int Health.* May 2004;9(5):624-629.

**44.** ter Kuile FO, van Eijk AM, Filler SJ. Effect of sulfadoxine-pyrimethamine resistance on the efficacy of intermittent preventive therapy for malaria control during pregnancy: a systematic review. *JAMA.* Jun 20 2007;297(23):2603-2616.

**45.** Gesase S, Gosling RD, Hashim R, et al. High resistance of Plasmodium falciparum to sulphadoxine/pyrimethamine in northern Tanzania and the emergence of dhps resistance mutation at Codon 581. *PLoS ONE.* 2009;4(2):e4569.

**46.** WHO. *Technical expert group meeting on intermittent preventive treatment in pregnancy (IPTp).* Geneva: World Health Organization; 11-13 July 2007 2007.

**47.** Greenwood B, Alonso P, ter Kuile FO, Hill J, Steketee RW. Malaria in pregnancy: priorities for research. *Lancet Infect Dis.* Feb 2007;7(2):169-174.

**48.** Brabin BJ, Wasame M, Uddenfeldt-Wort U, Dellicour S, Hill J, Gies S. Monitoring and evaluation of malaria in pregnancy - developing a rational basis for control. *Malar J.* 2008;7 Suppl 1:S6.

**49.** Council for International Organization of Medical Sciences. International Ethical Guidelines for Biomedical Research Involving Human Subjects. http://www.cioms.ch/frame_guidelines_nov_2002.htm. Accessed April, 2010.

**50.** White NJ. Intermittent presumptive treatment for malaria. *PLoS Med.* Jan 2005;2(1):e3.

**51.** Menendez C, Mayor A. Congenital malaria: the least known consequence of malaria in pregnancy. *Semin Fetal Neonatal Med.* Jun 2007;12(3):207-213.

**52.** Falade C, Mokuolu O, Okafor H, et al. Epidemiology of congenital malaria in Nigeria: a multi-centre study. *Trop Med Int Health.* Nov 2007;12(11):1279-1287.

**53.** Perrault SD, Hajek J, Zhong K, et al. Human immunodeficiency virus co-infection increases placental parasite density and transplacental malaria transmission in Western Kenya. *Am J Trop Med Hyg.* Jan 2009;80(1):119-125.

**54.** Mwangoka GW, Kimera SI, Mboera LE. Congenital Plasmodium falciparum infection in neonates in Muheza District, Tanzania. *Malar J.* 2008;7:117.

**55.** Global Forum for Health Research and WHO. *Research issues in sexual and reproductive health for low- and middle-income countries* 2007.

**56.** McCosker H. Undertaking sensitive research: issues and strategies for meeting the safety needs of all participants. *Forum: Qualitative Social Research.* Feb 2001 2001;2(1).

**57.** WHO/FIND/CDC. Methods manual for laboratory quality control testing of malaria rapid diagnostic tests. Aug 2008; version 5(a):Manual of SOPs for:lab-based quality control testing of malaria RDTs using stored dilutions of malaria parasites; and preparation of quality control samples from malaria parasite field collections. Available at: http://www.wpro.who.int/NR/rdonlyres/B5446BF5-BCFA-427D-B9FE-CEA57D36B92B/0/RDTQCMethodsManualV4final3WEBVERSION.pdf. Accessed 1 Sep, 2008.

**58.** WHO. Malaria Microscopy Quality Assurance Manual version 1. Feb 2009.
