# Supplementary material for: HRP2 and pLDH-Based Rapid Diagnostic Tests, Expert Microscopy, and PCR for Detection of Malaria Infection during Pregnancy and at Delivery in Areas of Varied Transmission: A Prospective Cohort Study in Burkina Faso and Uganda
Source: PLoS One. 2016 Jul 5;11(7):e0156954. doi: 10.1371/journal.pone.0156954 (PMC4933335; doi:10.1371/journal.pone.0156954)
Supplement: S1 Table — (DOCX) [file pone.0156954.s004.docx]

STROBE Statement—checklist of items that should be included in reports of observational studies

|  | Item No. | Recommendation | Page  No. | Relevant text from manuscript |
| --- | --- | --- | --- | --- |
| **Title and abstract** | 1 | (*a*) Indicate the study’s design with a commonly used term in the title or the abstract | 1 | prospective quasi experimental study in Loa PDR and Uganda |
|  |  | (*b*) Provide in the abstract an informative and balanced summary of what was done and what was found | 1 | Front-line HWs in two different malaria-endemic areas are able to correctly use prototype PCWs for quality control of malaria RDTs in routine health care. Results suggest that availability of PCWs can improve health workers’ confidence in RDT results, and that PCWs are both implementable and likely to benefit malaria diagnostic programmes. Future work should refine implementation strategies, and evaluate longer term impacts of PCWs on health worker behaviours and patient management. Lessons learned from malaria RDT and PCW implementation may be valuable in introducing other point-of-care diagnostic and quality-control tools. |
| Introduction | | | |  |
| Background/rationale | 2 | Explain the scientific background and rationale for the investigation being reported | 3 | Rapid diagnostic tests (RDTs) are widely used for malaria diagnosis, but the lack of quality control at point of care continues to restrict trust in test results. Prototype positive control wells (PCW) containing recombinant malaria antigens have been developed to identify poor quality RDT lots, but are yet to be used at scale. This study assessed front-line public health workers’ ability to use PCWs to detect degraded RDTs; the impact of PCW availability on RDT use and prescribing; and health workers’ perceptions of PCWs and preferred strategies for implementation. |
| Objectives | 3 | State specific objectives, including any prespecified hypotheses | 4 | *Training efficacy and effectiveness; all sites:*   1. To determine whether, after a half-day training, health workers can correctly prepare and interpret results of PCWs both at the time of initial training and after six months of routine use. 2. To determine whether health workers can use PCWs to effectively detect RDTs with poor sensitivity.   *Impact on use of RDTs and adherence to results:*   1. To assess the impact of PCW availability on health workers’ use of RDTs (rate, frequency, patient selection). 2. To assess the impact of PCW availability on health workers’ adherence to RDT results.   *Acceptability, feasibility, and cost implications:*   1. To collect information on the preferred strategies for implementation of PCWs (packaging, frequency/timing of recommended use). 2. To assess whether health workers find PCWs acceptable, feasible and valuable for use in routine clinical care. |
| Methods | | | |  |
| Study design | 4 | Present key elements of study design early in the paper | 4 | Two countries endemic malaria, prospectively quasi experimental with a one intervention arm and control arm where PCW were not introduced. We also a Pre-Post evaluation do measure impact of intervention. Qualitative methods through focus group discussions were used to collect information on acceptability of PCWs |
| Setting | 5 | Describe the setting, locations, and relevant dates, including periods of recruitment, exposure, follow-up, and data collection | 5-9 | The study was conducted from March to October 2013 in Salavan Province, southern Laos, and in Kiboga District, west-central Uganda. A total of 557 HWs participated in Laos (267) and Uganda (290); the majority were community-based HWs other Health Facility based HWK. After half day training at their respective centres, training, participants correctly performing the six key individual PCW steps under observation; performance was followed-up with two mid-term and end of study (six months later) evaluation |
| Participants | 6 | (*a*) *Cohort study*—Give the eligibility criteria, and the sources and methods of selection of participants. Describe methods of follow-up  *Case-control study*—Give the eligibility criteria, and the sources and methods of case ascertainment and control selection. Give the rationale for the choice of cases and controls  *Cross-sectional study*—Give the eligibility criteria, and the sources and methods of selection of participants |  | Study area selection criteria were: malaria RDTs meeting WHO procurement criteria, already in routine use in clinical care according to plans/programmes approved by the national malaria control authorities; representative sites in Africa and Asia; and local collaborators experienced in the conduct of operational research on malaria diagnosis.  The study was conducted at government-sponsored health facilities and at community or village health volunteers’ work stations where RDTs are used in routine patient care. |
|  |  | (*b*) *Cohort study*—For matched studies, give matching criteria and number of exposed and unexposed  *Case-control study*—For matched studies, give matching criteria and the number of controls per case |  | To assess the impact of PCW availability on RDT use, in each country routine clinical data from a neighbouring “control” area with similar climate, malaria epidemiology, health care infrastructure and RDT access but without PCWs (Sekong Province in Laos; Kyankwanzi District in Uganda) were obtained as aggregate summaries from the Ministry of Health (Laos) or from individual health facility and community worker logbooks (Uganda). |
| Variables | 7 | Clearly define all outcomes, exposures, predictors, potential confounders, and effect modifiers. Give diagnostic criteria, if applicable | 7 | After the initial training, health workers’ ability to correctly use PCWs was assessed: immediately after training, at the study mid-point about three months later, and at the end of the study six months after training. Outcomes: observed and scored individual participants on PCW performance and result interpretation. Second, at the study mid-point and end-point each health worker was individually presented with panels of reacted RDTs and asked to propose the correct actions if they obtained these results with a PCW. Third, the forms completed by health workers during their routine work over the study period were retrieved to determine: a) frequency of use of PCWs, b) results of RDTs tested with PCWs, c) interpretation of results, and d) any actions taken. |
| Data sources/ measurement | 8* | For each variable of interest, give sources of data and details of methods of assessment (measurement). Describe comparability of assessment methods if there is more than one group | *7* |  |
| Bias | 9 | Describe any efforts to address potential sources of bias | 8 |  |
| Study size | 10 | Explain how the study size was arrived at | 6 | A sample size of approximately 300 health workers in each of the two study areas was targeted. The goal was to include a representative sample of health workers who use malaria RDTs in routine practice, with recruitment of approximately 225 community workers in each country and the remainder being clinic staff. The target sample size represented approximately 3% to 5% of the community workers using RDTs in each country. |

Continued on next page

| Quantitative variables | 11 | Explain how quantitative variables were handled in the analyses. If applicable, describe which groupings were chosen and why | 9 | Training outcomes were presented as proportions and frequencies |
| --- | --- | --- | --- | --- |
| Statistical methods | 12 | (*a*) Describe all statistical methods, including those used to control for confounding | 9 | Comparisons between groups were made using Pearson’s Chi-square or Fisher’s exact test, while changes in performance between assessments were assessed using either McNemar or McNemar-Bowker test. Binary logistic regression was used to assess the association between age and amount of time the participant had been using RDTs on correctly preparing individual PCW steps and interpreting RDT results. Poisson regression was used to assess the association between age, facility and PCW use on the proportions of patients tested by RDT, positive by RDT and RDT-positive patients treated with an antimalarial. |
|  |  | (*b*) Describe any methods used to examine subgroups and interactions | 15 | FGD: In Laos, 84 participants took part in 11 semi-structured interviews and 11 FGDs. In Uganda, 119 participants participated in 29 interviews and 11 FGDs.  Most health workers reported that difficulties in performing the PCWs were generally minor and became easier with training and experience.  In general, PCWs were discussed by health workers as a way to confirm RDT quality and restore confidence in RDT results in some situations where doubts existed.  In both Laos and Uganda, among both clinic staff and community workers, one of the most frequently mentioned reasons for health workers to doubt RDT results was obtaining “too many” consecutive similar results when testing patients, especially consecutive negative results. Finally, some participants questioned whether PCWs could also be of poor quality |
|  |  | (*c*) Explain how missing data were addressed |  |  |
|  |  | (*d*) *Cohort study*—If applicable, explain how loss to follow-up was addressed  *Case-control study*—If applicable, explain how matching of cases and controls was addressed  *Cross-sectional study*—If applicable, describe analytical methods taking account of sampling strategy |  | There was minimal loss to follow-up due to floods in Loa PDR.  Impact of RDT deployment was assessed based on routine data that missed some variable, hence limitations mentioned in the discussion section. |
|  |  | (*e*) Describe any sensitivity analyses | 9 | Before the study began and at the end, sensitivity and proficiency testing of the PCWs was performed using reference methods in HTD London |
| Results | | | | |
| Participants | 13* | (a) Report numbers of individuals at each stage of study—eg numbers potentially eligible, examined for eligibility, confirmed eligible, included in the study, completing follow-up, and analysed | 10 |  |
|  |  | (b) Give reasons for non-participation at each stage |  |  |
|  |  | (c) Consider use of a flow diagram | Fig 2 | Included flow chart |
| Descriptive data | 14* | (a) Give characteristics of study participants (eg demographic, clinical, social) and information on exposures and potential confounders | 10 | Table 1 **:** Participating health workers: enrolment population and descriptive data |
|  |  | (b) Indicate number of participants with missing data for each variable of interest |  |  |
|  |  | (c) *Cohort study*—Summarise follow-up time (eg, average and total amount) | 10 |  |
| Outcome data | 15* | *Cohort study*—Report numbers of outcome events or summary measures over time | *21* | Table 2: Health worker performance of PCW with RDT, observed by study staff, immediately after training at start of study, at study midpoint three months after training, and at study end six months after training |
|  |  | *Case-control study—*Report numbers in each exposure category, or summary measures of exposure |  |  |
|  |  | *Cross-sectional study—*Report numbers of outcome events or summary measures |  |  |
| Main results | 16 | (*a*) Give unadjusted estimates and, if applicable, confounder-adjusted estimates and their precision (eg, 95% confidence interval). Make clear which confounders were adjusted for and why they were included | 22-23 | Tables 5a-c: Estimated marginal means (EMM) for rapid diagnostic testing (RDT), results, and antimalarial treatment in Lao PDR and Uganda for health facilities with and without positive control wells (PCW) and community health workers |
|  |  | (*b*) Report category boundaries when continuous variables were categorized |  |  |
|  |  | (*c*) If relevant, consider translating estimates of relative risk into absolute risk for a meaningful time period |  |  |

Continued on next page

| Other analyses | 17 | Report other analyses done—eg analyses of subgroups and interactions, and sensitivity analyses | 16 | In Laos, when aggregated data from clinic staff were compared between the PCW and control provinces, there were significant differences in the proportion of patients receiving an RDT in Salavan vs Sekong (p<0.001), and also between patient age groups within each province (p<0.001; Table 5a).  In Uganda, the proportion of patients tested was significantly higher in the control district (Kyankwanzi) than in Kiboga, and was also significantly higher in the pre-PCW period in both districts (Table 5b  Records for 39,882 patients seen by community health workers in Uganda were analysed (Table 5c). The odds of conducting an RDT were 1.61 (95% CI: 1.49 – 1.74) times higher for the post-PCW period compared to the pre-PCW period in both districts. |
| --- | --- | --- | --- | --- |
| Discussion | | | | |
| Key results | 18 | Summarise key results with reference to study objectives | 18 | A total of 557 HWs participated in Laos (267) and Uganda (290); the majority were community-based HWs. After training, most (88% to ≥99%) participants correctly performed the six key individual PCW steps under observation; performance was generally maintained during the six-month study period. Most (89%) HWs recommended an appropriate action on PCW results. Nearly all (97%) reported a correct action on PCW result at the routine work site. In Uganda, where data for 127,775 individual patients were available, PCW introduction in health facilities was followed by an increase in antimalarial prescribing on RDT-negative results for young children (from 2.4% to 5.6%) but a decrease for other patients (from 4.7% to 1.9%); whereas antimalarial prescribing increased in the control district (no PCWs. Among community-based HWs, after PCW introduction antimalarial treatment of RDT-negatives declined by an estimated 12.2% (p<0.05), compared to an increase of almost 40% in the control district (p<0.05). Qualitative data revealed PCWs as a way to confirm RDT quality and restore confidence in RDT results. |
| Limitations | 19 | Discuss limitations of the study, taking into account sources of potential bias or imprecision. Discuss both direction and magnitude of any potential bias | 18 | Health workers knew that they were participating in research, so the Hawthorne effect may have influenced their PCW performance under observation as well as records kept during routine work. Keeping written records appeared to be challenging for some study participants, especially in Laos where some records with missing data were excluded from analysis. This observation reflects the challenges of conducting research among front-line health workers in malaria-endemic areas (and also highlights one of the challenges encountered when health care systems must rely on staff with limited education). More PCWs were used per health worker in Uganda than in Laos, perhaps at least in part because the RDTs in the Uganda study area were more freely available. Patient-level data on RDT use and antimalarial prescribing was only available in Uganda, so the effects seen there could not be compared with data from Laos |
| Interpretation | 20 | Give a cautious overall interpretation of results considering objectives, limitations, multiplicity of analyses, results from similar studies, and other relevant evidence | 19 | Results suggest that availability of PCWs can improve health workers’ confidence in RDT results, and that PCWs are both implementable and likely to benefit malaria diagnostic programmes. Lessons learned from malaria RDT and PCW implementation may be valuable in introducing other point-of-care diagnostic and quality-control tools. |
| Generalisability | 21 | Discuss the generalisability (external validity) of the study results | 20 | We had study had a wide range of quality control measures that ensured that the results are accurate. The sample size and wide |
| Other information | |  | | |
| Funding | 22 | Give the source of funding and the role of the funders for the present study and, if applicable, for the original study on which the present article is based | 22 | This study was supported by the Foundation for Innovative New Diagnostics (FIND) with funds from the Bill and Melinda Gates Foundation and the UK Department for International Development –DFID . The Funders did not have any role in the presented study. |

*Give information separately for cases and controls in case-control studies and, if applicable, for exposed and unexposed groups in cohort and cross-sectional studies.

**Note:** An Explanation and Elaboration article discusses each checklist item and gives methodological background and published examples of transparent reporting. The STROBE checklist is best used in conjunction with this article (freely available on the Web sites of PLoS Medicine at http://www.plosmedicine.org/, Annals of Internal Medicine at http://www.annals.org/, and Epidemiology at http://www.epidem.com/). Information on the STROBE Initiative is available at www.strobe-statement.org.
